# Zinc *N*,*N*-bis(2-picolyl)amine Chelates Show Substitution-Dependent Cleavage of Phosphodiesters in Models as Well as of PNAzyme-RNA Bulges

**DOI:** 10.3390/molecules29092123

**Published:** 2024-05-03

**Authors:** Søren W. Svenningsen, Olivia Luige, Zeyed Abdulkarim, Roger Strömberg, Nicholas H. Williams

**Affiliations:** 1 Department of Chemistry, University of Sheffield, Richard Roberts Building, Sheffield S3 7HF, UK; swsvenningsen@hotmail.com (S.W.S.); zeyed.abdulkarim@gmail.com (Z.A.); 2 Department of Biosciences and Nutrition, Karolinska Institutet, Neo, Halsovagen 9, 14157 Huddinge, Sweden; olivialuige@gmail.com; 3 Department of Laboratory Medicine, Karolinska Institutet, ANA Futura, Nobels alle 8 B, 14152 Huddinge, Sweden

**Keywords:** PNAzyme, metal chelate, RNA substrate, RNA bulge, phosphodiester cleavage

## Abstract

PNAzymes are a group of artificial enzymes which show promising results in selective and efficient cleavage of RNA targets. In the present study, we introduce a series of metal chelating groups based on *N*,*N*-bis(2-picolyl) groups (parent, 6-methyl and 6-amino substituted) as the active sites of novel PNAzymes. An improved synthetic route for the 6-amino analogues is described. The catalytic activity of the chelating groups for cleaving phosphodiesters were assessed with the model substrate 2-hydroxypropyl p-nitrophenyl phosphate (HPNPP), confirming that the zinc complexes have the reactivity order of parent < 2-methyl < 2-amino. The three ligands were conjugated to a PNA oligomer to form three PNAzymes which showed the same order of reactivity and some sensitivity to the size of the RNA bulge designed into the catalyst–substrate complex. This work demonstrates that the kinetic activity observed for the model substrate HPNPP could be translated onto the PNAzymes, but that more reactive Zn complexes are required for such PNAzymes to be viable therapeutic agents.

## 1. Introduction

The ability to selectively cleave and manipulate the nucleic acid sequences of the RNA and DNA that constitute genetic material is central to molecular biology research, medical analysis and the potential treatment of genetic diseases through gene editing or silencing.

DNA restriction enzymes are an invaluable tool for molecular biology research due to their capacity to recognize short DNA sequences and to degrade them in a defined fashion. Creating reagents capable of site-specific cleavage of RNA target sequences could provide a further valuable tool. This type of cleavage is believed to be achieved in the medical applications of synthetic oligonucleotides, where hybridisation with a target sequence leads to the recruitment of natural enzymes RNase H or an RNA-induced silencing complex that catalyses the cleavage of the target [1]. This approach is limited by the requirement that the substrate can be recognized by the enzyme, which limits the features that can be introduced to enhance the properties of the oligonucleotide in a biological context, such as stability to nonspecific degradation, enhanced hybridization with complementary sequences and cellular uptake [2]. Thus, a desirable alternative is to design oligonucleotides that have the inherent capability of cleaving target sequences as well as recognizing them.

Such artificial ribonucleases have been created and have been shown to catalytically cleave their targets in a sequence-selective manner. The target selectivity has been achieved using oligonucleotide-based sequences (OBANs), such as 2′-*O*-methyloligoribonucleotides [3,4,5] and peptide nucleic acid sequences (PNAzymes) [6,7]. The selectivity for a target is designed in such a way that a bulge is created at the position to be cleaved which can be accessed via a pendant functionality. This functionality is usually a metal chelating ligand. Cleavage leaves a product state where the target strands hybridise less effectively to the catalyst, and so, turnover is assured. The creation of a bulge also provides opportunities to create more reactive target sites by controlling the conformation of the RNA backbone once hybridization has occurred.

Notable successes have been reported using neocuproine as the ligand which can coordinate a metal ion and form a species reactive enough to catalyse the cleavage of the RNA target, both with 2′-*O*-Methyloligoribonucleotide-based systems [3,4,5] and with PNAzymes [6,7]. Most of the reactive species to date with high site selectivity use Cu(II) as the metal ion [8,9], but this presents toxicity challenges in biological systems. Zn(II) has recently been used, and with similar or even slightly higher activity [10,11]. However, further search for alternative chelates is desirable, especially if therapeutic applications are going to be realized; and this development will be assisted by an accompanying understanding of the mechanistic principles that govern activity.

One group of metal chelating ligands which has been described in the literature for a variety of purposes is the *N*,*N*-bis(2-picolyl)amine head group. Metal ion complexes of these tridentate ligands have found applications in molecular imaging, phosphate binding and phosphodiester cleavage [12,13,14,15,16,17]. Of particular relevance to developing artificial nucleases, it has been shown that by modifying the second coordination sphere of the metal ion with 6-amino substituents on the pyridyl ring provides considerably enhanced activity towards phosphate diester cleavage over the parent pyridyl group [18,19,20]. These properties have been established primarily using artificial analogues for RNA, such as 2-hydroxypropyl para-nitrophenyl phosphate (HPNPP). Despite the benefits of this substitution, the synthetic preparation of these derivatives has been inconvenient and typically involves low yields and difficult purification of key intermediates [19,21].

**Figure 1:**
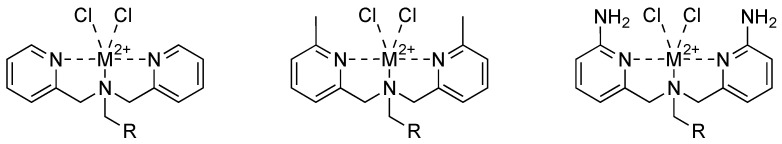
Structures of metal chelating *N*,*N*-bis(2-picolyl)amine groups used in this study. R indicates the linker site.

In the present study, we introduce the metal chelating *N*,*N*-bis(2-picolyl)amine moiety as the catalytic group in a novel group of PNAzymes. Three different PNAzymes that differ in the substituent at the 2′ position of the pyridine rings (Figure 1) were synthesised. We establish a more convenient synthetic methodology to incorporate the 2-aminopyridyl unit and compare the catalytic activity of the parent zinc complexes on the model substrate HPNPP with the catalytic activity they display in the macromolecular PNAzyme systems that cleave RNA. The three PNAzymes synthesized were studied in combination with different RNA substrates to create both 3- and 4-nucleotide bulges as the target cleavage site.

## 2. Results and Discussion

### 2.1. Synthesis

To construct bis(2-picolyl)amines, the key building block has a 2-methylene substituent with a leaving group on the pyridine core. When the 6-substituent is H or Me, these building blocks are readily accessible and the corresponding *N*,*N*-bis(2-picolyl)amine ligands can be synthesised conveniently. However, although unsymmetrical 2,6-disubstituted pyridines are widespread building blocks in many fields of chemistry, there is a lack of efficient synthetic strategies when one of these substituents is an amino group. This has been a limitation in the preparative scale production of the intermediates for making metal ion chelating ligands that contain the 2-aminopicolyl structure, despite the favourable impact that these have on binding and reactivity [12,13,14,15,16,17,18,19,20].

The most common previous strategies have focused on the functionalisation of a methyl group in 2-methyl-6-amino-pyridine, after the amino group has been protected. The direct bromination of tert-butyl(6-methylpyridin-2-yl)amide leads to a variety of brominated species that are difficult to separate and a product that requires deprotection under harsh conditions. Using tert-butyl(6-methylpyridin-2-yl)carbamate alleviates the final deprotection but suffers from similarly intractable problems in product mixtures [22,23]. These methods are difficult to use conveniently on a preparative scale.

**Scheme 1:**
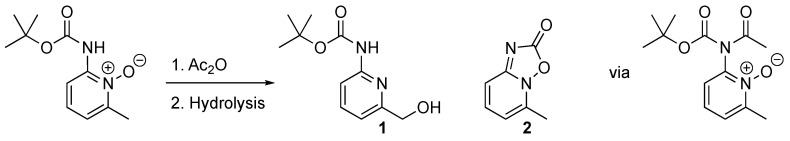
Functionalisation of the 6-methyl position via the Boekelheide reaction.

An alternative to bromination of the methyl group is the Boekelheide reaction in which the *N*-oxide of the pyridine rearranges to form a 2-hydroxymethyl in a much more selective reaction. We implemented this approach by oxidising tert-butyl(6-methylpyridin-2-yl)carbamate to the corresponding *N*-oxide and reacting it with acetic anhydride. The ensuing isomerisation is followed by hydrolysis to produce the tert-butyl(6-(hydroxymethyl)pyridin-2-yl)carbamate **1**. This process introduces specificity to the functionalization process, whilst retaining a convenient protecting group on the amino functionality, and is conveniently carried out. However, the process is hampered by a competing transfer of the acyl group to the *N*-oxide, leading to a heterocyclic side product **2** as illustrated in Scheme 1. This competition is sufficiently efficient (~50% yield for **2**) to severely reduce the yield of the desired intermediate (~20% yield for **1**). This side reaction can be prevented by methylating the 2-amino group (leading to ~75% yields for the rearrangement) but leads to an N-methyl substituent rather than the parent amino group that we desired in this work, to be able to use the corresponding Me analogue as a steric control for the amino group in our kinetic studies.

As an efficient synthesis of (6-aminopyridin-2-yl)methanol was a critical step in the synthesis of the amine substituted pyridines we required, with the alcohol serving as a precursor for introducing a halide or pseudo halides, we explored a new way to introduce amines in the 2-position of the pyridine systems as described by Yin et al. [24]. This strategy is based on the direct amination of pyridines with tert-butylamine by activation of the 2 and 4 positions through reacting the pyridine *N*-oxide with tosic anhydride. However, we were unable to synthesize (6-aminopyridin-2-yl)methanol starting from 2-(acetoxymethyl)pyridine 1-oxide using this methodology, so we reconsidered our strategy.

**Scheme 2:**
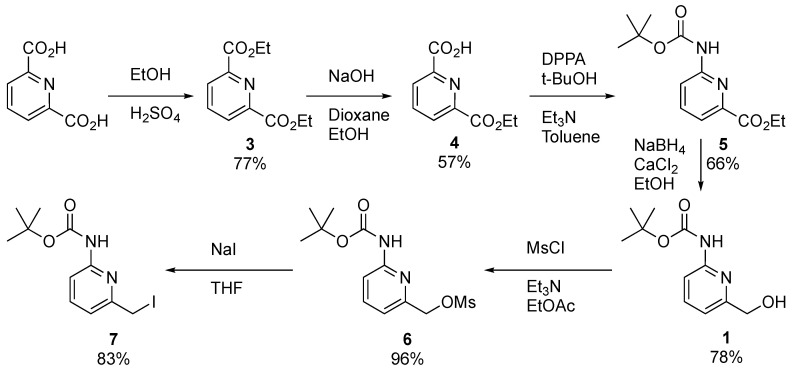
Pathway for the synthesis of protected 6-amino pyridylmethyl alkylating agent.

We chose to follow the strategy of Morihiro Mitsuya et al. [25] in synthesising boc protected 2-amino building blocks in their search for a novel Muscarinic M3 Receptor Antagonist. Starting from dipicolinic acid, both carboxylic acids were converted into ethyl esters **3** via a Fischer esterification in ethanol. One of the esters was selectively hydrolysed in good yield to produce the mono carboxylic acid **4**. The carboxylic acid was converted into the boc protected 2-amino-pyridine in one step with diphenyl phoshoryl azide in the presence of tert-butyl alcohol resulting in **5**, via a Curtius rearrangement. Reduction of the ethyl ester with sodium borohydride in the presence of calcium chloride in ethanol resulted in alcohol **1**. The alcohol was then turned into an alkylating agent by mesylation of the alcohol with mesyl chloride in ethyl acetate, resulting in **6**. The mesylate did not show sufficient alkylating properties towards the amines we used, and, therefore, the mesylate was converted to iodide **7** with sodium iodide in tetrahydrofuran. Similarly inefficient alkylation was observed when the mesylate of (6-methylpyridin-2-yl)methanol was used to create the 2-methyl derivatives of the ligands we studied, and for this reason, the mesylate of the intermediate was also turned into the alkyl iodide. Overall, this synthetic route (Scheme 2) provides an efficient and scalable method to introduce the protected 2-amino pyridylmethyl substituent into ligands suitable for binding metal ions.

**Scheme 3:**
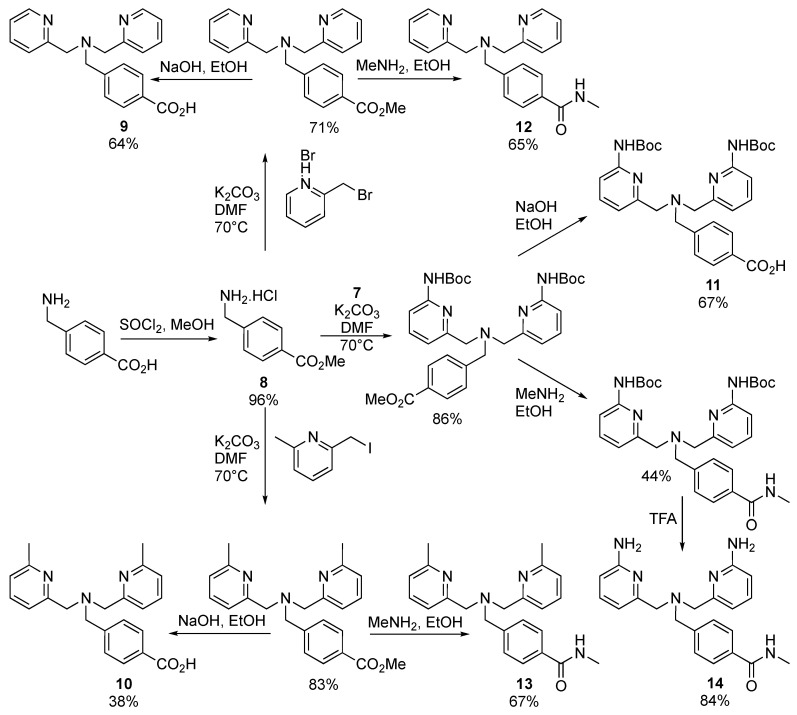
Synthetic scheme for the synthesis of the *N*,*N*-bis(2-picolyl)amine ligands.

The synthesis of the *N*,*N*-bis(2-picolyl)amine and its 6-methyl and 6-amino derivatives was based on the double alkylation of **8** with commercially available 2-(bromomethyl)pyridine hydrobromide or the respective alkyl iodides in dimethyl formamide. The synthetic procedure is shown in Scheme 3 and successfully provided **9**, **10** and **11,** which contain a carboxylic acid moiety for conjugation to an amine site in a PNA oligomer. The N-methyl amide derivatives of these compounds (**12**, **13** and **14**) were synthesised to allow the reactivity of the corresponding zinc complexes towards HPNPP cleavage to be measured and allowed the X-ray analysis of the corresponding zinc complexes.

**Scheme 4:**
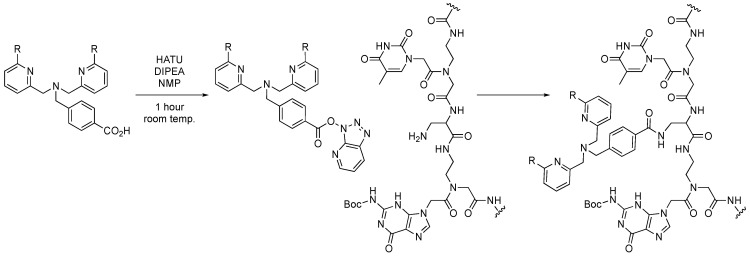
Conjugation of ligand **12**–**14** to the central part of the PNA sequence.

The carboxylic acid derivatives **9**, **10** and **11** were conjugated to PNA in order to synthesise the PNAzymes. The PNA oligomer was synthesized using a microwave-assisted peptide synthesizer Biotage^®^ Initiator+ Alstra^™^ (Uppsala, Sweden) as described in the literature [8,9,10,11]. Selective deprotection of the central 4-methyltrityl protected amine with 0.1% TFA in DCM, as previously reported [8,9], resulted in the free primary amine for conjugation to the *N*,*N*-bis(2-picolyl)amine ligands. The three ligands were conjugated to the PNA sequence via standard HATU peptide chemistry (Scheme 4) and the PNA was cleaved from the solid support and fully deprotected in one step with TFA. Purification via HPLC resulted in the final PNAzymes (PNAzyme 1, H; PNAzyme 2, Me and PNAzyme 3, NH_2_) which were characterized via mass analysis with MALDI-TOF MS and used for the kinetic studies. Concentrations of the purified PNAzymes were determined spectrophotometrically with nearest-neighbour approximation [8,9,10,11].

### 2.2. X-ray Structures

**Figure 2:**
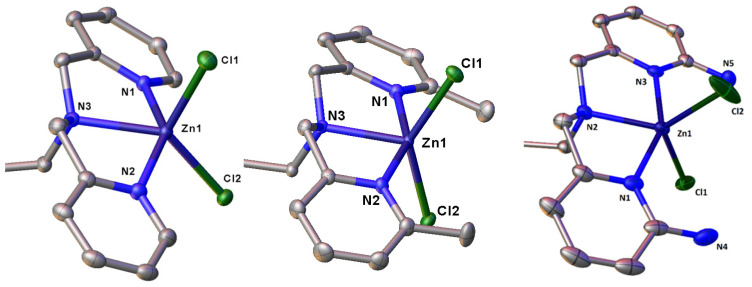
Comparison of the binding site to the Zn ion of the N,N-bis(2-picolyl) amine ligands **12**–**14** complexed with ZnCl_2_. **12.**ZnCl_2_ (**left**), **13.**ZnCl_2_ (**middle**) and **14.**ZnCl_2_ (**right**).

Crystals of the zinc complexes formed from **12, 13** and **14** and zinc chloride suitable for X-ray analysis were obtained via crystallisation from methanol and diethyl ether, allowing for the slow diffusion of ether into the methanol solution of the zinc complexes. All three ligands bind Zn(II) ions in a tridentate manner as confirmed with the X-ray analysis of **12**–**14**. The differing substituents at the six position of the pyridine lead to variations in geometry and bond lengths between the ligand and the zinc ion. Adding a methyl group leads to longer pyridyl–zinc distances (2.27 cf. 2.17 Å), which is compensated by a reduction in the zinc–tertiary amino distance (2.15 cf. 2.20 Å). The geometry of the coordination sphere around the zinc ion also becomes more trigonal bipyramidal, compared to a square based pyramid in the unsubstituted analogue. Presumably, this is driven by the steric congestion of the 2-methyl substituents. Searching the Cambridge Crystallographic Database reveals that the only comparable complex with the methyl substituent is also trigonal bipyramidal which has similar bond lengths to the coordinating nitrogen atoms (Table 1 and Figure 2). It also shows a small number of similar complexes for the parent pyridyl unit, showing both square based pyramidal and trigonal bipyramidal structures. In the trigonal bipyramidal geometry, the pyridyl–zinc distances are rather shorter with a range of 2.10 to 2.12 Å and a concomitant increase in the distance to the tertiary amine to 2.38 or 2.40 Å. When the amino groups are present, the trigonal bipyramidal geometry is retained, but the distances change. Converting methyl to amino reduces the pyridyl-zinc distances (2.19 cf. 2.27 Å), but the zinc–tertiary amine distance remains similar (2.14 cf. 2.15 Å). We note that similar geometries are accessible for all the complexes, and that the sum of the distances are shortest for the amino substituted complex and longest for the methyl substituted complex. Tentatively, we expect that weaker interactions by the ligand would lead to a more Lewis acidic zinc centre; tighter coordination would be expected to reduce the Lewis acidity. Thus, the effects of the substitutions may have a different balance of effects in modulating the catalytic activity of the zinc complexes.

### 2.3. Titration Data

The p*K*_a_s of the ligands were measured via potentiometric titration under the same conditions used for the kinetic analysis (25 °C, *I* = 0.1 M NaCl), along with the binding constant for the coordination of Zn(II) ions. In the zinc aqua complex, the water molecules can be deprotonated, and these are included in the model for the titration of the ligand in the presence of one equivalent of zinc ions. These data are consistent with the parameters for similar compounds [18,20]. The lower formation constant for **13.**Zn is consistent with the greater ligand–zinc distances noted in the structural analysis, suggesting less effective coordination and rationalising the greater Lewis acidity for water coordinated to the zinc ion. The found p*K*_a_ values are listed in Table 2.

### 2.4. HPNPP Cleavage

**Scheme 5:**
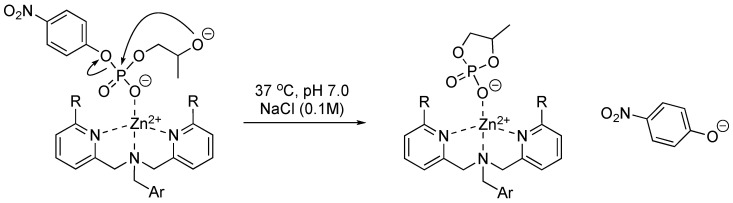
Suggested reaction mechanism for the ligand-Zn-mediated cleavage of the model substrate HPNPP.

**Figure 3:**
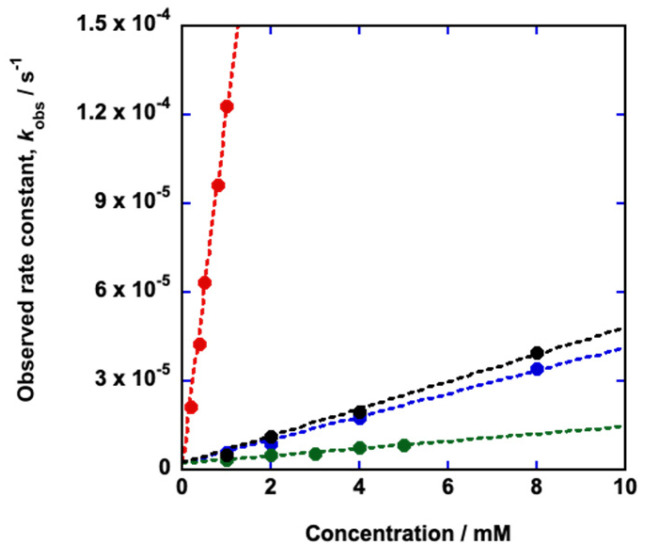
Dependence of observed rate constants for reaction of HPNPP on the concentration of ZnCl_2_ (black), **12.**Zn (green), **13.**Zn (blue) and **14.**Zn (red). pH 7.0, *I* = 0.1 M (NaCl) and 37 °C.

The methyl amide derivatives **12**–**14** were used to study the effect that these complexes have on the cleavage of HPNPP, a model RNA analogue that has been widely used in the literature [26,27]. These kinetic experiments were carried out at a pH of 7.0 (HEPES buffer 50 mM) in NaCl (0.1 M) at 37 °C (Scheme 5). The ligand and metal ion were mixed in a 1:1 ratio so that the complex formed in situ. These observed rate constants were plotted against the concentration of zinc complexes in a second order plot to determine their second order rate constants. As shown in Figure 3, a linear correlation between complex concentration and the observed rate constants was observed confirming the first order dependence of the reaction on the concentration of zinc complexes in each case. The relative rate constants of the three complexes compared to zinc chloride in the absence of an added ligand was calculated and shown in Table 3.

These data are consistent with previous reports for related complexes containing these picolyl ligands [17,18]. The unsubstituted complex (**12**.Zn) is less reactive than zinc chloride in solution, showing that ligation deactivates the ability of the zinc to cleave HPNPP. **14**.Zn is about 26-fold more reactive than the same concentration of zinc chloride, and about 100-fold more reactive than the unsubstituted ligand, demonstrating the impact of the 2-amino substituents. **13**.Zn reflects the steric effect of the 2-amino groups, but with very different electronic and hydrogen bonding potential, and leads to a complex which is comparable to zinc chloride, but about 30-fold less reactive than the amino substituted compound. In this context, the impact of the amino substituent does not appear to be primarily steric in character and may be due to direct hydrogen bonding interactions between the ligand and the bound substrate.

### 2.5. PNAzyme Reactivity

**Figure 4:**
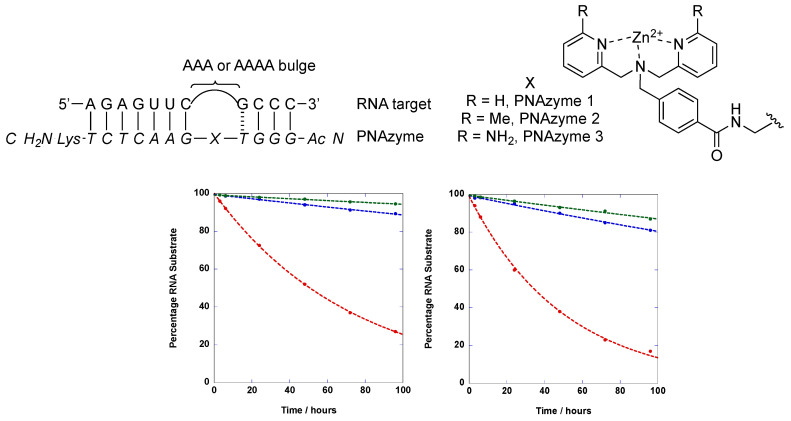
(**Top**): PNAzyme-RNA complexes studied. (**Bottom**): as in the legend now. Cleavage of the AAA (**left**) and AAAA (**right**) bulge mediated by 4 µM PNAzyme in the presence of 100 µM ZnCl_2_ at pH 7 and 37 °C. PNAzyme 1 (green), PNAzyme 2 (blue) and PNAzyme 3 (red).

The reactivity of the PNAzymes were studied in a similar manner to that previously reported for the neocuproine system [3,4,5,6,7,10,11]. Both AAA and AAAA bulges were designed into the PNAzyme:RNA duplex to form the cleavage target (Figure 4). A first order rate equation was fitted to the decrease in concentration of the substrate to produce the rate constants shown in Table 4.

The data show that there is a similar increase in activity as the substituents are changed from H to methyl to amino. The ratio is smaller, with the amino substituted ligand about 10-fold more reactive than the methyl substituted ligands, which is approximately 2-fold more reactive than the unsubstituted ligand. In all these experiments, the excess of zinc chloride used means that there is potentially a competing reaction from the zinc aqua ions; under these conditions, this reaction would be the dominant pathway for HPNPP cleavage. The observed difference in rates of reaction suggests that zinc complexes are more reactive than the zinc aqua ion, although it is possible that a significant fraction of the observed cleavage is due to the zinc aqua ions.

Previous data [6,7,10,11] suggest that a complex formed from the same target but with a 2′-*O*-Methyloligoribonucleotide as the basis to create an AAA bulge (i.e., using a modified RNA as the recognition feature of the catalyst) is cleaved with a rate constant of ca 1 × 10^−6^ s^−1^ in the presence of 100 µM zinc chloride [3,4,5,6,7]. This is comparable or slightly faster than the rate constants observed with the parent and methyl derivatives. More directly comparable data are obtained from the reaction in the presence of 100 µM zinc ions and PNA that does not contain a chelating ligand, where the rate of cleavage is estimated as slower than 1 × 10^−6^ s^−1^, and so, in this structure, cleavage promoted by Zn(II) ions in solution appears to be slower than in the 2′-*O*-Methyloligoribonucleotide systems [10,11]. If the observed rate constant has a significant contribution from free Zn(II) ions in solution, the relative rates of the complexes will be greater than reported in Table 4.

Considering the substrate with the AAA bulge, if we assume that the rate constant observed for PNAzyme 1 is primarily due to the background contribution from Zn(II) in solution then the ratio between Zn(II) background:PNAzyme 2:PNAzyme 3 will be 1:1.3:26. The 20-fold ratio between PNAzymes 2 and 3 is slightly lower than the 30-fold ratio measured for the reactivity of these complexes with HPNPP. This analysis assumes an insignificant contribution from the Zn(II) complex in PNAzyme 1 to the observed reaction, which would imply a larger difference in reactivity relative to the other derivatives. If 10% of the observed reactivity is due to the complex, then the relative rates with PNAzymes 2 and 3 would be 13- and 260-fold, respectively—somewhat larger than for the reactions with HPNPP.

Alternatively, if the same relative rate between the contribution from –H and –NH_2_ (1:98) from the HPNPP study is applied to the PNAzymes, the relative rates between PNAzyme 3 and PNAzyme 1 will be 27:0.28. If the contribution from PNAzyme 1 equals 0.28, the contribution of free Zn(II) would be 1 − 0.28 = 0.72. When this contribution is subtracted from the original observed relative rate constants (–Me: 2.3 − 0.72 = 1.58 and –NH_2_: 27 − 0.72 = 26.3), a new set of relative rate constants are generated. If these values are compared with the slowest system, PNAzyme 1, the relative rates for the contribution of –H, –Me and –NH_2_ are 1:6:98. The derived ratio between PNAzymes 1 and 2 is reasonably close to the value of 3.3 observed for HPNPP.

If the same principles were applied to the PNAzyme data for the –AAAA– bulge, the relative rates would be 1:0.6:14, giving a ratio of 23 between PNAzymes 2 and 3 and relative ratios of 6 and 140 with PNAzyme 1. Assuming a ratio of 98 between PNAzyme 1 and 3 leads to a derived ratio between PNAzymes 1 and 2 of 5.

These considerations suggest that the relative rate ratios are in reasonably good agreement in the different systems. An additional factor which could justify a simple comparison of the observed rate constants, assigning them to the activity of the relevant PNAzymes, would be the likely effective molarity of the conjugated complex. This has been estimated at around 1 mM, which is typical for a system where there is not a tightly controlled interaction between the target site and the complex—and where there are unlikely to be unfavourable interactions preventing the association of the complex with the target. This would mean the complex is effectively in a significant excess relative to the solution concentration of the zinc ions, compensating for the likely lower intrinsic reactivity. This simple analysis would lead to smaller rate ratios between the different complexes compared to their impact on HPNPP cleavage.

All of these analyses suggest that, for these complexes, a similar set of reactivity ratios are observed for the structural variation in the ligands for both substrates, implying that, in this case, HPNPP studies provide a useful indication of the features that can confer enhanced activity in more sophisticated constructs. The most likely rationale is that the interaction of these zinc complexes with the substrate is primarily through the phosphoryl group, where the impact on both substrates (which differ markedly in their nucleophilic and leaving group elements) is expected to be similar. The differences observed may be due to differing transition state structures for the rate limiting step in each case. This is likely to be early in the reaction of HPNPP (which may react through a single step), whereas the departure of the leaving group might be expected to be rate limiting for the RNA substrates and to have a late transition state.

## 3. Materials and Methods

### 3.1. Synthesis of Chelating Ligands

Described in Supplementary Materials.

### 3.2. Synthesis of PNA Conjugates 

#### 3.2.1. PNAzyme 3

The resin PNA (5.7 mg; 1 eq) was placed in a reactor containing a bottom filter, and the MTT group was removed via repetitive washes of the resin with TFA/DCM (1:99) solution. The wash was continued for approximately 1 min until a clear and colourless cleaved phase was observed (bright yellow becoming colourless). The resin was then washed with DCM, NMP and NMP + NMM followed by NMP. The ligand **9** (9.5 mg; 0.017 mmol 25 eq) and HATU (5.9 mg; 0.016 mmol; 23 eq) were taken up in NMP (80 µL), and DIPEA (5.4 µL; 46 eq) was added. The preactivation was left to react for 1 h at RT. The preactivated solution was added to the washed and deprotected resin and left to react for 1.5 h at RT. The resin was then washed with NMP (10 × 2 mL) and DCM (10 × 2 mL). The PNA conjugate was cleaved off the solid support by use of a cleavage mixture containing TFA/H_2_O/TIS (95:2.5:2.5) (1 mL) for 90 min at RT. The cleavage mixture was collected and evaporated under an N_2_ stream. Water (1.5 mL) was added, and the water phase was washed with Et_2_O (2 × 1.5 mL). The water phase was then evaporated *in vacuo* before being taken up in MilliQ water (Billerica, MA, USA) and purified via RP-HPLC. MALDI: *m*/*z* [M+H]^+^ calcd for [C_150_H_188_N_74_O_38_+H^+^]: 3635.5160; Found: 3637.4154. 

#### 3.2.2. PNAzyme 2

The resin PNA (5.8 mg; 1 eq) was placed in a reactor containing a bottom filter, and the MTT group was removed via repetitive washes of the resin with TFA/DCM (1:99) solution. The wash was continued for approximately 1 min until a clear and colourless cleaving phase was observed (bright yellow becoming colourless). The resin was then washed with DCM, NMP and NMP + NMM followed by NMP. The ligand **8** (6.3 mg; 0.017 mmol; 25 eq) and HATU (5.9 mg; 0.016 mmol; 23 eq) were taken up in NMP (80 µL), and DIPEA (5.4 µL; 46 eq) was added. The preactivation was left to react for 1 h at RT. The preactivated solution was added to the washed and deprotected resin and left to react for 1.5 h at RT. The resin was then washed with NMP (10 × 2 mL) and DCM (10 × 2 mL). The PNA conjugate was cleaved off the solid support by use of a cleavage mixture containing TFA/H_2_O/TIS (95:2.5:2.5) (1 mL) for 90 min at RT. The cleavage mixture was collected and evaporated under an N_2_ stream. Water (1.5 mL) was added, and the water phase was washed with Et_2_O (2 × 1.5 mL). The water phase was then evaporated *in vacuo* before being taken up in MilliQ water and purified via RP-HPLC. MALDI: *m*/*z* [M+H]^+^ calcd for [C_152_H_190_N_72_O_38_+H^+^]: 3633.5255; Found: 3634.2500.

#### 3.2.3. PNAzyme 1

The resin PNA (5.6 mg; 1 eq) was placed in a reactor containing a bottom filter, and the MTT group was removed via repetitive washes of the resin with TFA/DCM (1:99) solution. The wash was continued for approximately 1 min until a clear and colourless cleaving phase was observed (bright yellow becoming colourless). The resin was then washed with DCM, NMP and NMP + NMM followed by NMP. The ligand **7** (5.6 mg; 0.017 mmol; 25 eq) and HATU (5.9 mg; 0.016 mmol; 23 eq) were taken up in NMP (80 µL), and DIPEA (5.4 µL; 46 eq) was added. The preactivation was left to react for 1 h at RT. The preactivated solution was added to the washed and deprotected resin and left to react for 1.5 h at RT. The resin was then washed with NMP (10 × 2 mL) and DCM (10 × 2 mL). The PNA conjugate was cleaved off the solid support by use of a cleavage mixture containing TFA/H_2_O/TIS (95:2.5:2.5) (1 mL) for 90 min at RT. The cleavage mixture was collected and evaporated under a N_2_ stream. Water (1.5 mL) was added, and the water phase was washed with Et_2_O (2 × 1.5 mL). The water phase was then evaporated *in vacuo* before being taken up in MilliQ water and purified via RP-HPLC. MALDI: *m*/*z* [M+H]^+^ calcd for [C_150_H_186_N_72_O_38_+H^+^]: 3605.4942; Found: 3606.2969. 

The PNAzymes were purified on an Ascentis Express Supelco Peptide ES-C18 column (2.7 µm, 150 × 4.6 mm) with a linear gradient elution of 0% to 40% buffer B over 30 min at 60 °C, using a flow rate of 1.0 mL/min and UV detection at 260 nm. Solvent system used: solvent A: 0.1% TFA in water and solvent B: 50% MeCN: water containing 0.1% TFA. Collected products were evaporated to dryness and lyophilised from water (×3). The final products were analysed via MALDI-MS in positive ion mode using a sinapic acid matrix (10 mg/mL in 0.1% TFA/milliQ and MeCN (2:1, *v*/*v*).

### 3.3. The Reaction of HPNPP

Stock solutions of HPNPP (5.0 mM), HEPES Buffer (0.1 M), Zn(II)Cl_2_ or Zn(NO)_2_ (50 mM) and the respective ligand (50 mM, DMSO) were prepared. Reaction mixtures were prepared directly in the cuvette by adding the ligand, Zn(II), buffer and water, giving a total volume of 2 mL and final concentrations of [HPNPP] = 0.05 mM and [HEPES] = 50 mM. The cuvettes were placed in the UV cell block to equilibrate. HPNPP was added as the last component and the complete reaction was followed at 400 nm. For the faster reactions, the integrated first order rate equation was fitted to the increase in absorbance with time to obtain the observed first rate constant (*k*_obs_) for each reaction. For slower reactions, initial rate experiments followed the same procedure but with use of HPNPP stock—solution of 50 mM and final concentrations of 5.0 mM 2–5% of the reaction. Data were fitted to straight lines and divided by the extinction coefficient ε = 9575 at a pH of 7.0.

### 3.4. The Reaction of PNAzymes

RNA cleavage reactions were carried out in sealed tubes immersed in a thermostated water bath (37 °C). Experiments were performed at a pH of 7.0 in a HEPES buffer (10 mM HEPES, 0.1 M NaCl). RNA targets (4 µM final concentration 1 eq) were equilibrated in appropriate amounts of water and HEPES buffer over a 15 min period at 37 °C prior to the addition of Zn(II) solution (100 µM final concentration) and 1.3 eq. of the PNAzyme. The reaction mixtures were then allowed to incubate at 37 °C. Aliquots (40 µL) were withdrawn at 3 h, 6 h, 24 h, 48 h, 72 h and 96 h time points and immediately quenched with 70 µL of 1.0 mM EDTA in 30% MeCN/milliQ. The samples were analysed with anion exchange HPLC (IE-HPLC) using a Dionex NucleoPac PA-100 (Thermo Scientific, Waltham, MA, USA, 4 × 250 mm) column with a linear gradient elution of 0–45% buffer B over 30 min at 60 °C. A flow rate of 1.5 mL/min was used and UV detection was carried out at 260 nm. The following solvent system was used: (A) 20 mM NaOAc in 30% aq. MeCN and (B) 20 mM NaOAc, 0.4 M LiClO4 in 30% aq. MeCN. Cleavage of RNA substrates was measured via quantification of the remaining RNA and the sum of the formed fragments detected in the IE-HPLC analysis.

### 3.5. Titrations 

For the titration experiments, the following standard conditions were used for all experiments. All solutions used were freshly made from chemicals of analytical grade and using miliQ water. For titration of the ligand itself, the ligand (1 eq; 0.02 mmol) was taken up in NaNO_3_ (0.10 M) (10 000 µL). HCl (1 M) (40 µL; 2 eq) was added and the solution was placed in a water bath at 25 °C. The solution was titrated with NaOH (0.2 M) (10 µL aliquots; 1/10 eq.). The stirring was turned on after each addition but turned off before each measurement to allow the pH meter to stabilize. For titration of the ligand in presence of a metal ion, the ligand (1 eq; 0.02 mmol) was taken up in NaNO_3_ (0.10 M) + Zn(NO_3_)_2_ (2.0 mM; 1 eq) (10 000 µL). HCl (1 M) (40 µL; 2 eq) was added and the solution was placed in a water bath at 25 °C. The solution was titrated with NaOH (0.2 M) (10 µL per addition; 1/10 eq.). The stirring was turned on after each addition and the pH was allowed to stabilize before each measurement without stirring.

## 4. Conclusions

*N*,*N*-bis(2-picolyl)amine derivatives suitable for conjugation to PNA were synthesized with an improved synthesis of the 6-amino analogues. It was found that the data obtained for the Zn(II) systems could be translated to the PNA conjugate systems and a similar trend based on the ligand substitution was found. The 2-amino group clearly increased the cleavage rate in all systems tested for different bulge sizes and metals. The difference in relative rates obtained for the cleavage of HPNPP were in general bigger compared to the relative rates obtained from the PNAzymes experiments. This study shows that kinetic data obtained for different ligands based on the model substrate HPNPP in solution can, to some extent, be translated into the reactivity of conjugated systems like artificial nucleases and PNAzymes. The observed rate ratios of the different PNAzymes represent the minimum differences in activity. It is likely that Zn(II) ions in solution contribute to the observed reactions, which would mean that the differences are greater. Thus, the substituents do have a similar effect on the rate of cleavage for an RNA target and the much simpler reaction with HPNPP. The observed rates of reaction for these complexes are far too slow to use for therapeutic reactions, but the perturbations of the reactivity by the variations in the ligand structure provide a useful insight into how more active complexes may be enhanced to create genuinely viable PNAzymes. The synthetic work described here also provides a route which may facilitate introducing the required moieties.

## Figures and Tables

**Table 1:** Bond lengths (Å) to the Zn ion of the *N*,*N*-bis(2-picolyl) amine ligands **12**–**14** complexed with ZnCl_2_.

	12.ZnCl_2_	13.ZnCl_2_	14.ZnCl_2_
Cl1	2.2614(4)	2.2898(7)	2.2827(8)
Cl2	2.2817(4)	2.2539(8)	2.2849(9)
N1	2.1697(13)	2.2421(13)	2.199(3)
N2	2.1741(14)	2.1468(13)	2.139(3)
N3	2.2005(13)	2.1468(13)	2.193(3)

**Table 2:** p*K*_a_ and formation constants for the ligand complexes **12**–**14**.

Ligand	p*K*_a_ (LH_2_^2+^)	p*K*_a_ (LH^+^)	Species	log*K*_f_	p*K*_a_^1^	p*K*_a_^2^
**12**	3.28 ± 0.04	5.89 ± 0.04	**12.**Zn	6.31 ± 0.02	8.50 ± 0.03	10.8 ± 0.03
**13**	4.15 ± 0.05	6.54 ± 0.06	**13.**Zn	4.65 ± 0.07	7.3 ± 0.1	9.1 ± 0.1
**14**	5.54 ± 0.03	7.59 ± 0.04	**14.**Zn	6.07 ± 0.03	8.15 ± 0.05	10.8 ± 0.06

**Table 3:** Rate constants for the cleavage of HPNPP catalysed by zinc chloride and the zinc complexes of **12**–**14**.

Species	2nd Order Rate Constant M^−1^ s^−1^	*k*_rel_ (Zn)	*k*_rel_ (12.Zn)
Zn	4.6 ± 0.1 × 10^−3^	1	3.8
**12.**Zn	1.2 ± 0.1 × 10^−3^	0.26	1
**13.**Zn	3.90 ± 0.09 × 10^−3^	0.85	3.3
**14.**Zn	1.18 ± 0.03 × 10^−1^	26	98

**Table 4:** Rate constants for the cleavage of RNA catalysed by 4 µM PNAzyme in the presence of 100 µM ZnCl_2_ at pH 7 and 37 °C.

		*k*_obs_ (s^−1^)			*k*_rel_ (PNAzyme 1)	
Bulge	PNAzyme 1	PNAzyme 2	PNAzyme 3	PNAzyme 1	PNAzyme 2	PNAzyme 3
–AAAA–	3.73 × 10^−7^	5.86 × 10^−7^	5.52 × 10^−6^	1	1.6	15
–AAA–	1.42 × 10^−7^	3.20 × 10^−7^	3.80 × 10^−6^	1	2.3	27

## Data Availability

Data are contained within the article and Supplementary Materials.

## Supplementary Materials

The following supporting information can be downloaded at: https://www.mdpi.com/article/10.3390/molecules29092123/s1. Synthesis; Metal complex formation; Synthesis of PNA conjugates; Titration experiments; Speciation Curves; The reaction of HPNPP; The reaction of PNAzymes.
