# Supplementary material for: Zinc N,N-bis(2-picolyl)amine Chelates Show Substitution-Dependent Cleavage of Phosphodiesters in Models as Well as of PNAzyme-RNA Bulges
Source: Molecules. 2024 May 3;29(9):2123. doi: 10.3390/molecules29092123 (PMC11085541; doi:10.3390/molecules29092123)
Supplement: Supplementary file 1 [file molecules-29-02123-s001.zip › molecules-2919454-supplementary.pdf]

Zinc *N*,*N*-bis(2-picolyl)amine Chelates Show Substitution-Dependent Cleavage of Phosphodiesters in Models as Well as of PNAzyme-RNA Bulges

Supporting Information

Contents

Synthesis 4
(6-Methylpyridin-2-yl)methyl methanesulfonate 4
2-(Iodomethyl)-6-methylpyridine 4
Diethyl pyridine-2,6-dicarboxylate **3** 4
6-(Ethoxycarbonyl)picolinic acid **4** 5
Ethyl 6-((tert-butoxycarbonyl)amino)picolinate **5** 5
Tert-butyl (6-(hydroxymethyl)pyridin-2-yl)carbamate **1** 5
(6-((Tert-butoxycarbonyl)amino)pyridin-2-yl)methyl methanesulfonate **6** 6
Tert-butyl (6-(iodomethyl)pyridin-2-yl)carbamate **7** 6
Methyl 4-(aminomethyl)benzoate hydrochloride **8** 7
Methyl 4-((bis(pyridin-2-ylmethyl)amino)methyl)benzoate 7
Methyl 4-((bis((6-methylpyridin-2-yl)methyl)amino)methyl)benzoate 7
Methyl 4-((bis((6-((tert-butoxycarbonyl)amino)pyridin-2-yl)methyl)amino)methyl)-benzoate 8
4-((Bis(pyridin-2-ylmethyl)amino)methyl)benzoic acid **9** 8
4-((Bis((6-methylpyridin-2-yl)methyl)amino)methyl)benzoic acid **10** 9
4-((Bis((6-((tert-butoxycarbonyl)amino)pyridin-2-yl)methyl)amino)methyl)benzoic acid **11** .. 9
4-((Bis(pyridin-2-ylmethyl)amino)methyl)-N-methylbenzamide **12** 10
4-((Bis((6-methylpyridin-2-yl)methyl)amino)methyl)-N-methylbenzamide **13** 10
Di-tert-butyl ((((4-(methylcarbamoyl)benzyl)azanediyl)bis(methylene))bis(pyridine-6,2-diyl))dicarbamate 11
4-((Bis((6-aminopyridin-2-yl)methyl)amino)methyl)-N-methylbenzamide **14** 11
(6-Methylpyridin-2-yl)methyl methanesulfonate 12
2-(Iodomethyl)-6-methylpyridine 13
Diethyl pyridine-2,6-dicarboxylate **3** 14
6-(Ethoxycarbonyl)picolinic acid **4** 15
Ethyl 6-((tert-butoxycarbonyl)amino)picolinate **5** 16
Tert-butyl (6-(hydroxymethyl)pyridin-2-yl)carbamate **1** 17
(6-((Tert-butoxycarbonyl)amino)pyridin-2-yl)methyl methanesulfonate **6** 18
Tert-butyl (6-(iodomethyl)pyridin-2-yl)carbamate **7** 19
Methyl 4-(aminomethyl)benzoate hydrochloride **8** 20
Methyl 4-((bis(pyridin-2-ylmethyl)amino)methyl)benzoate 21
Methyl 4-((bis((6-methylpyridin-2-yl)methyl)amino)methyl)benzoate 22
Methyl 4-((bis((6-((tert-butoxycarbonyl)amino)pyridin-2-yl)methyl)amino)methyl)-benzoate 23
4-((bis(pyridin-2-ylmethyl)amino)methyl)benzoic acid **9** 24
4-((bis((6-methylpyridin-2-yl)methyl)amino)methyl)benzoic acid **10** 25
4-((bis(pyridin-2-ylmethyl)amino)methyl)-N-methylbenzamide **12** 27

4-((bis((6-methylpyridin-2-yl)methyl)amino)methyl)-N-methylbenzamide **13** 28

di-tert-butyl ((((4-(methylcarbamoyl)benzyl)azanediyl)bis(methylene))bis(pyridine-6,2-diyl))dicarbamate 29

4-((bis((6-aminopyridin-2-yl)methyl)amino)methyl)-N-methylbenzamide **14** 30

Metal complex formation 31

4-((bis(pyridin-2-ylmethyl)amino)methyl)-N-methylbenzamide **12** $\cdot ZnCl_2$ Complex 31

4-((bis((6-methylpyridin-2-yl)methyl)amino)methyl)-N-methylbenzamide **13** $\cdot ZnCl_2$ Complex 31

4-((bis((6-aminopyridin-2-yl)methyl)amino)methyl)-N-methylbenzamide **14** $\cdot ZnCl_2$ Complex 31

Synthesis of PNA conjugates 31

Titration experiments 34

Speciation Curves 35

The reaction of HPNPP 36

The reaction of PNAzymes 36

Synthesis

(6-Methylpyridin-2-yl)methyl methanesulfonate

(6-methylpyridin-2-yl)methanol (3.00 g; 24.4 mmol) was dissolved in THF (40 ml) and cooled on an ice bath. Et_3N (2.96 g; 29.3 mmol) was added followed by methanesulfonyl chloride (3.21 g; 28.0 mmol). The reaction was left to stir overnight. The reaction mixture was poured into aqueous NH_4Cl (sat) (150 ml) and extracted with diethyl ether (3x50 ml). The combined ether phase was dried over $MgSO_4$, filtered and evaporated *in vacuo* giving an orange oil. Yield: 4.64 g (95%)

^{1}H NMR (400 MHz, $CDCl_3$) δ 7.66 (t, J = 7.7 Hz, 1H), 7.29 (d, J = 7.7 Hz, 1H), 7.17 (d, J = 7.7 Hz, 1H), 5.31 (s, 2H), 3.10 (s, 3H), 2.57 (s, 3H). ^{13}C NMR (101 MHz, $CDCl_3$) δ 158.6, 152.8, 137.5, 123.5, 119.6, 71.9, 38.2, 24.4. HRMS (ESI): m/z $[M+H]^+$ calcd. for [$C_8H_{11}NO_3S$ +H^+]: 202.0532; found: 202.0532 100 FT-IR (Vmax/cm^{-1}): 3383(w), 3023(w), 2932(w), 1596(m), 1577(w), 1460(m), 1347(s), 1334(s), 1171(s), 1028(m), 966(s), 945(s)

2-(Iodomethyl)-6-methylpyridine

(6-methylpyridin-2-yl)methyl methanesulfonate (2.00 g; 9.94 mmol) was dissolved in THF (30 ml) and LiI (2.86 g; 21.4 mmol) was added. The reaction was heated to 50°C for 90 min. The THF solution was poured into aqueous NH_4Cl (sat.) (100 ml) and extracted with chloroform (3x50 ml). The combined organic phase was washed with $Na_2S_2O_3$ (10% W/W solution) (50 ml) and dried over $MgSO_4$, filtered and evaporated *in vacuo*, giving an orange oil. Yield: 2.24 g (97%)

^{1}H NMR (400 MHz, $CDCl_3$) δ 7.50 (t, J = 7.7 Hz, 1H), 7.19 (d, J = 7.7 Hz, 1H), 6.99 (d, J = 7.7 Hz, 1H), 4.46 (s, 2H), 2.52 (s, 3H). ^{13}C NMR (101 MHz, $CDCl_3$) δ 158.4, 157.6, 137.3, 122.3, 120.0, 24.6, 6.8. HRMS (ESI): m/z $[M+H]^+$ calcd. for [C_7H_8IN +H^+]: 233.9774; found: 233.9771 FT-IR (Vmax/cm^{-1}): 3230(w), 2966(w), 2924(w), 2870(w), 1591(s), 1575(s), 1452(s), 1159(s), 1035(m), 989(m), 790(m)

Diethyl pyridine-2,6-dicarboxylate **3**

Pyridine-2,6-dicarboxylic acid (30.00 g; 179.5 mmol) was taken up in EtOH (200 ml). A catalytic amount of conc. H_2SO_4 (95%) (2 ml) was added and the mixture was refluxed for 24 h. The ethanol phase was concentrated *in vacuo* and $NaHCO_3$ (sat.) (200 ml) was added. The aqueous phase was extracted with $CHCl_3$ (2x200 ml) and the combined organic phase was dried over $MgSO_4$, filtered and evaporated *in vacuo* giving a colourless oil which crystallised giving a colourless crystalline solid. Yield: 30.83 g (77%), Mp: 40-42 °C

^{1}H NMR (400 MHz, $CDCl_3$) δ 8.23 (d, J = 7.8 Hz, 2H), 7.96 (t, J = 7.8 Hz, 1H), 4.43 (q, J = 7.1 Hz, 4H), 1.40 (t, J = 7.1 Hz, 6H). ^{13}C NMR (101 MHz, $CDCl_3$) δ 164.6, 148.6, 138.3, 127.8, 62.3, 14.2. HRMS (ESI): m/z $[M+H]^+$ calcd. for [$C_{11}H_{13}NO_4$ +H^+]: 224.0917; found: 224.0921 FT-IR (Vmax/cm^{-1}): 3100(w), 3062(w), 2985(m), 2944(w), 2907(w), 1742(vs), 1238(vs)

6-(Ethoxycarbonyl)picolinic acid **4**

Diethyl pyridine-2,6-dicarboxylate **3** (30.22 g; 135.4 mmol) was dissolved in a mixture of EtOH (50ml) and dioxane (400 ml). NaOH (5.42 g; 135.5 mmol; 1 eq) was dissolved in EtOH (150 ml) and added to the first solution. The combined mixture was heated to 100 °C for 5 h. The solvent was removed *in vacuo* and the residue was taken up in water (400 ml). The aqueous phase was washed with $CHCl_3$ (3x100ml) before the pH was lowered to pH 1 with conc. HCl. The aqueous phase was then extracted with $CHCl_3$ (4x150 ml) and the combined organic phase was dried over $MgSO_4$, filtered and evaporated *in vacuo* giving a white solid. Yield: 15.07 g (57%). Mp: 120-122 °C

^{1}H NMR (400 MHz, $CDCl_3$) δ 10.25 (s, 1H), 8.39 (dd, J = 7.7, 1.1 Hz, 1H), 8.35 (dd, J = 7.8, 1.1 Hz, 1H), 8.11 (t, J = 7.8 Hz, 1H), 4.48 (q, J = 7.1 Hz, 2H), 1.44 (t, J = 7.1 Hz, 3H). ^{13}C NMR (101 MHz, $CDCl_3$) δ 163.8, 147.2, 146.5, 139.7, 128.9, 126.8, 62.6, 14.3. HRMS (ESI): m/z $[M+H]^+$ calcd. for $[C_9H_9NO_4 + H^+]$: 196.0604; found: 196.0603 FT-IR (Vmax/cm^{-1}):3064(w), 2987(w), 2816(w), 2675(w), 2548(w), 1737(vs), 1698(vs), 1575(s), 1245(vs), 1151(s), 1023(m), 759(s), 709(s), 694(s), 650(s)

Ethyl 6-((tert-butoxycarbonyl)amino)picolinate **5**

6-(Ethoxycarbonyl)picolinic acid **4** (7.00 g; 35.9 mmol) was dissolved in toluene (150 ml) and Et_3N (9.80 ml; 70.7 mmol) was added. Diphenylphosphoryl azide (11.42 g; 41.5 mmol) was added and the reaction was stirred at RT for 10 min before t-BuOH (15 ml; 157 mmol) was added. The reaction mixture was heated to 100 °C for 24h. The reaction was allowed to cool down to RT and EtOAc (200 ml) was added. The organic phase was washed with $NaHCO_3$ (sat.) (2x100 ml) and NaCl (sat.) (100 ml). The organic phase was dried over $MgSO_4$, filtered and evaporated *in vacuo* giving a dark brown oil which was purified by column chromatography (Eluent: EtOAc/Hex, 20:80) to give a clear colourless oil. Yield 6.35 g, (66%).

^{1}H NMR (400 MHz, $CDCl_3$) δ 8.13 (dd, J = 7.2, 1.9 Hz, 1H), 7.82 – 7.74 (m, 2H), 7.64 (s, 1H), 4.43 (q, J = 7.1 Hz, 2H), 1.49 (s, 9H), 1.40 (t, J = 7.1 Hz, 3H). ^{13}C NMR (101 MHz, $CDCl_3$) δ 164.9, 152.4, 152.0, 139.2, 120.0, 116.1, 81.4, 62.0, 28.3, 14.4. HRMS (ESI): m/z $[M+H]^+$ calcd. for $[C_{13}H_{18}N_2O_4 + H^+]$: 267.1339; found: 267.1351

Tert-butyl (6-(hydroxymethyl)pyridin-2-yl)carbamate **1**

Ethyl 6-((tert-butoxycarbonyl)amino)picolinate **5** (9.63 g; 36.2 mmol) was taken up in EtOH (150 ml) and $CaCl_2$ (8.03 g; 72.4 mmol) was added. The suspension was cooled to 0°C and $NaBH_4$ (6.84 g; 181 mmol) was added. The mixture was stirred for 5 h and allowed to rise to RT. The reaction mixture was concentrated *in vacuo* and water (500 ml) was added. The aqueous phase was extracted with $CHCl_3$ (4x100 ml). The organic layer was dried over $MgSO_4$, filtered and evaporated *in vacuo* giving a yellow oil. The residue was purified by column chromatography (Eluent: EtOAc/Hex, 1:2) giving a colourless oil. Yield: 6.87g (78%)

^{1}H NMR (400 MHz, $CDCl_3$) δ 7.82 (d, J = 8.3 Hz, 1H), 7.65 (dd, J = 8.1, 7.6 Hz, 1H), 7.49 (s, 1H), 6.90 (d, J = 7.4 Hz, 1H), 4.64 (s, 2H), 1.52 (s, 9H) ^{13}C NMR (101 MHz, $CDCl_3$) δ 157.5, 152.3, 151.2, 139.2, 115.1, 110.8, 81.4, 63.9, 28.4. HRMS (ESI): m/z $[M+H]^+$ calcd. for $[C_{11}H_{16}N_2O_3 +H^+]$: 225.1234; found: 225.1231 FT-IR (Vmax/cm^{-1}):3222(w), 2980(w), 2934(w), 1727(s), 1582(m), 1522(s), 1457(s), 1367(m), 1225(s), 1149(vs), 1083(s), 878(m), 792(m)

(6-((Tert-butoxycarbonyl)amino)pyridin-2-yl)methyl methanesulfonate **6**

Tert-butyl (6-(hydroxymethyl)pyridin-2-yl)carbamate **1** (2.00 g; 8.96 mmol) was dissolved in EtOAc (50 ml) and Et_3N (2.27 g; 22.4 mmol) was added. The mixture was cooled on an ice bath and MsCl (2.57 g; 22.4 mmol) was added. The reaction was stirred at RT for 2 h. The solution was poured out in $NaHCO_3$ (sat.) (100 ml) and the organic phase was separated and washed with additional $NaHCO_3$ (sat.)(100 ml), water (2x100 ml) and NaCl (sat.) (50 ml). The organic phase was dried over $MgSO_4$, filtered and evaporated *in vacuo* giving a colourless oil. Yield: 2.60 g (96%)

^{1}H NMR (400 MHz, $CDCl_3$) δ 7.92 (d, J = 8.4 Hz, 1H), 7.70 (t, J = 7.9 Hz, 1H), 7.58 (s, 1H), 7.08 (d, J = 7.4 Hz, 1H), 5.17 (s, 2H), 3.03 (s, 3H), 1.50 (s, 9H). ^{13}C NMR (101 MHz, $CDCl_3$) δ 152.3, 151.9, 151.7, 139.4, 117.1, 112.4, 81.4, 71.2, 38.2, 28.3 HRMS (ESI): m/z $[M+H]^+$ calcd. for $[C_{12}H_{18}N_2O_5S+H^+]$: 303.1009; found: 303.1014; m/z $[M+Na]^+$ calcd. for $[C_{12}H_{18}N_2O_5S+Na^+]$: 325.0829; found: 325.0833 FT-IR (Vmax/cm^{-1}): 3222(w), 2980(w), 2938(w), 1727(s), 1582(m), 1522(m), 1463(m), 1354(s), 1223(m), 1168(s), 1152(s), 1083(m), 955(s), 800(m)

Tert-butyl (6-(iodomethyl)pyridin-2-yl)carbamate **7**

(6-((Tert-butoxycarbonyl)amino)pyridin-2-yl)methyl methanesulfonate **6** (2.48 g; 8.20 mmol) was dissolved in THF(75 ml) and NaI (2.46 g; 16.4 mmol) was added. The reaction mixture was heated to 50 °C and stirred for 90 min. The reaction mixture was allowed to cool to RT and poured into aqueous NH_4Cl (sat.) (50 ml) and extracted with $CHCl_3$ (2x40 ml). The combined organic phase was washed with $Na_2S_2O_3$ (aq) (10w/w%) (25 ml) and the combined organic phase was dried over $MgSO_4$, filtered and evaporated *in vacuo* to give a yellow oil. Yield: 2.28 g (83%)

^{1}H NMR (400 MHz, $CDCl_3$) δ 7.79 (d, J = 8.4 Hz, 1H), 7.57 (t, J = 7.9 Hz, 1H), 7.51 (s, 1H), 7.00 (d, J = 7.4 Hz, 1H), 4.36 (s, 2H), 1.49 (s, 9H). ^{13}C NMR (101 MHz, $CDCl_3$) δ 156.4, 152.3, 151.7, 139.2, 117.3, 111.3, 81.1, 28.3, 6.1. HRMS (ESI): m/z $[M+H]^+$ calcd. for $[C_{11}H_{15}IN_2O_2 +H^+]$: 335.0251; found: 335.0252; m/z $[M+Na]^+$ calcd. for $[C_{11}H_{15}IN_2O_2 +Na^+]$: 357.0070; found: 357.0076 FT-IR (Vmax/cm^{-1}): 3226(w), 2980(w), 2934(w), 1727(s), 1580(s), 1517(s), 1452(s), 1402(m), 1365(m), 1279(m), 1223(m), 1149(s), 986(m), 886(m), 796(m)

Methyl 4-(aminomethyl)benzoate hydrochloride **8**

4-(Aminomethyl)benzoic acid (1.20 g; 7.93 mmol) was suspended in methanol (90 ml) and thionyl chloride (9.0 ml; 124 mmol) was added dropwise. The solution was allowed to cool to RT and the flask was equipped with a stopper and left to stir overnight. The methanolic solution was evaporated *in vacuo* and the white salt was triturated in diethyl ether. The ether phase was decanted off and the white salt was dried *in vacuo*. Yield: 1.53 g (96%) mp: 234-236 °C

^1H NMR (400 MHz, DMSO) δ 8.72 (s, 3H), 7.97 (d, J = 8.3 Hz, 2H), 7.66 (d, J = 8.4 Hz, 2H), 4.09 (s, 2H), 3.86 (s, 3H). ^{13}C NMR (101 MHz, DMSO) δ 165.9, 139.5, 129.5, 129.2, 129.2, 52.3, 41.7. HRMS (ESI): m/z $[M+H]^+$ calcd. for $[C_9H_{11}NO_2 + H^+]$: 166.0863; found: 166.0869 FT-IR (Vmax/cm^{-1}): 3005(s,Br), 2961(s,Br), 2881(s,Br), 1720(vs), 1597(w), 1478(m), 1466(m), 1436(m), 1381(w), 1315(w), 1281(vs), 1190(m), 1117(s), 1110(s), 1077(m), 963(m), 854(m), 863(m), 854(m), 835(m), 763(s), 703(s), 625(m), 532(m)

Methyl 4-((bis(pyridin-2-ylmethyl)amino)methyl)benzoate

2-(Bromomethyl)pyridine hydrobromide (0.56 g; 2.2 mmol) and methyl 4-(aminomethyl)benzoate hydrochloride (0.20 g; 0.99 mmol) was added to a flame dried flask and the flask was equipped with a rubber septum. The atmosphere was changed to argon and DMF (dry) (10 ml) was added via a syringe. DIPEA (0.71 g; 5.5 mmol) was added and the flask was equipped with an argon balloon. The stirred reaction mixture was heated to 70 °C overnight. The reaction mixture was poured into water (75 ml) and extracted with diethyl ether (3x30 ml). The combined ether phase was dried over $MgSO_4$, filtered and evaporated *in vacuo*. The residue was purified by column chromatography (Eluent: EtOAc/EtOH, 9:1; Rf: tailing) giving a yellow oil. Yield: 0.24 g (71%)

^1H NMR (400 MHz, $CDCl_3$) δ 8.50 (ddd, J = 5.0, 1.7, 1.0 Hz, 2H), 7.97 (d, J = 8.3 Hz, 2H), 7.65 (td, J = 7.7, 1.7 Hz, 2H), 7.54 (d, J = 7.8 Hz, 2H), 7.48 (d, J = 8.2 Hz, 2H), 7.13 (ddd, J = 7.3, 4.9, 0.9 Hz, 2H), 3.87 (s, 3H), 3.79 (s, 4H), 3.72 (s, 2H). ^{13}C NMR (101 MHz, $CDCl_3$) δ 167.1, 159.4, 149.1, 144.7, 136.6, 129.7, 129.0, 128.7, 122.9, 122.2, 60.2, 58.3, 52.1. HRMS (ESI): m/z $[M+H]^+$ calcd for $[C_{21}H_{21}N_3O_2 + H^+]$: 348.1707; found: 348.1703.

Methyl 4-((bis((6-methylpyridin-2-yl)methyl)amino)methyl)benzoate

2-(Iodomethyl)-6-methylpyridine (1.00 g; 4.29 mmol) and the methyl 4-(aminomethyl)benzoate hydrochloride (0.35 g; 1.74 mmol) was added to a flame dried flask and the flask was equipped with a rubber septum. The atmosphere was changed to argon and DMF (10 ml) was added via a syringe. DIPEA (1.12 g; 8.67 mmol) was added and the flask was equipped with an argon balloon. The stirred reaction mixture was heated to 70 °C overnight. The reaction mixture was poured into water (75 ml) and extracted with diethyl ether (3x30 ml). The combined ether phase was washed with NaCl (sat.) (2x20 ml), dried over $MgSO_4$, filtered and evaporated *in vacuo*. The

residue was purified by column chromatography (Eluent: EtOAc/EtOH, 9:1; Rf: tailing) to give a yellow oil. Yield: 0.54 g (83%)

^{1}H NMR (400 MHz, $CDCl_3$) δ 7.96 (d, J = 8.1 Hz, 2H), 7.54 (t, J = 7.6 Hz, 2H), 7.48 (d, J = 8.1 Hz, 2H), 7.40 (d, J = 7.7 Hz, 2H), 6.99 (d, J = 7.6 Hz, 2H), 3.88 (s, 3H), 3.77 (s, 4H), 3.71 (s, 2H), 2.50 (s, 6H). ^{13}C NMR (101 MHz, $CDCl_3$) δ 167.1, 159.0, 157.7, 145.0, 136.8, 129.7, 128.9, 128.7, 121.6, 119.5, 60.3, 58.3, 52.1, 24.5. HRMS (ESI): m/z $[M+H]^+$ calcd for $[C_{23}H_{25}N_3O_2 + H^+]$: 376.2020; found: 376.2003. FT-IR (Vmax/cm^{-1}): 3062(w), 2996(w), 2951(w), 2923(w), 2829(w), 1718(vs), 1579(m), 1577(m), 1456(m), 1435(m), 1275(vs), 1155(s), 786(m), 756(m), 732(m)

Methyl 4-((bis((6-((tert-butoxycarbonyl)amino)pyridin-2-yl)methyl)amino)methyl)-benzoate

Tert-butyl (6-(iodomethyl)pyridin-2-yl)carbamate (0.38 g; 1.14 mmol) and the methyl 4- (aminomethyl)benzoate hydrochloride (0.10 g; 0.50 mmol) was added to a flame dried flask and the flask was equipped with a rubber septum. The atmosphere was changed to argon and DMF (4 ml) was added via a syringe. DIPEA (0.33 g; 2.6 mmol) was added and the flask was equipped with an argon balloon. The stirred reaction mixture was heated to 70 °C overnight. The reaction mixture was poured into water (25 ml) and extracted with diethyl ether (3x15 ml). The combined ether phase was washed with NaCl (sat.) (2x10 ml), dried over $MgSO_4$, filtered and evaporated *in vacuo*. The residue was purified by column chromatography (Eluent: Hex/EtOAc, 80:20; Rf:0.20, tailing) to give a colourless oil which crystallised to give a white solid. Yield: 0.22 g (76%) mp: 134-136 °C

^{1}H NMR (400 MHz, $CDCl_3$) δ 7.97 (d, J = 8.2 Hz, 2H), 7.77 (d, J = 8.2 Hz, 2H), 7.63 (t, J = 7.9 Hz, 2H), 7.49 – 7.42 (m, 4H), 7.16 (d, J = 7.4 Hz, 2H), 3.89 (s, 3H), 3.69 (s, 2H), 3.65 (s, 4H), 1.48 (s, 18H). ^{13}C NMR (101 MHz, $CDCl_3$) δ 167.1, 157.7, 152.5, 151.3, 144.6, 138.7, 129.8, 129.1, 128.8, 117.6, 110.5, 81.0, 59.5, 58.0, 52.2, 28.4. HRMS (ESI): m/z $[M+H]^+$ calcd. for $[C_{31}H_{39}N_5O_6 + H^+]$: 578.2973; found: 578.2979; m/z $[M+Na]^+$ calcd. for $[C_{31}H_{39}N_5O_6 + Na^+]$: 600.2793; found: 600.2795. FT-IR (Vmax/cm^{-1}): 3198(w), 2978(w), 2928(w), 2824(w), 1724(s), 1579(m), 1525(m), 1456(s), 1276(s), 1228(s), 1149(s), 1068(m), 991(m), 800(m)

4-((Bis(pyridin-2-ylmethyl)amino)methyl)benzoic acid **9**

Methyl 4-((bis(pyridin-2-ylmethyl)amino)methyl)benzoate (0.91 g; 2.6 mmol) was dissolved in a minimum volume of methanol. NaOH (2 M) (10 ml) was added, and the mixture was stirred at RT for 5 h. HCl (1 M) (20 ml) was added and the solution was evaporated *in vacuo*. DCM (50 ml) was added, followed by Na_2SO_4 and the suspension was stirred for 5 min. The suspension was filtered and the filtrate was concentrated *in vacuo*. The residue was taken up in hot acetonitrile (15 ml) and allowed to cool to RT before being left at 4 °C overnight. The solvent was pipetted off and the crystals was washed with additional acetonitrile to give a white solid. Yield: 0.56 g (64%) mp.: 168- 170 °C

^{1}H NMR (400 MHz, DMSO) δ 12.90 (s, 1H), 8.49 (ddd, J = 4.8, 1.7, 0.9 Hz, 2H), 7.90 (d, J = 8.2 Hz, 2H), 7.78 (td, J = 7.7, 1.8 Hz, 2H), 7.57 (d, J = 7.8 Hz, 2H), 7.53 (d, J = 8.2 Hz, 2H), 7.25 (ddd, J = 7.4, 4.7, 0.8 Hz, 2H), 3.71 (s, 4H), 3.70 (s, 2H). ^{13}C NMR (101 MHz, DMSO) δ 167.3, 158.9, 148.9, 144.2, 136.7, 129.5, 129.4, 128.7, 122.6, 122.2, 59.2, 57.2. HRMS (ESI): m/z $[M+H]^+$ calcd. for $[C_{20}H_{19}N_3O_2 +H^+]$: 334.1550; found: 334.1551. FT-IR (Vmax/cm-1): 3064(w), 2881(w), 2814(w), 2463(b), 1897(b), 1692(m), 1599(m), 1571(m), 1433(m), 1273(s), 1247(s) 1013(m), 763(s), 753(s)

4-((Bis((6-methylpyridin-2-yl)methyl)amino)methyl)benzoic acid **10**

Methyl 4-((bis((6-methylpyridin-2-yl)methyl)amino)methyl)benzoate (1.12 g; 3.0 mmol) was dissolved in a minimum volume of methanol. NaOH (2 M) (10 ml) was added, and the mixture was stirred at RT for 5 h. HCl (1 M) (20 ml) was added and the solution was evaporated *in vacuo*. DCM (50 ml) was added, followed by Na_2SO_4 and the suspension was stirred for 5 min. The suspension was filtered and the filtrate was concentrated *in vacuo*. The residue was taken up in hot acetonitrile (15 ml) and allowed to cool to RT before being left at 4°C overnight. The solvent was pipetted off and the crystals were washed with additional acetonitrile to give a white solid. Yield: 0.41 g (38%) mp.: 146-148 °C

^{1}H NMR (400 MHz, DMSO) δ 12.90 (s, 1H), 7.91 (d, J = 8.2 Hz, 2H), 7.64 (t, J = 7.7 Hz, 2H), 7.53 (d, J = 8.2 Hz, 2H), 7.38 (d, J = 7.7 Hz, 2H), 7.08 (d, J = 7.6 Hz, 2H), 3.66 (s, 2H), 3.65 (s, 4H), 2.41 (s, 6H). ^{13}C NMR (101 MHz, DMSO) δ 167.3, 158.3, 157.0, 144.3, 136.9, 129.5, 129.4, 128.6, 121.4, 119.3, 59.3, 57.2, 24.0. HRMS (ESI): m/z $[M+H]^+$ calcd. for $[C_{22}H_{23}N_3O_2 +H^+]$: 362.1863; found: 362.1860. FT-IR (Vmax/cm^{-1}): 3061(w), 2920(w), 2834(w), 2441(w),1692(s), 1601(m), 1591(m), 1578(s), 1458(s), 1275(vs), 1244(vs), 1122(m), 1013(m), 973(m), 795(vs), 778(vs), 756(vs)

4-((Bis((6-((tert-butoxycarbonyl)amino)pyridin-2-yl)methyl)amino)methyl)benzoic acid **11**

Methyl 4-((bis((6-((tert-butoxycarbonyl)amino)pyridin-2 yl)methyl)amino)methyl)-benzoate (0.20 g; 0.35 mmol) was dissolved in EtOH (10 ml) and NaOH(s) (0.40 g; 10 mmol) was added and left to dissolve and react on sonication for 1.5h. HCl (1M) (10ml) was added and the clear mixture was concentrated *in vacuo*. The residue was taken up in DCM (25 ml), and $MgSO_4$ was added. After 5 min of stirring, the suspension was filtered and the filtrate was concentrated *in vacuo*. The residue was recrystallized from hot ethyl acetate to give a white crystalline solid. Yield: 0.13 g (67%) Mp: 128-130°C

^{1}H NMR (400 MHz, MeOD) δ 8.01 (d, J = 8.2 Hz, 2H), 7.77 – 7.71 (m, 4H), 7.66 (d, J = 8.2 Hz, 2H), 7.04 (dd, J = 5.9, 2.2 Hz, 2H), 4.57 (s, 2H), 4.43 (s, 4H), 1.57 (s, 18H). ^{13}C NMR (101 MHz, MeOD) δ 168.9, 154.2, 153.6, 150.4, 141.1, 136.9, 133.1, 132.3, 131.1, 119.4, 113.6, 82.2, 59.1, 58.2, 28.6. HRMS (ESI): m/z $[M+H]^+$ calcd. for $[C_{30}H_{37}N_5O_6 +H^+]$: 564.2817; found: 564.2818; m/z $[M+Na^+]$ calcd. for $[C_{30}H_{37}N_5O_6 +Na^+]$: 586.2636; found: 586.2640. FT-IR (Vmax/cm^{-1}):3202(w), 2982(w), 2928(w), 2591(w), 1727(s), 1708(s), 1585(m), 1525(m), 1463(m), 1226(s), 1151(vs), 1082(m)

4-((Bis(pyridin-2-ylmethyl)amino)methyl)-N-methylbenzamide **12**

Methylamine hydrochloride (6.70 g; 99.2 mmol) was dissolved in methanol (15 ml) and NaOH(s)(3.86 g; 96.5 mmol) was added and the reaction mixture cooled on an ice bath. The mixture was stirred for 10 min at RT. Methyl 4-((bis(pyridin-2-ylmethyl)amino)methyl)benzoate (0.74 g; 2.1 mmol) was dissolved in methanol (5 ml) and added to the first suspension (final conc. 20% w/w % methyl amine). The reaction mixture was stirred at RT for 2 days in a sealed system with a balloon attached. The methanolic mix was filtered and the filtrate was evaporated over N_2 stream overnight. The solid was taken up in water (40 ml) and extracted with DCM (3x25 ml). The combined DCM phase was dried over $MgSO_4$, filtered and evaporated *in vacuo* to give a colourless oil. The oil was further purified by column chromatography (Eluent: 90:10, EtOAc/EtOH, Rf: 0.10) to give a colourless oil. Yield: 0.48 g (65%)

^{1}H NMR (400 MHz, $CDCl_3$) δ 8.49 (ddd, J = 4.9, 1.6, 1.0 Hz, 2H), 7.70 (d, J = 8.2 Hz, 2H), 7.64 (td, J = 7.7, 1.7 Hz, 2H), 7.53 (d, J = 7.8 Hz, 2H), 7.42 (d, J = 8.2 Hz, 2H), 7.13 (ddd, J = 7.2, 4.7, 0.9 Hz, 2H), 6.55 – 6.49 (m, 1H), 3.76 (s, 4H), 3.67 (s, 2H), 2.95 (d, J = 4.8 Hz, 3H). ^{13}C NMR (101 MHz, $CDCl_3$) δ 168.3, 159.4, 149.1, 142.7, 136.6, 133.6, 129.0, 127.0, 122.9, 122.2, 60.1, 58.2, 26.9. HRMS (ESI): m/z $[M+H]^+$ calcd. for $[C_{21}H_{22}N_4O +H^+]$: 347.1866; found: 347.1881. FT-IR (V_{max}/cm^{-1}):3449(w), 3280(m), 3063(w), 2931(w), 2818(w), 1637(s), 1547(s), 1432(m), 1302(s), 758(vs)

4-((Bis((6-methylpyridin-2-yl)methyl)amino)methyl)-N-methylbenzamide **13**

Methylamine hydrochloride (6.70 g; 99.2 mmol) was dissolved in methanol (15 ml) and NaOH(s) (3.86 g; 96.5 mmol) was added. The reaction mixture cooled on an ice bath and stirred for 10 min at RT. Methyl 4-((bis((6-methylpyridin-2-yl)methyl)amino)methyl)benzoate (0.85 g; 2.3 mmol) was dissolved in methanol (5 ml) and added to the suspension (final conc. 20% w/w % methyl amine). The reaction mixture was stirred at RT for 2 days in a sealed system with a balloon attached. The methanolic mix was filtered and the filtrate was evaporated over N_2 stream overnight. The solid was taken up in water (40 ml) and extracted with DCM (3x25 ml). The combined DCM phase was dried over $MgSO_4$, filtered and evaporated *in vacuo* to give an orange oil. The oil was further purified by column chromatography (Eluent: 90:10, EtOAc/EtOH, Rf: 0.21) to give a colourless oil. Yield: 0.57 g (67%)

^{1}H NMR (400 MHz, $CDCl_3$) δ 7.69 (d, J = 8.2 Hz, 2H), 7.53 (t, J = 7.7 Hz, 2H), 7.42 (d, J = 8.2 Hz, 2H), 7.38 (d, J = 7.7 Hz, 2H), 6.97 (d, J = 7.5 Hz, 2H), 6.58 – 6.50 (m, 1H), 3.72 (s, 4H), 3.66 (s, 2H), 2.94 (d, J = 4.8 Hz, 3H), 2.48 (s, 6H). ^{13}C NMR (101 MHz, $CDCl_3$) δ 168.3, 159.0, 157.6, 143.0, 136.8, 133.5, 128.9, 127.0, 121.6, 119.5, 60.2, 58.1, 26.8, 24.5. HRMS (ESI): m/z $[M+H]^+$ calcd. for $[C_{23}H_{26}N_4O +H^+]$: 375.2179; found: 375.2197 107 FT-IR (V_{max}/cm^{-1}): 3454(w), 3317(w), 3062(w), 2922(w), 2823(w), 1639(s), 1578(m), 1547(s), 1455(s), 1302(m), 787(m), 751(s)

Di-tert-butyl ((((4-(methylcarbamoyl)benzyl)azanediyl)bis(methylene))bis(pyridine-6,2-diyl))dicarbamate

Methylamine hydrochloride (6.70 g; 99.2 mmol) was dissolved in ethanol (15 ml) and NaOH(s) (3.86 g; 96.5 mmol) was added and the reaction mixture cooled on an ice bath. The mixture was stirred for 10 min at RT. Methyl 4-((bis((6-((tertbutoxycarbonyl)amino)pyridin-2-yl)methyl)amino)methyl)-benzoate (0.260 g; 0.450 mmol) was dissolved in ethanol (5 ml) and added to the first suspension (final conc. 20% w/w % methyl amine) The reaction mixture was stirred at 40 °C for 4 days in a sealed system with a balloon attached. The ethanolic mix was filtered and the filtrate was evaporated under a N_2 stream overnight. The residual solid was taken up in water (40 ml) and extracted with DCM (3x25 ml). The combined DCM phase was washed with brine (20 ml), dried over $MgSO_4$, filtered and evaporated *in vacuo* to give a white solid. The solid was further purified by column chromatography (Eluent: EtOAc/Hex, 60:40, Rf: 0.23) to give a white solid. Yield: 0.115 g (44%) mp: 105-107 °C

^{1}H NMR (400 MHz, $CDCl_3$) δ 7.76 (d, J = 8.2 Hz, 2H), 7.68 – 7.59 (m, 4H), 7.45 (s, 2H), 7.41 (d, J = 8.1 Hz, 2H), 7.15 (d, J = 7.4 Hz, 2H), 6.27 – 6.17 (m,1H), 3.66 (s, 2H), 3.64 (s, 4H), 2.99 (d, J = 4.8 Hz, 3H), 1.48 (s, 18H). ^{13}C NMR (101 MHz, $CDCl_3$) δ 168.3, 157.8, 152.5, 151.3, 142.7, 138.7, 133.6, 129.0, 127.0, 117.7, 110.5, 81.0, 59.5, 57.9, 28.4, 26.9. HRMS (ESI): m/z [M+H]+ calcd. for [$C_{31}H_{40}N_6O_5$ +H^+]: 577.3133; found: 577.3143; m/z [M+Na^+] calcd. for [$C_{31}H_{40}N_6O_5$ +Na^+]: 599.2952; found: 599.2953. FT-IR (Vmax/cm^{-1}):3448-3153(w), 2977(w), 2928(w),2805(w), 1726(s), 1642(w), 1579(m), 1518(m), 1456(s), 1407(m), 1366(m), 1277(m), 1226(s), 1150(vs), 1082(m), 797(m), 753(m)

4-((Bis((6-aminopyridin-2-yl)methyl)amino)methyl)-N-methylbenzamide **14**

Methyl 4-((bis((6-((tert-butoxycarbonyl)amino)pyridin-2-yl)methyl)amino)methyl)-benzoate (109 mg; 0.189 mmol) was taken up in 20% TFA in DCM (1:4) (5 ml) and the reaction mixture was stirred at 25°C for 16h. The reaction mixture was evaporated over an N_2 stream and the residue was taken up in DCM (20 ml). The DCM solution was washed with $NaHCO_3$ (sat.) (2x20 ml) and the combined water phases were extracted with DCM (20 ml). The combined organic phase was dried over $MgSO_4$, filtered and evaporated *in vacuo* to give a white solid. Yield: 59.8 mg (84%) Mp: 94-96 °C

^{1}H NMR (400 MHz, MeOD) δ 7.75 (d, J = 9.9 Hz, 2H), 7.48 (d, J = 8.2 Hz, 2H), 7.42 (t, J = 7.8 Hz, 2H), 6.88 (d, J = 7.3 Hz, 2H), 6.42 (d, J = 8.2 Hz, 2H), 3.66 (s, 2H), 3.53 (s, 4H), 2.90 (s, 3H). ^{13}C NMR (101 MHz, MeOD) δ 170.6, 160.4, 158.2, 144.3, 139.7, 134.3, 129.9, 128.2, 112.7, 108.4, 60.6, 59.1, 26.9. HRMS (ESI): m/z [M+H]$^+$ calcd. for [$C_{21}H_{24}N_6O$ +H^+]: 377.2084; found: 377.2095. FT-IR (Vmax/cm^{-1}):3441(w), 3322(m), 3188(w),2813(w), 1614(s), 1598(m), 1570(s), 1460(s), 1294(m)

NMR Spectra

(6-Methylpyridin-2-yl)methyl methanesulfonate
^{1}H NMR (400 MHz, $CDCl_3$)

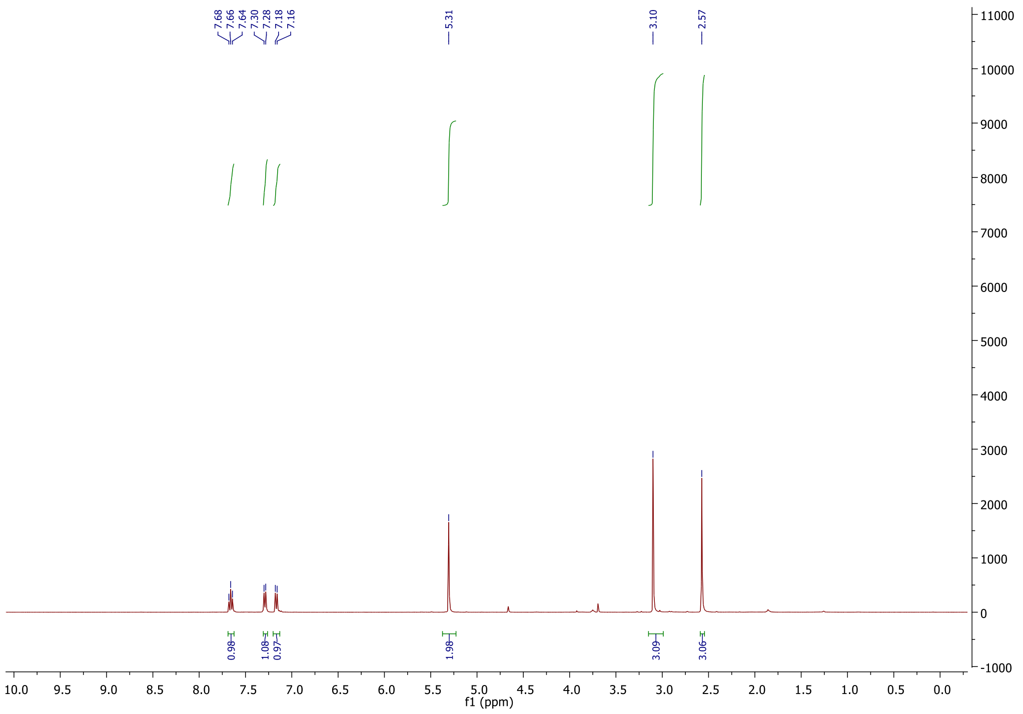

^{13}C NMR (101 MHz, $CDCl_3$)

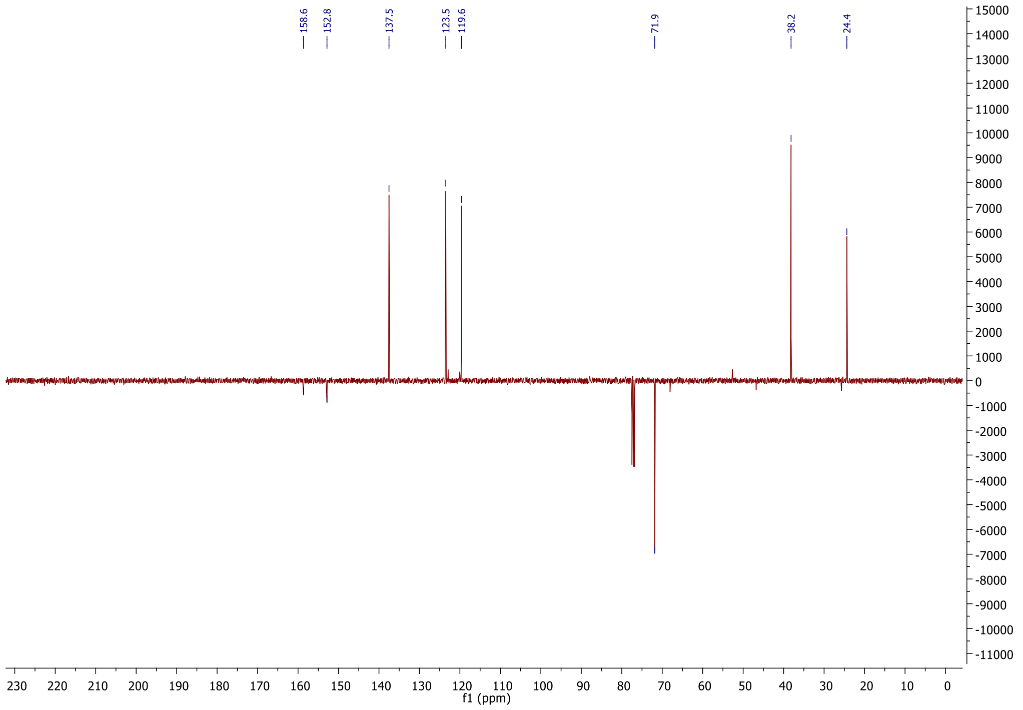

2-(Iodomethyl)-6-methylpyridine

^{1}H NMR (400 MHz, $CDCl_3$)

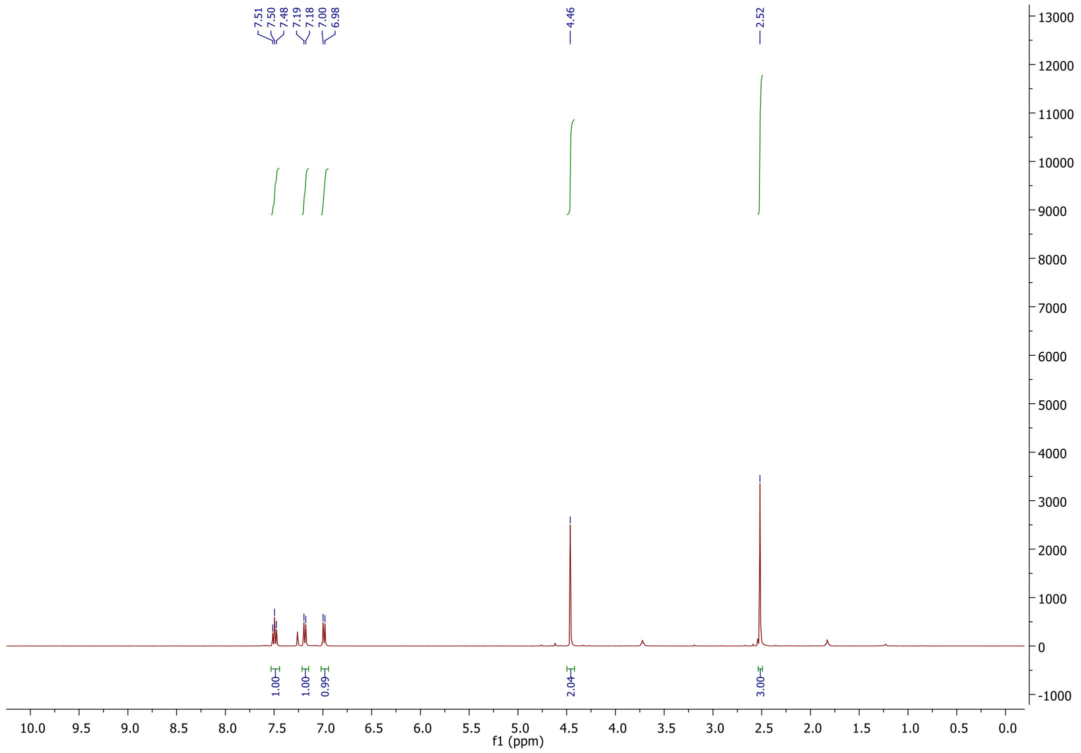

^{13}C NMR (101 MHz, $CDCl_3$)

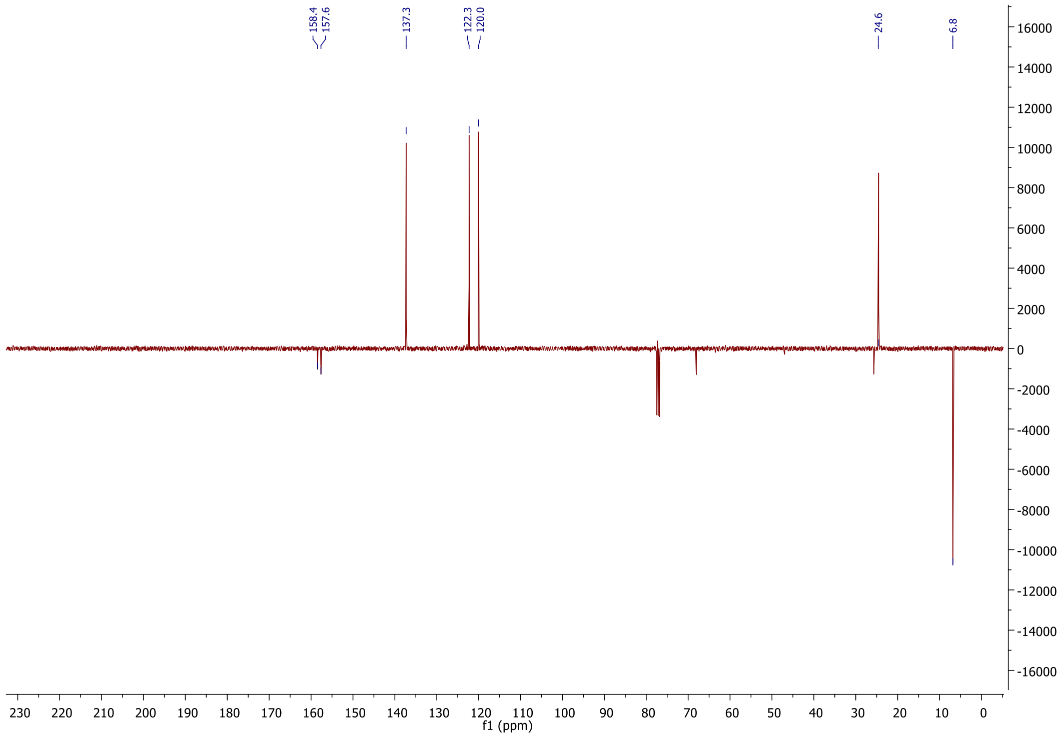

Diethyl pyridine-2,6-dicarboxylate **3**
^{1}H NMR (400 MHz, $CDCl_3$)

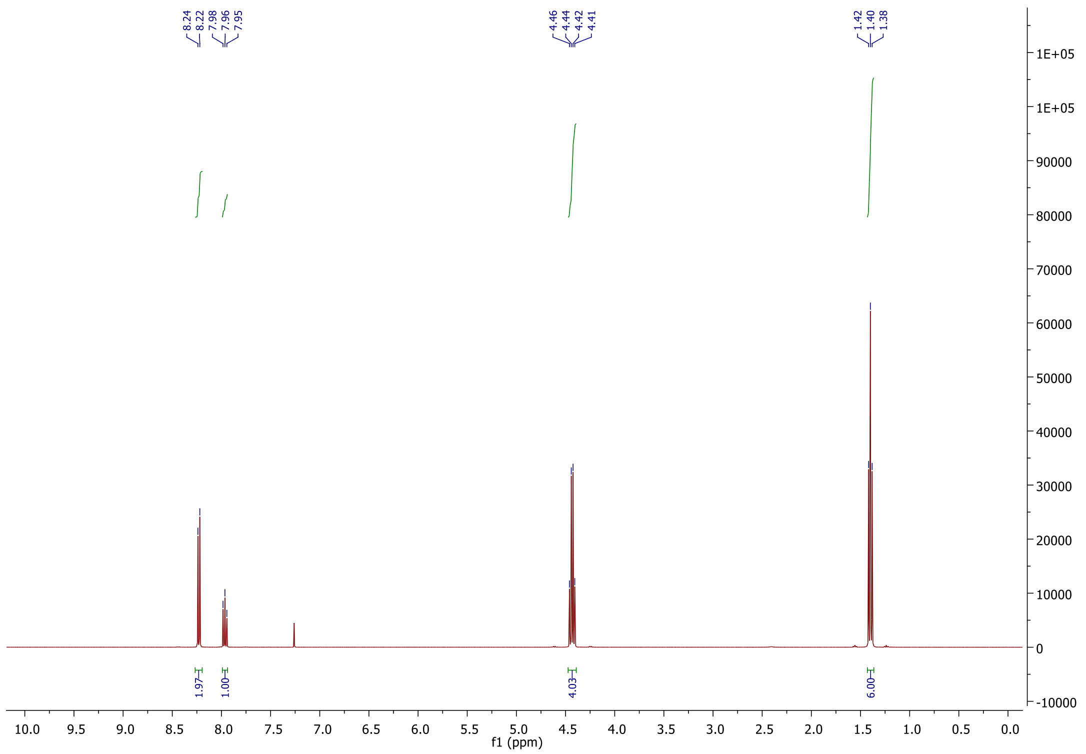

^{13}C NMR (101 MHz, $CDCl_3$)

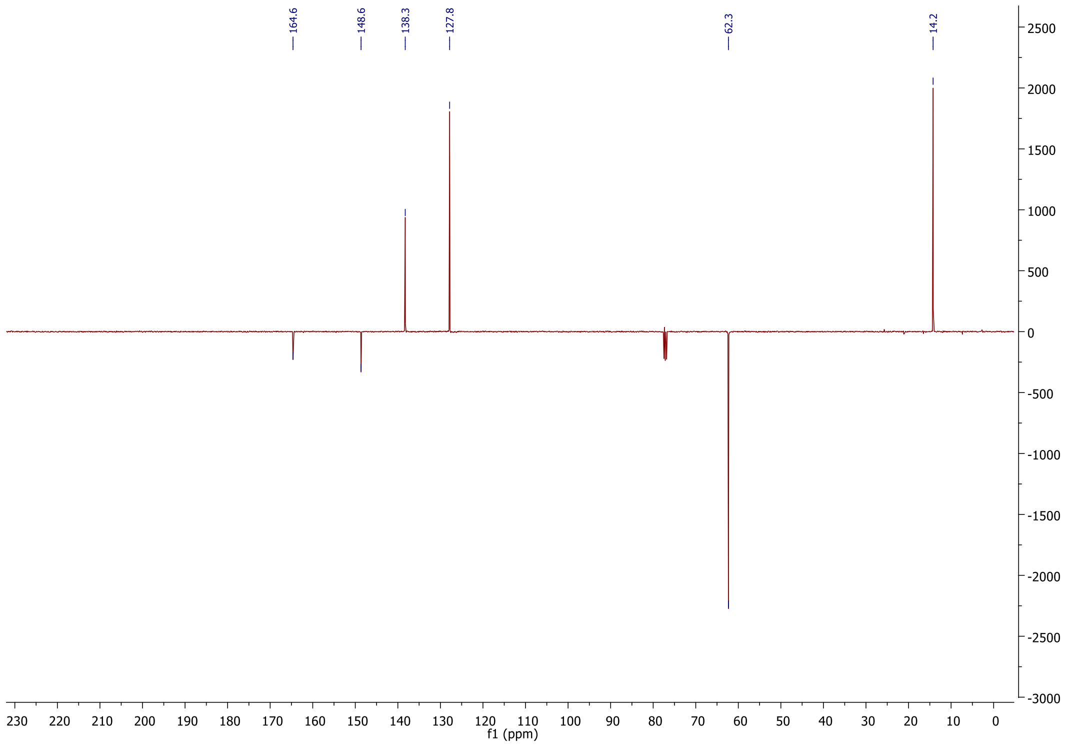

6-(Ethoxycarbonyl)picolinic acid **4**
^{1}H NMR (400 MHz, $CDCl_3$)

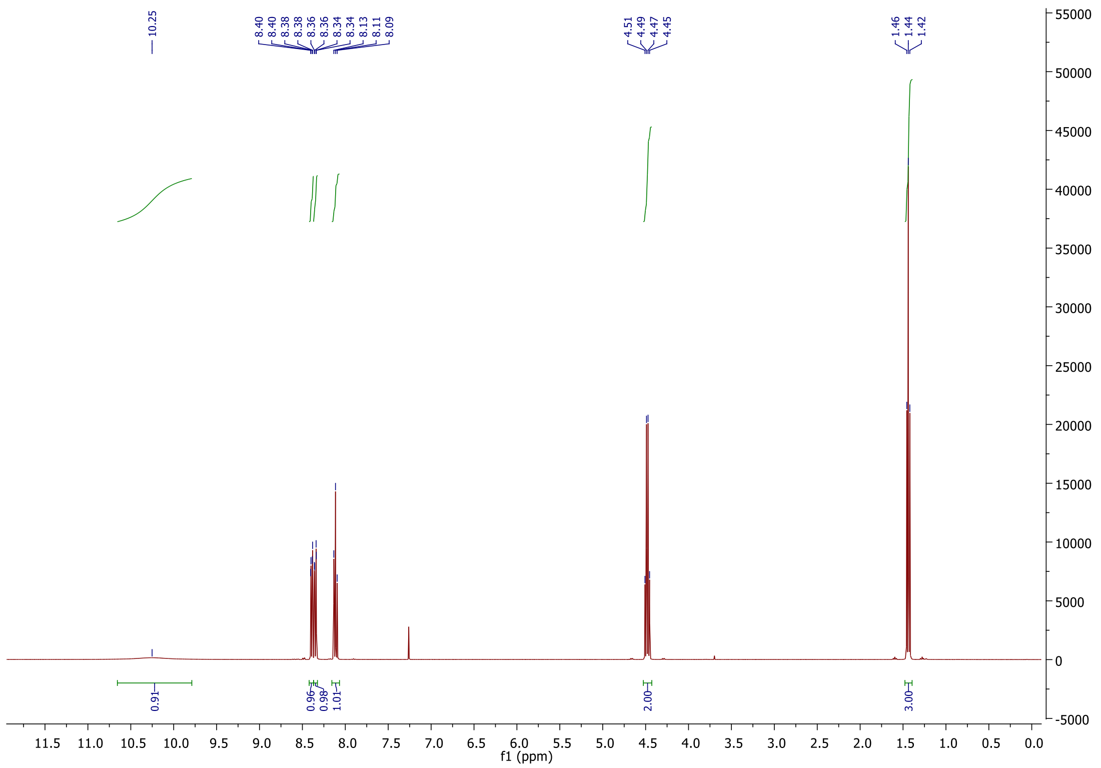

^{13}C NMR (101 MHz, $CDCl_3$)

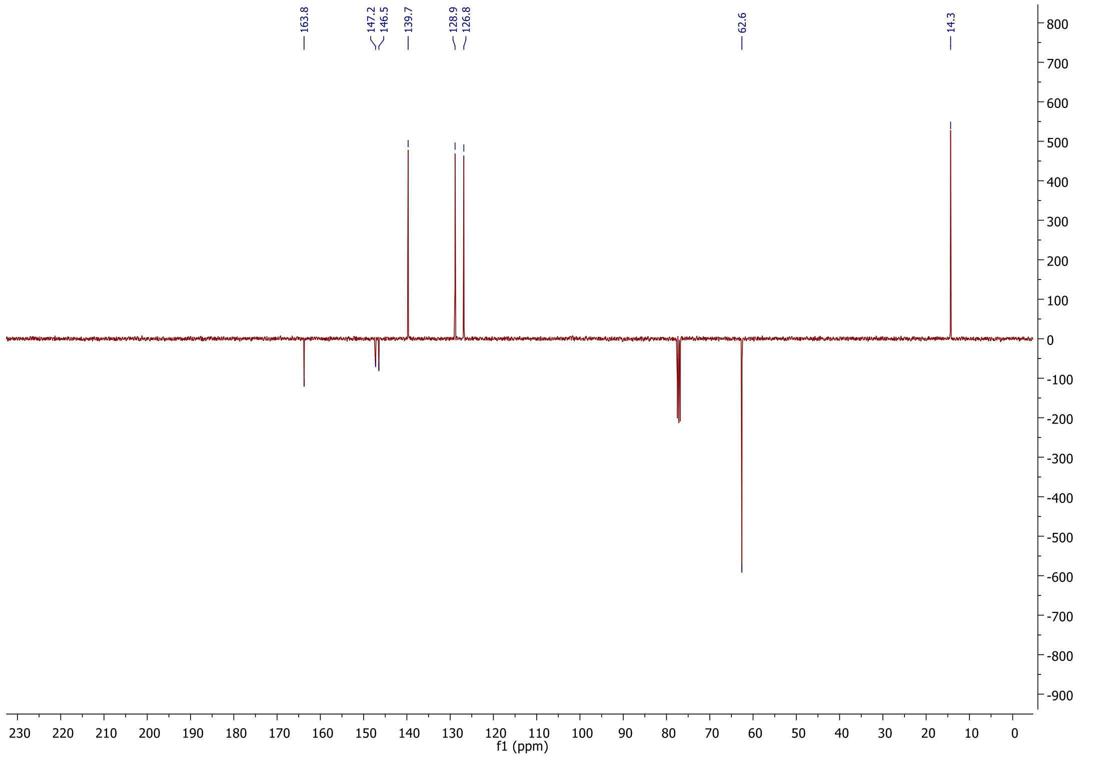

Ethyl 6-((tert-butoxycarbonyl)amino)picolinate **5**
^{1}H NMR (400 MHz, $CDCl_3$)

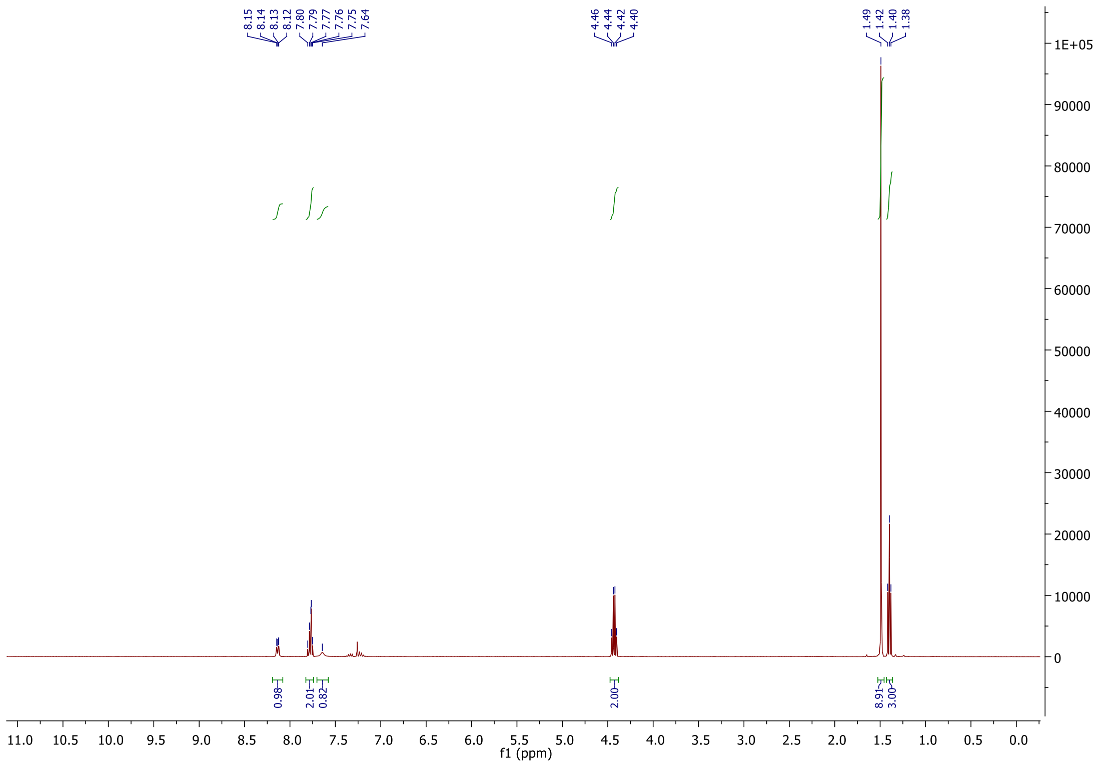

^{13}C NMR (101 MHz, $CDCl_3$)

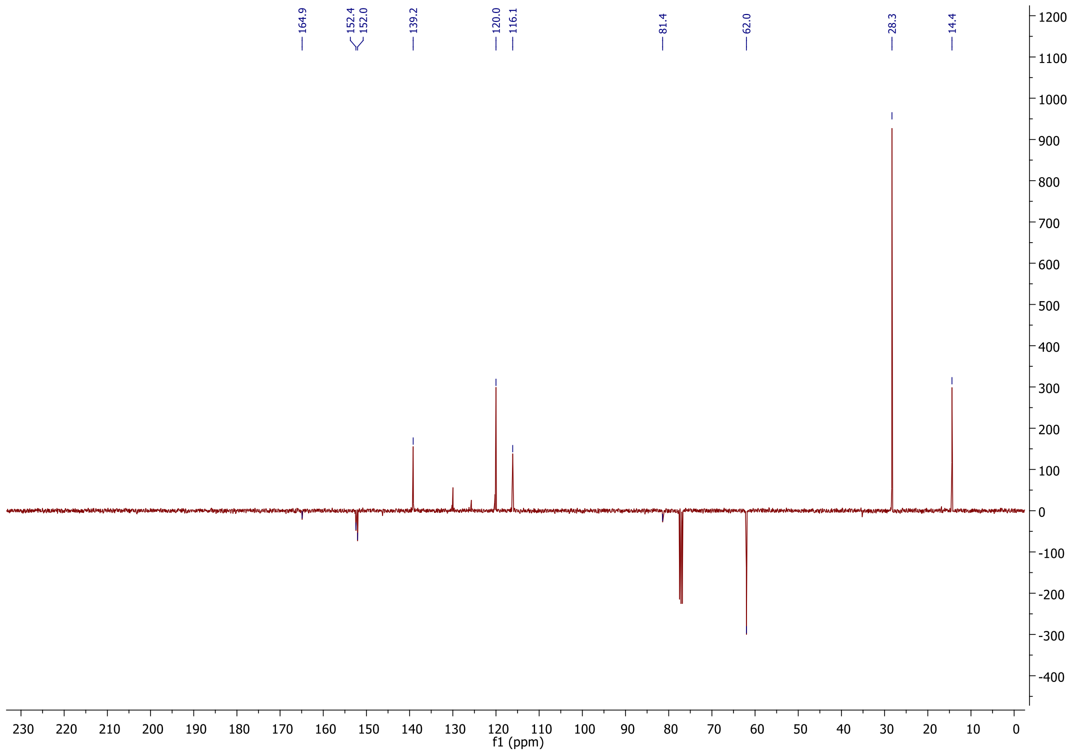

Tert-butyl (6-(hydroxymethyl)pyridin-2-yl)carbamate **1**

^{1}H NMR (400 MHz, $CDCl_3$)

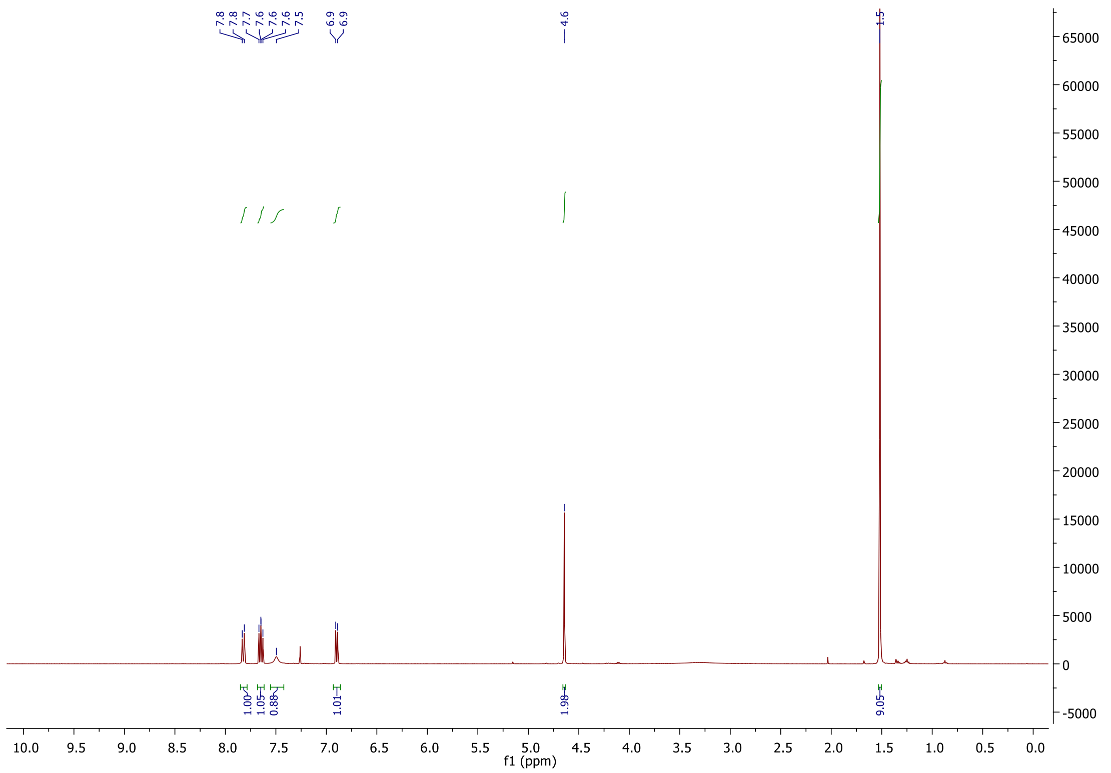

^{13}C NMR (101 MHz, $CDCl_3$)

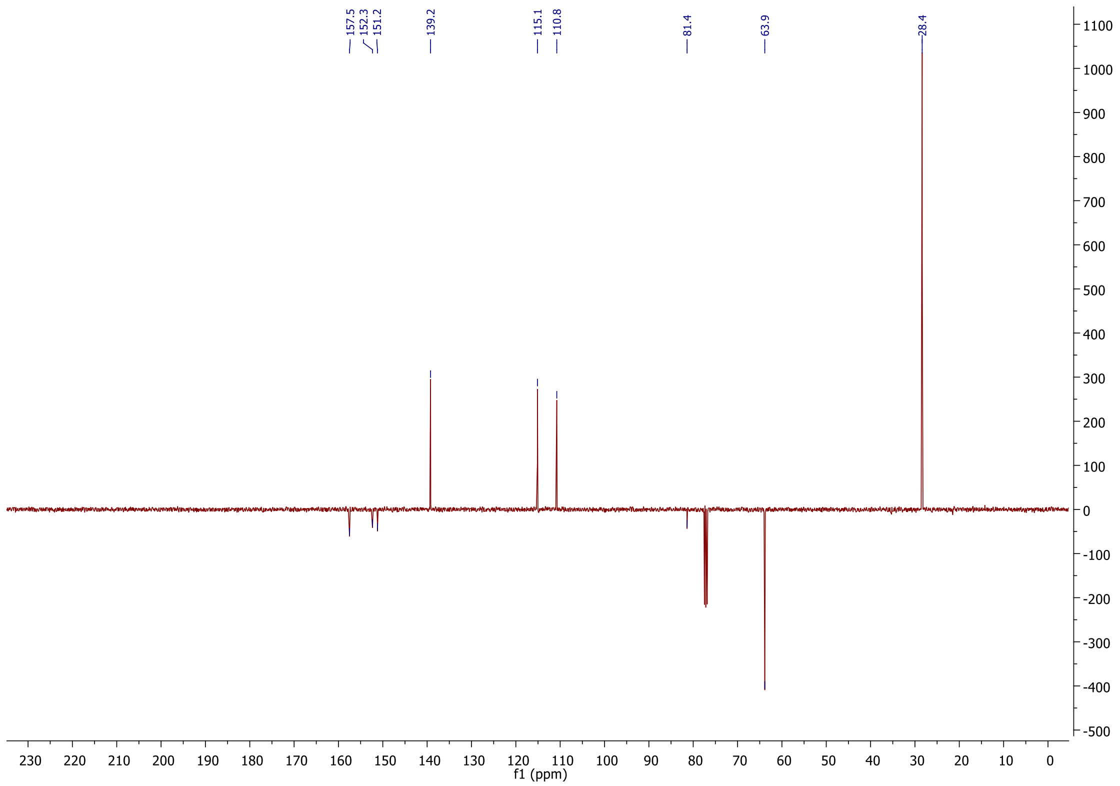

(6-((Tert-butoxycarbonyl)amino)pyridin-2-yl)methyl methanesulfonate **6**

^{1}H NMR (400 MHz, $CDCl_3$)

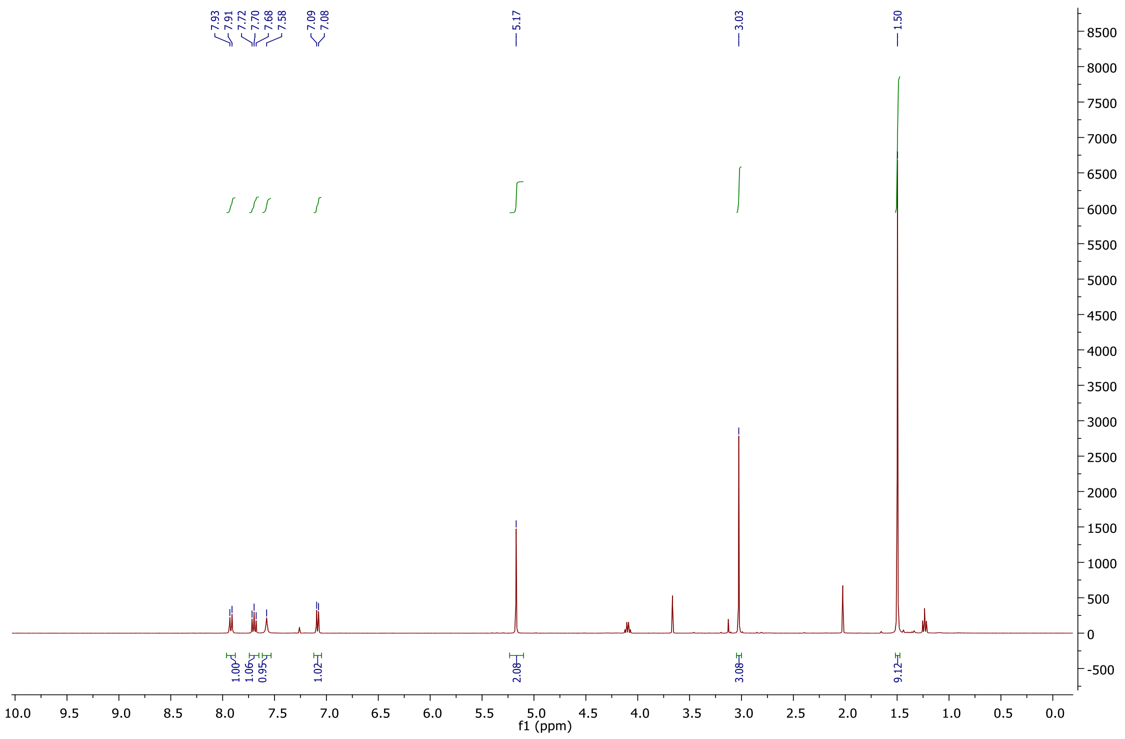

^{13}C NMR (101 MHz, $CDCl_3$)

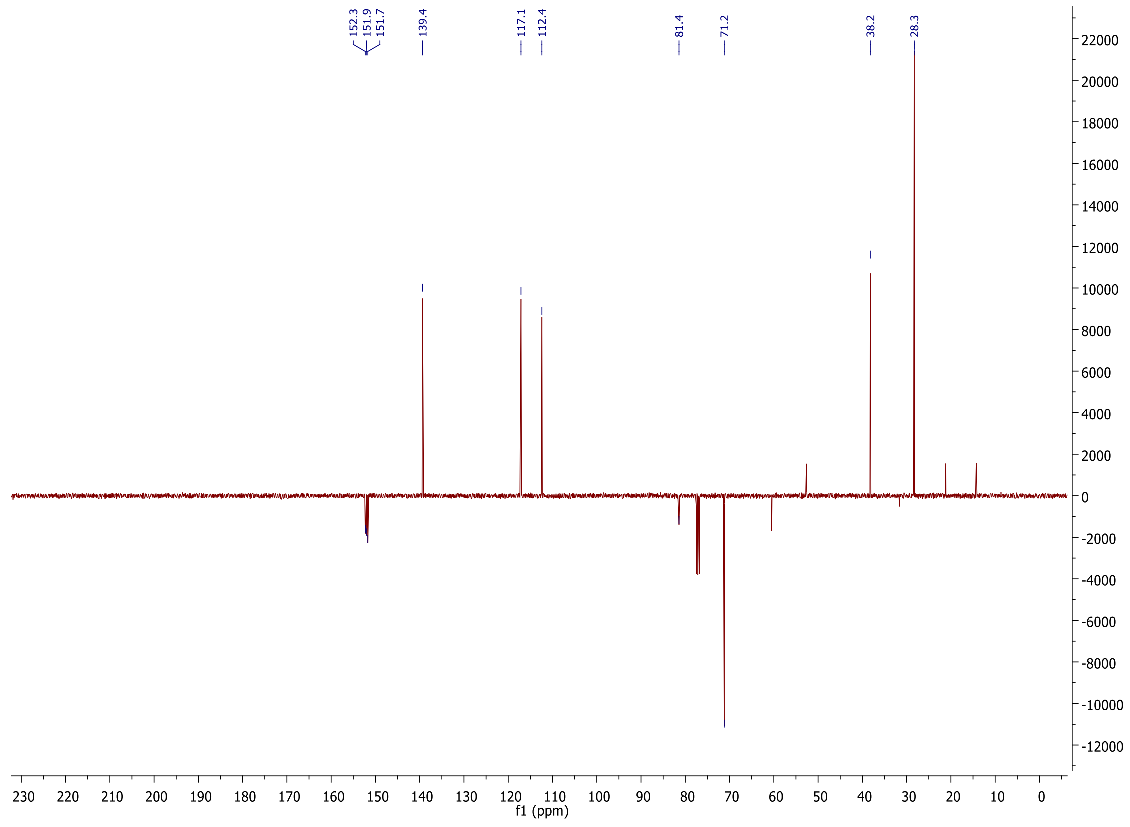

Tert-butyl (6-(iodomethyl)pyridin-2-yl)carbamate **7**

^{1}H NMR (400 MHz, $CDCl_3$)

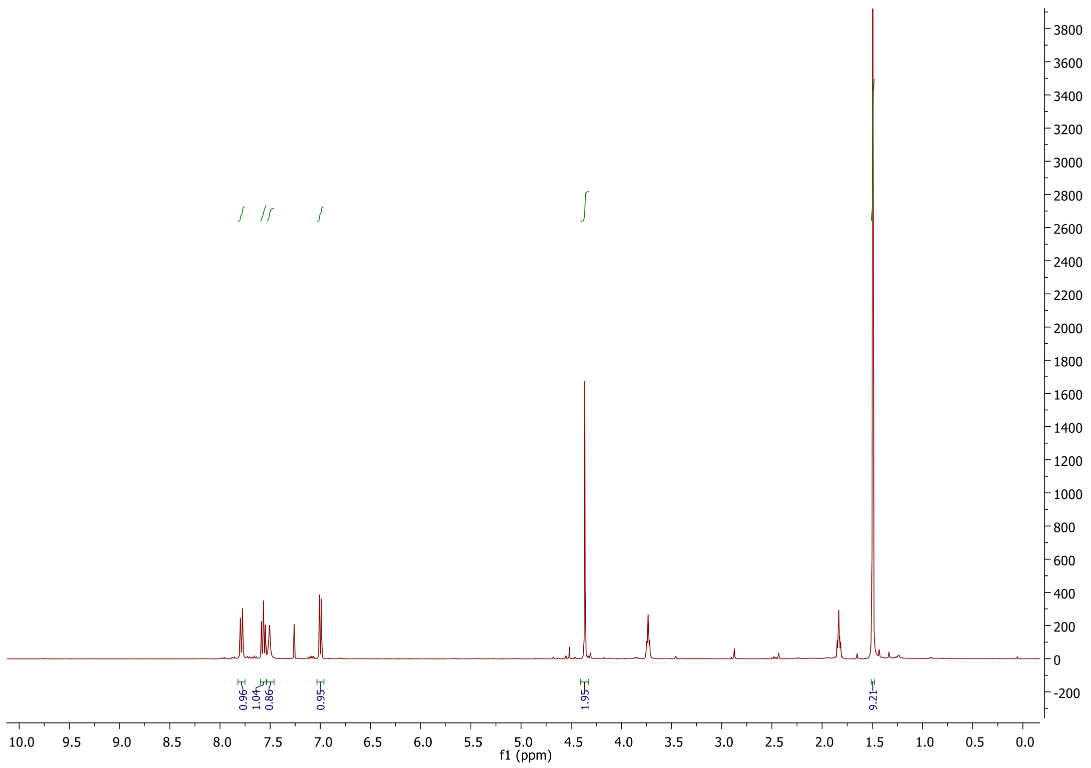

^{13}C NMR (101 MHz, $CDCl_3$)

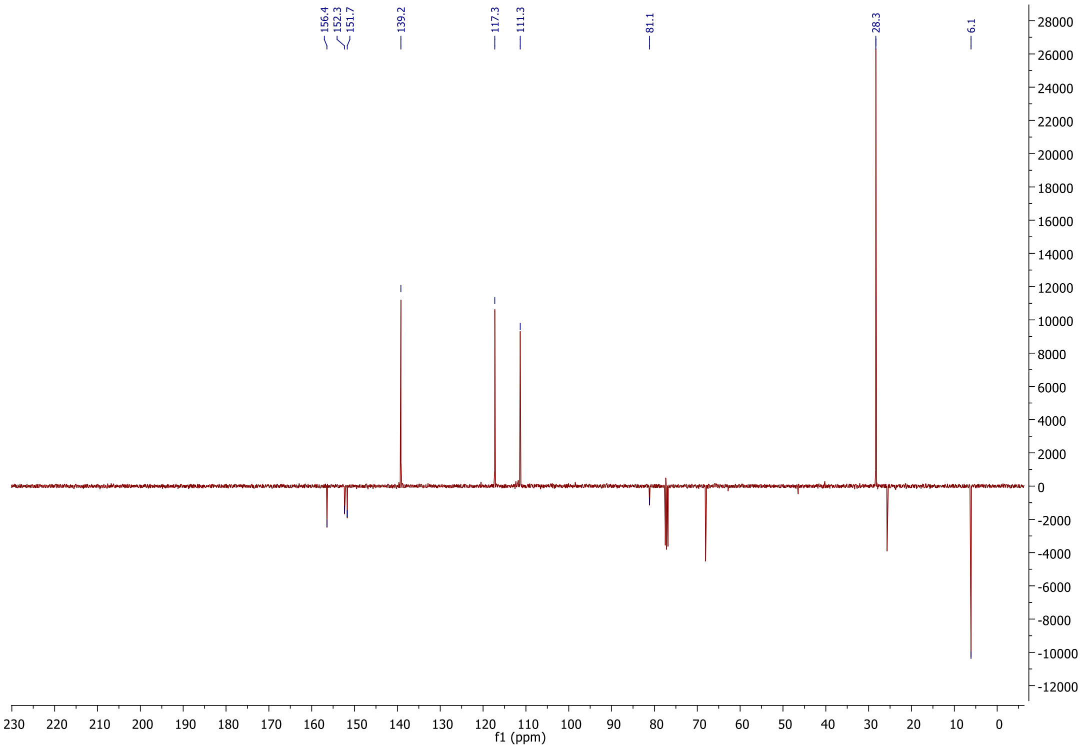

Methyl 4-(aminomethyl)benzoate hydrochloride **8**
^{1}H NMR (400 MHz, DMSO-d6)

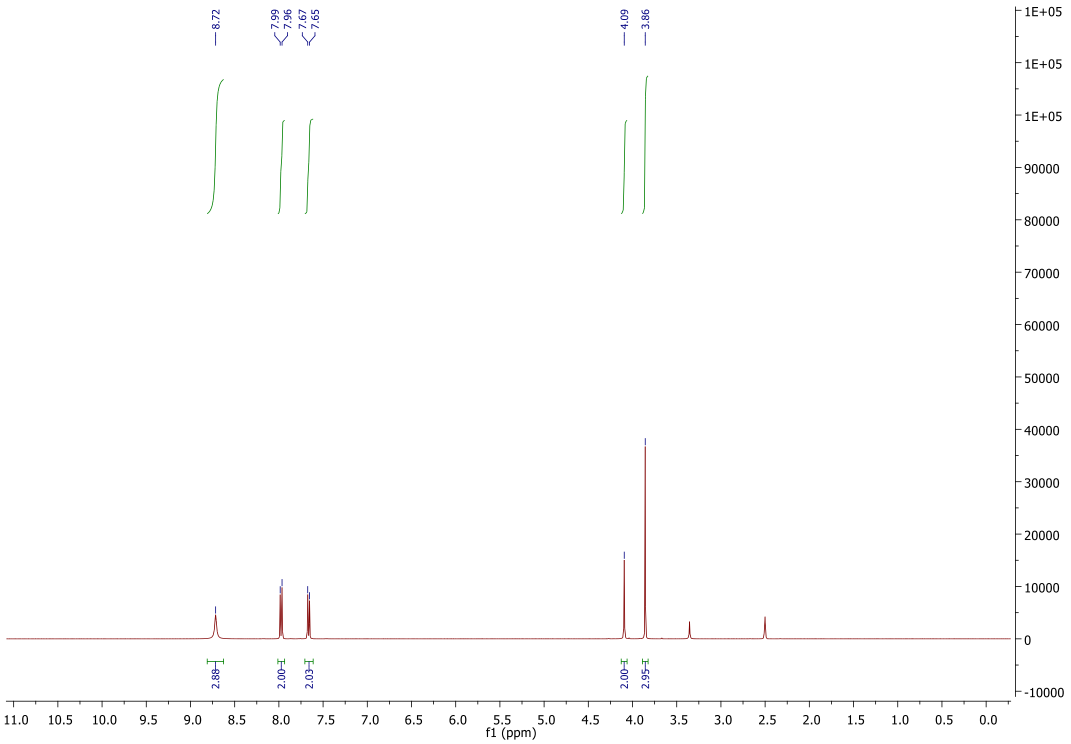

^{13}C NMR (101 MHz, DMSO-d6)

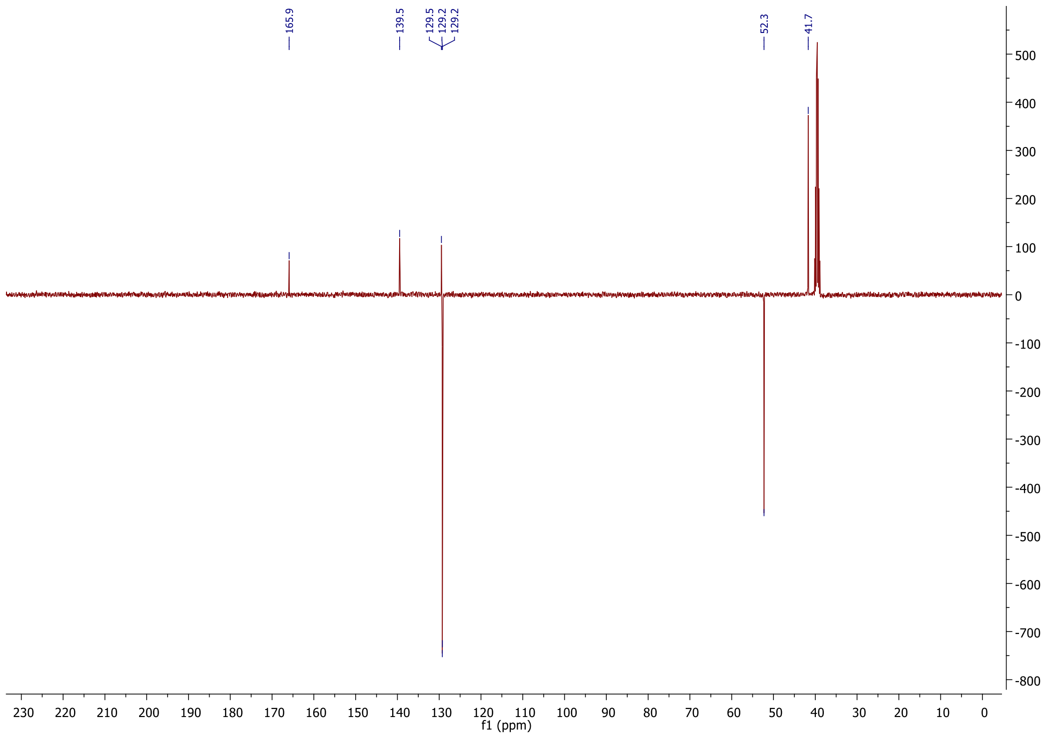

Methyl 4-((bis(pyridin-2-ylmethyl)amino)methyl)benzoate
^{1}H NMR (400 MHz, $CDCl_3$)

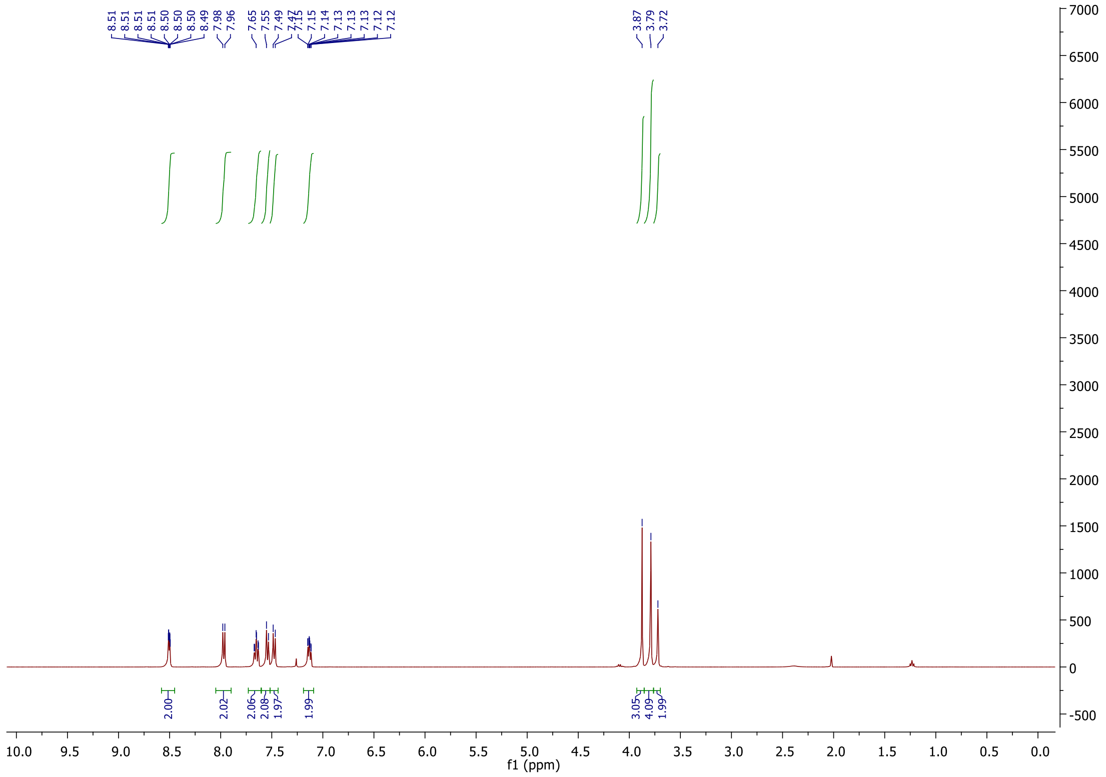

^{13}C NMR (101 MHz, $CDCl_3$)

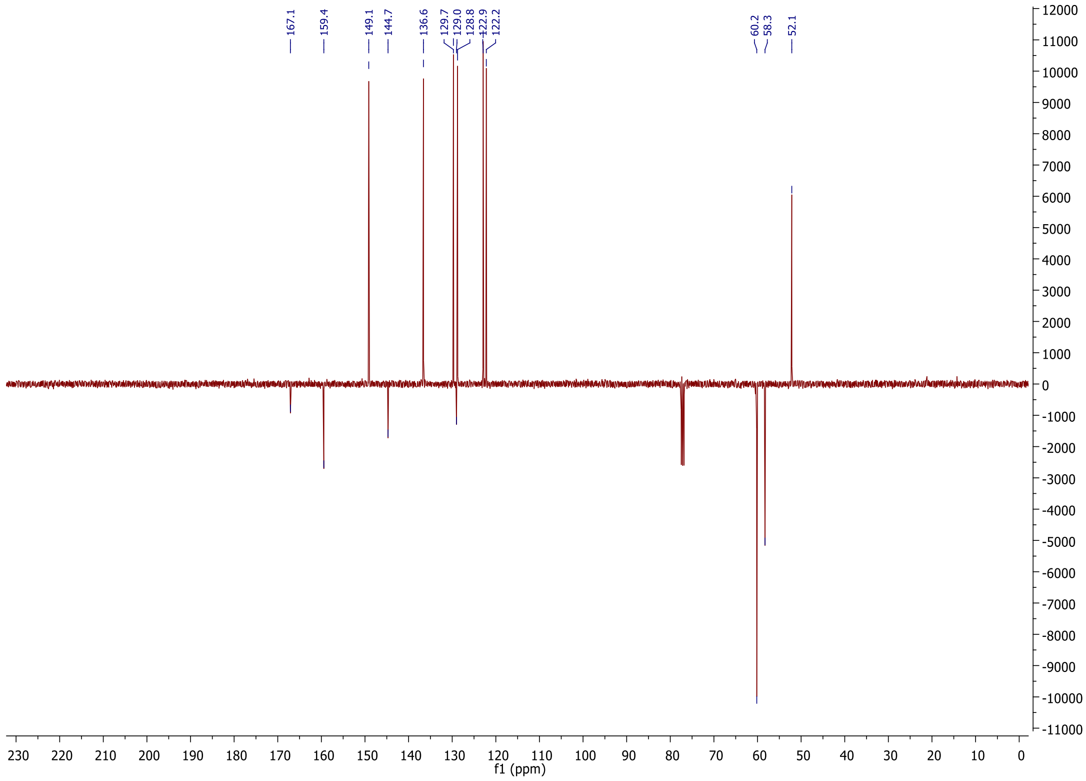

Methyl 4-((bis((6-methylpyridin-2-yl)methyl)amino)methyl)benzoate

^{1}H NMR (400 MHz, $CDCl_3$)

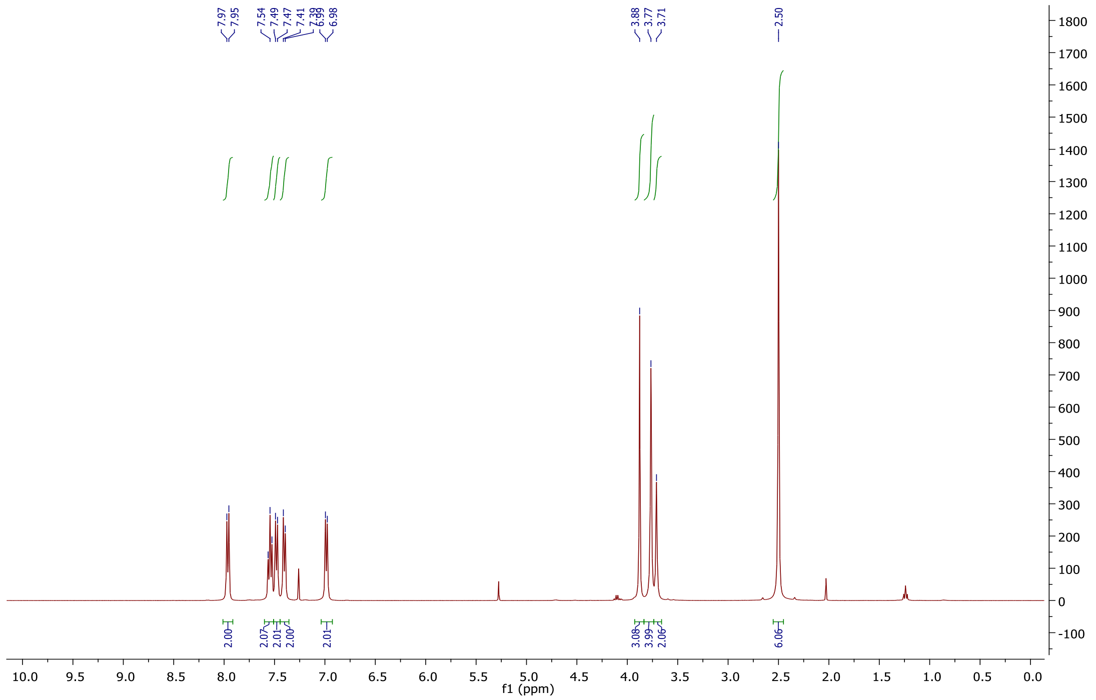

^{13}C NMR (101 MHz, $CDCl_3$)

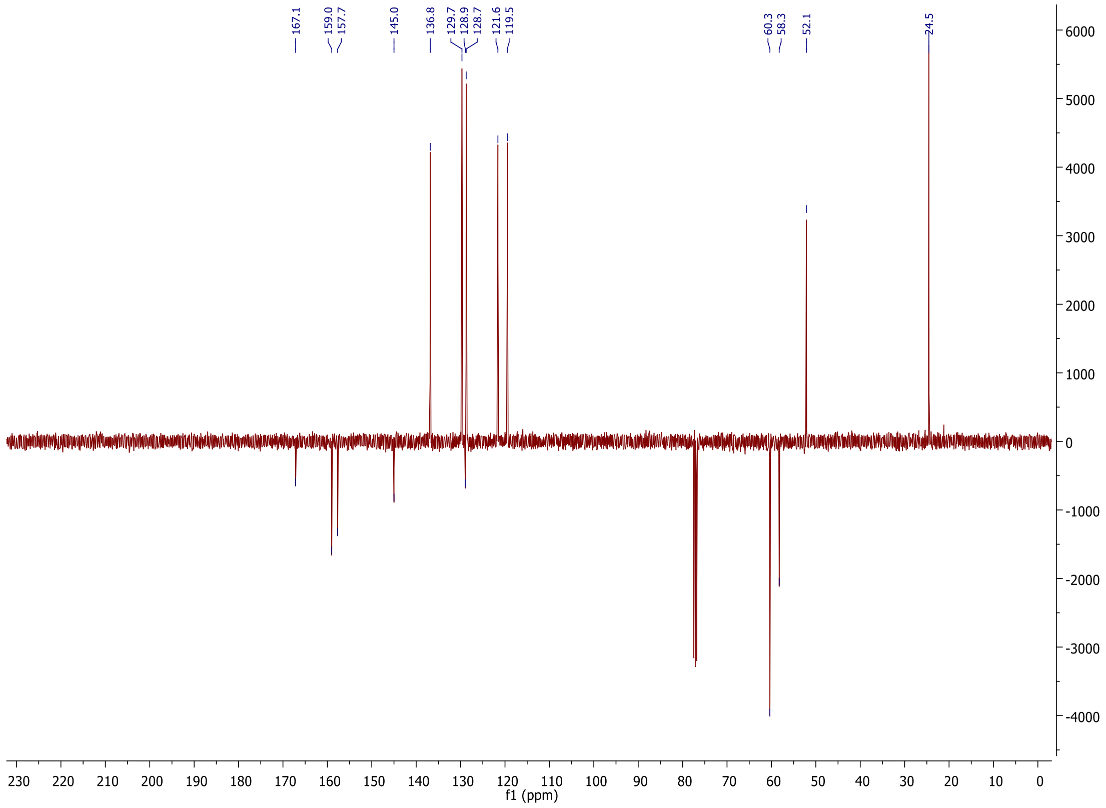

Methyl 4-((bis((6-((tert-butoxycarbonyl)amino)pyridin-2-yl)methyl)amino)methyl)-benzoate

^{1}H NMR (400 MHz, $CDCl_3$)

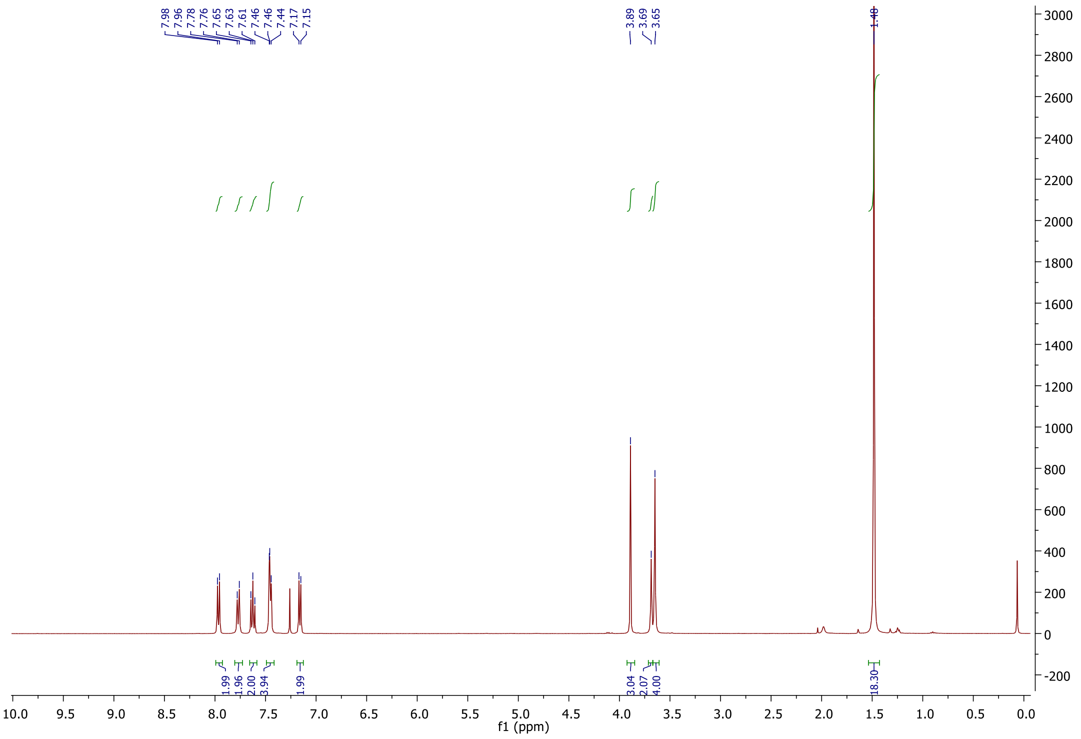

^{13}C NMR (101 MHz, $CDCl_3$)

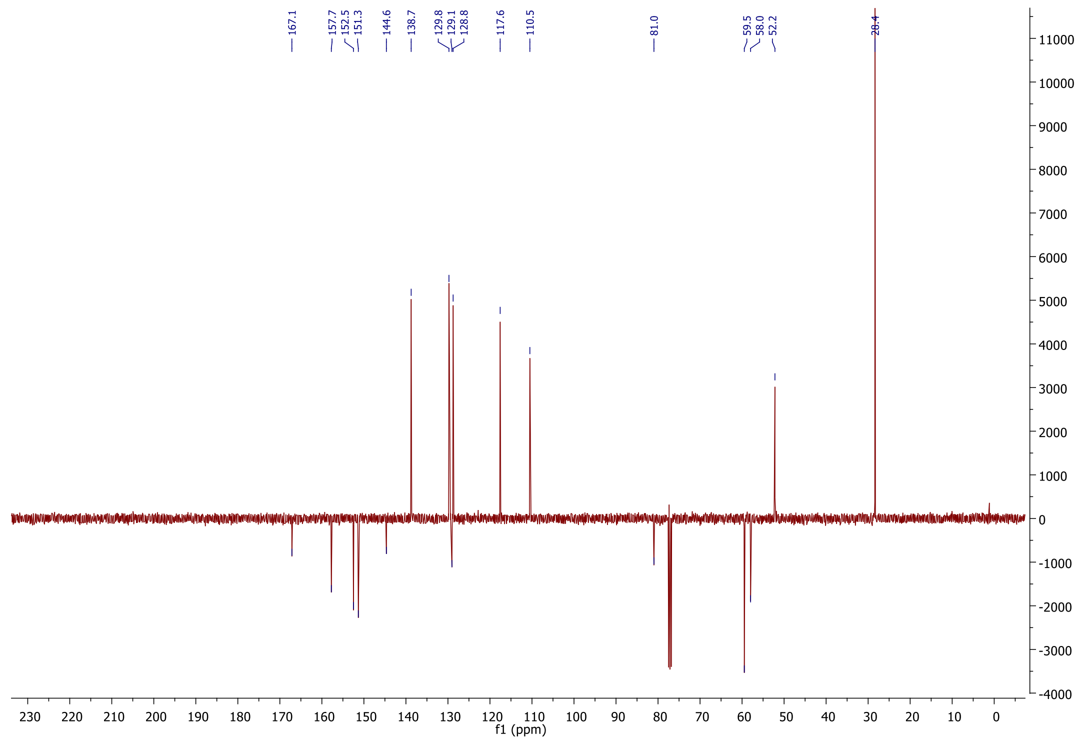

4-((bis(pyridin-2-ylmethyl)amino)methyl)benzoic acid **9**

^{1}H NMR (400 MHz, DMSO-d6)

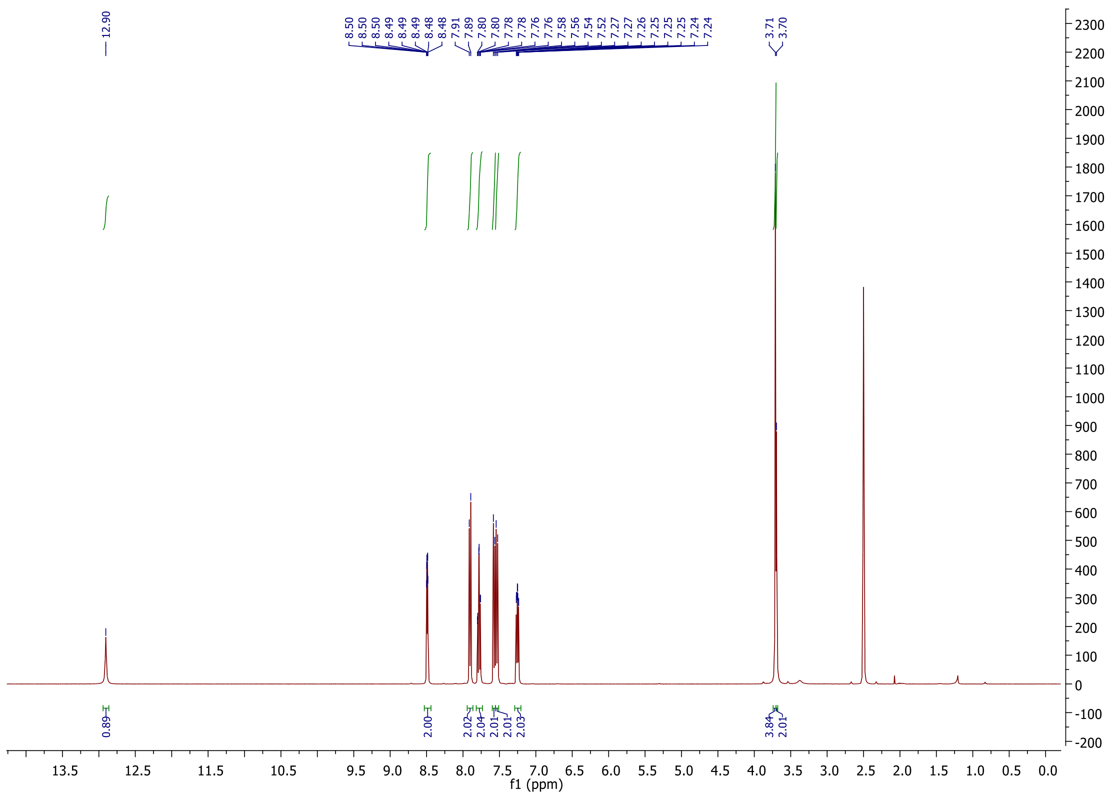

^{13}C NMR (101 MHz, DMSO-d6)

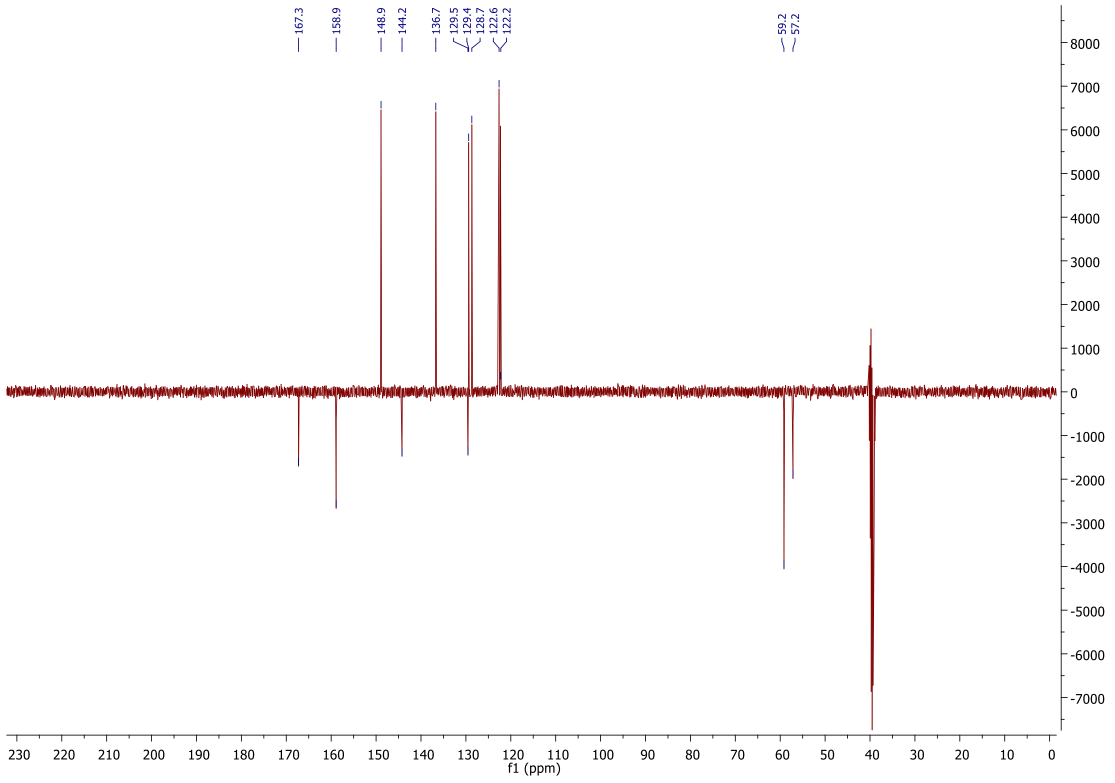

4-((bis((6-methylpyridin-2-yl)methyl)amino)methyl)benzoic acid **10**
^{1}H NMR (400 MHz, DMSO-d6)

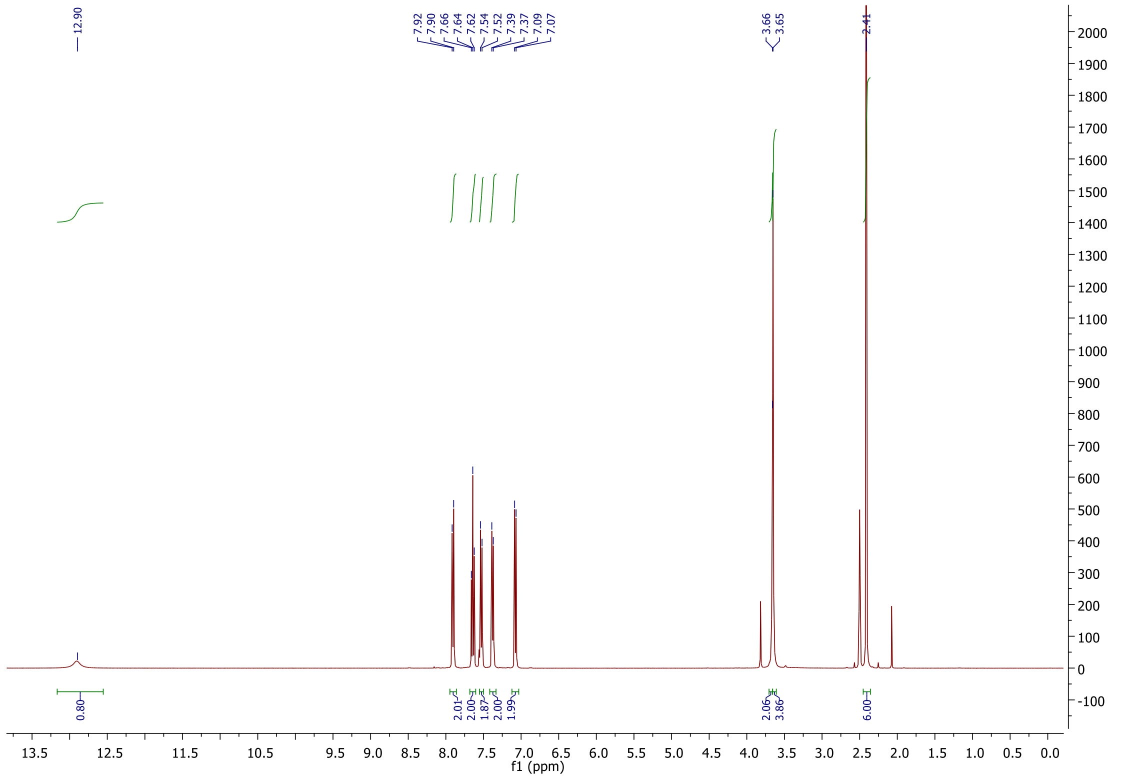

^{13}C NMR (101 MHz, DMSO-d6)

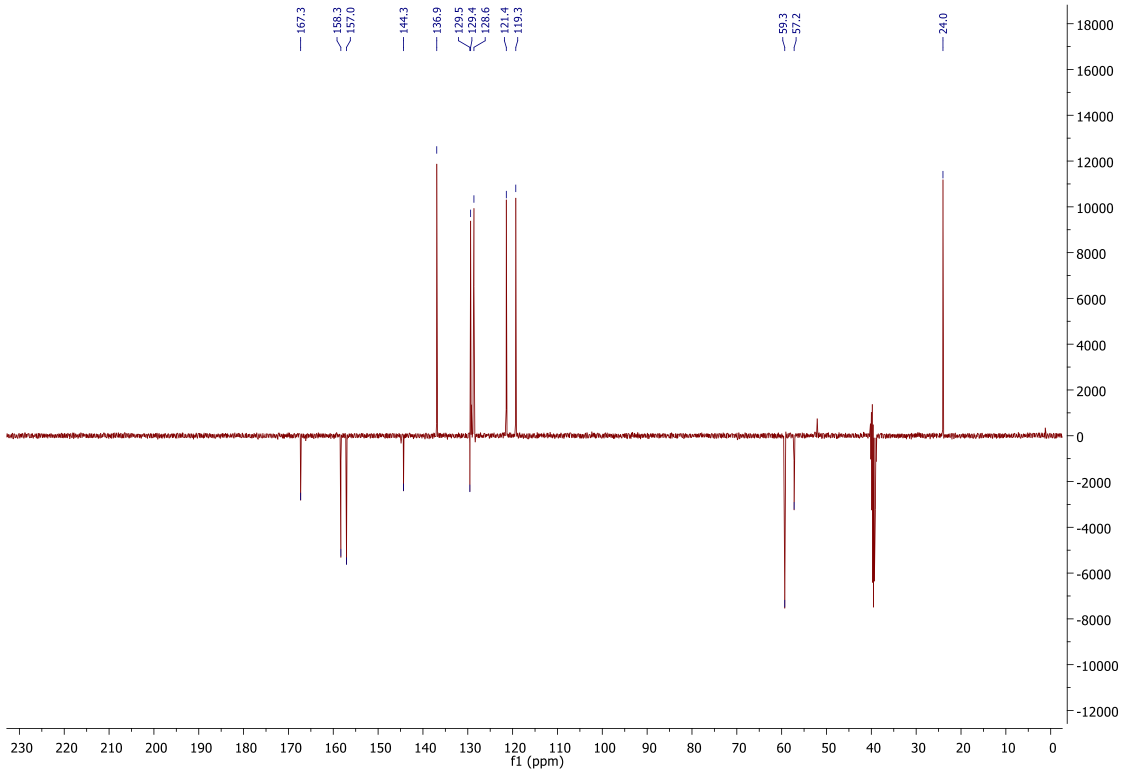

4-((bis((6-((tert-butoxycarbonyl)amino)pyridin-2-yl)methyl)amino)methyl)benzoic acid **11** ^{1}H NMR (400 MHz, MeOD-d4)

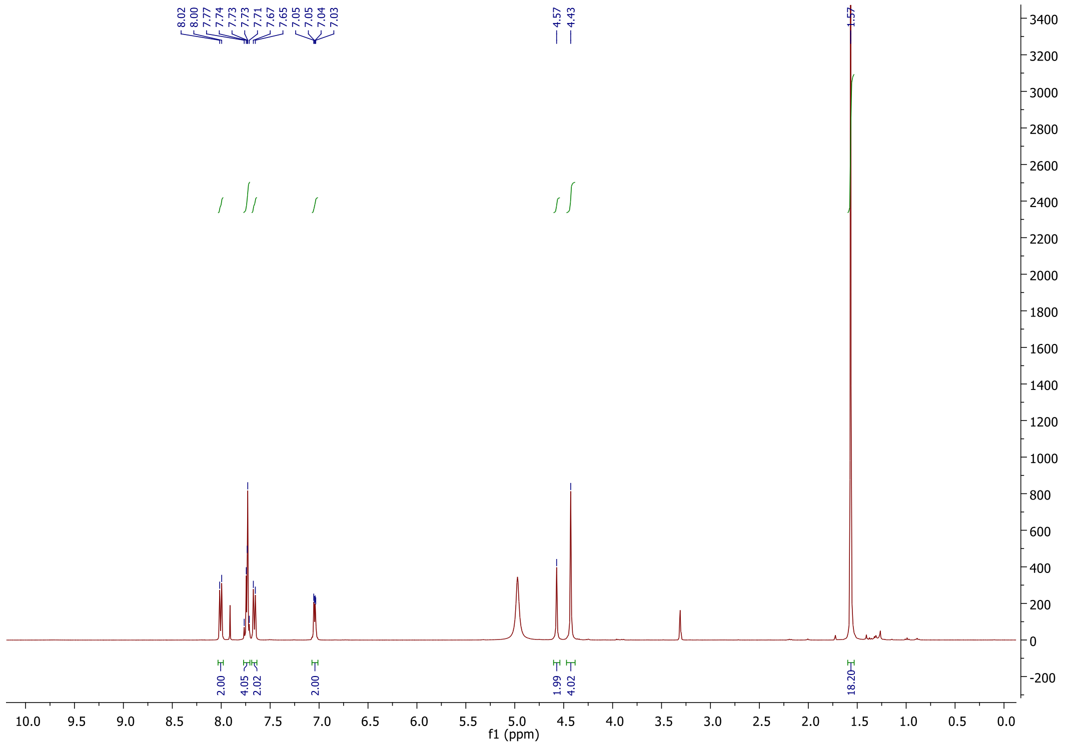

^{13}C NMR (101 MHz, MeOD-d4)

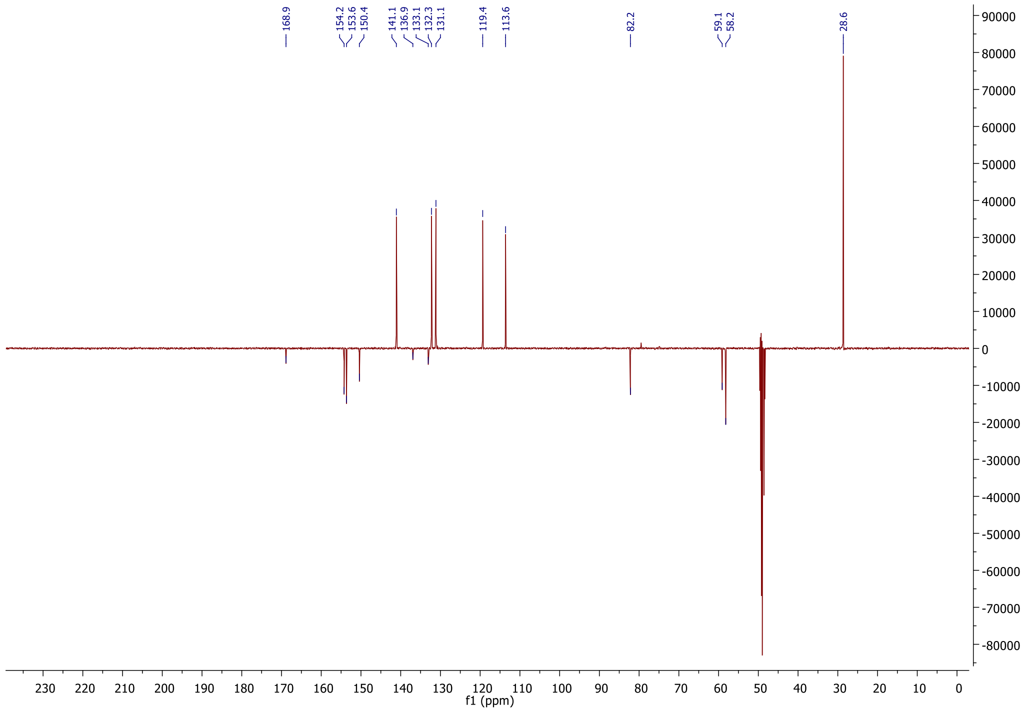

4-((bis(pyridin-2-ylmethyl)amino)methyl)-N-methylbenzamide **12**

^{1}H NMR (400 MHz, $CDCl_3$)

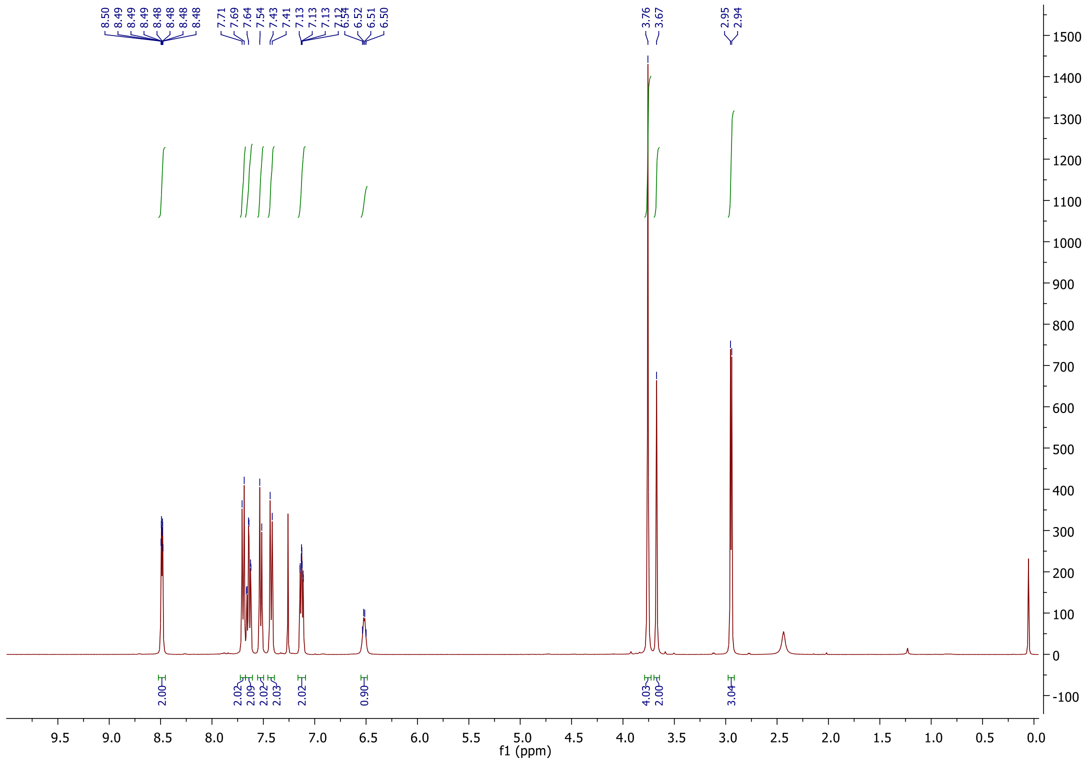

^{13}C NMR (101 MHz, $CDCl_3$)

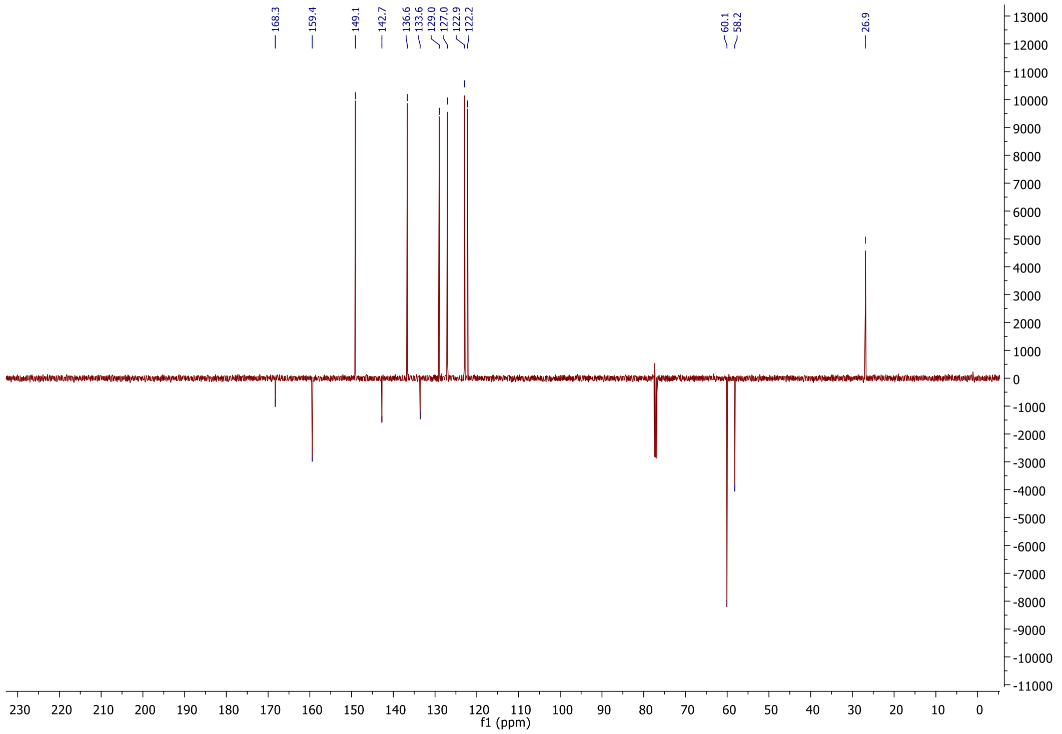

4-((bis((6-methylpyridin-2-yl)methyl)amino)methyl)-N-methylbenzamide **13**
^{1}H NMR (400 MHz, $CDCl_3$)

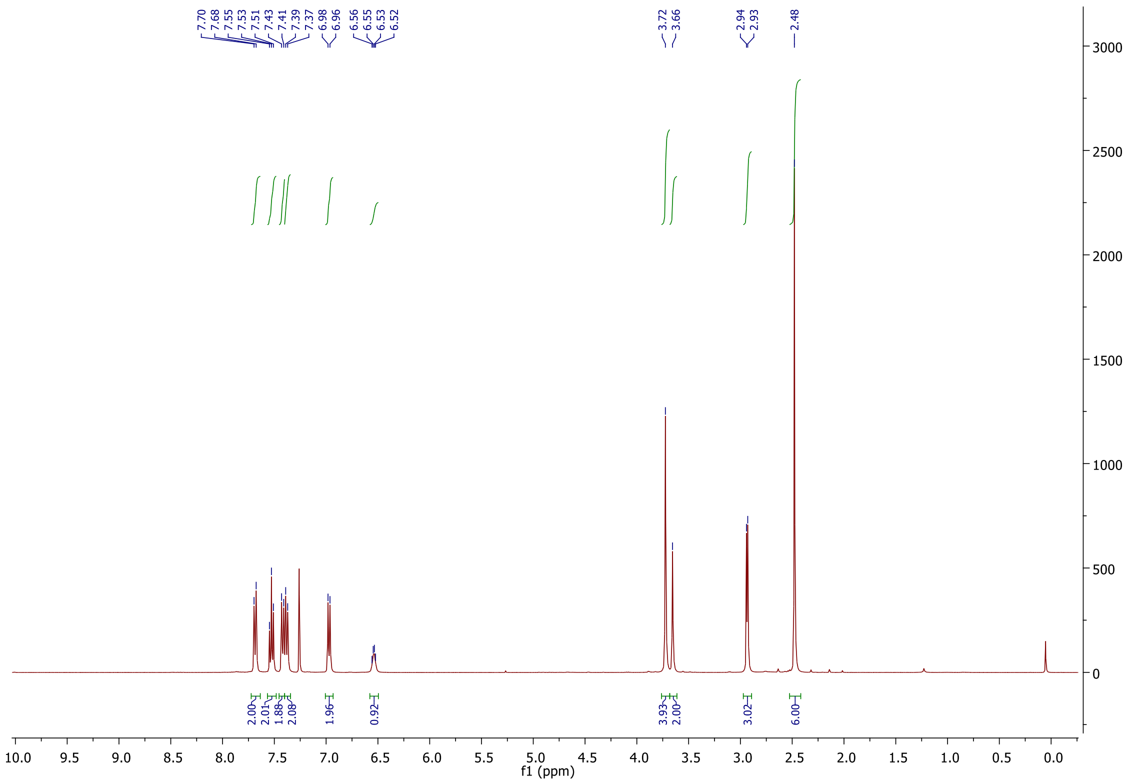

^{13}C NMR (101 MHz, $CDCl_3$)

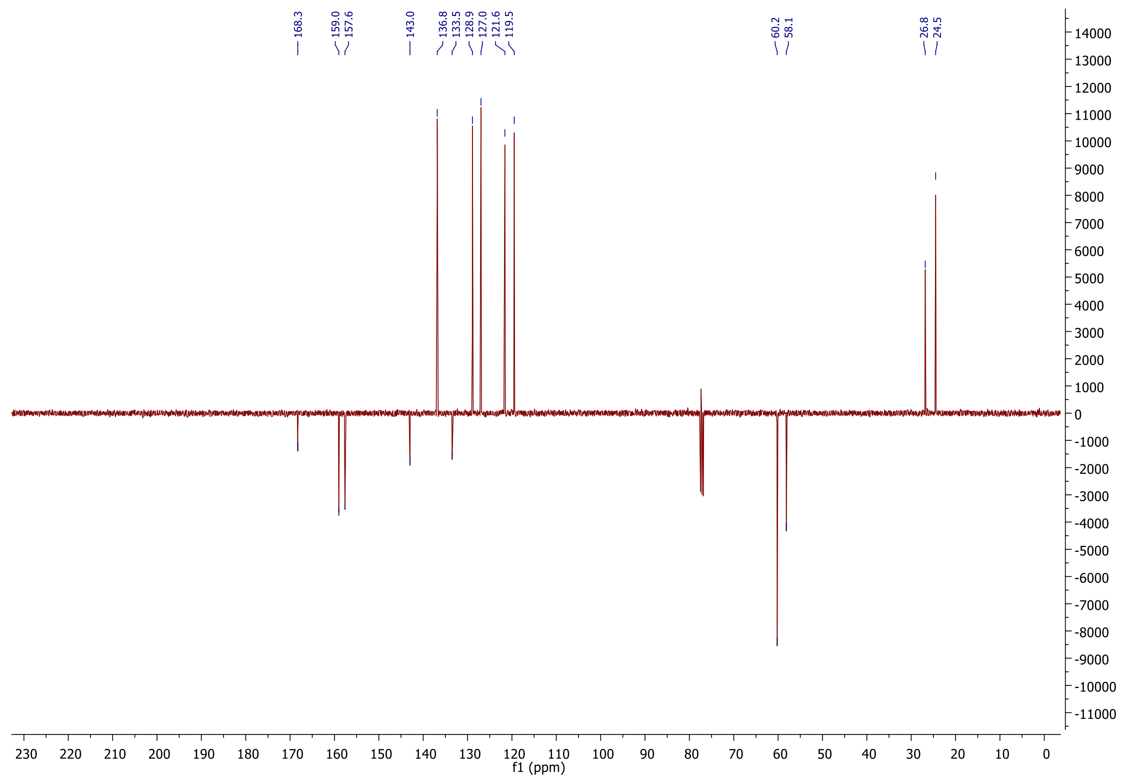

di-tert-butyl ((((4-(methylcarbamoyl)benzyl)azanediyl)bis(methylene))bis(pyridine-6,2-diyl))dicarbamate

^{1}H NMR (400 MHz, $CDCl_3$)

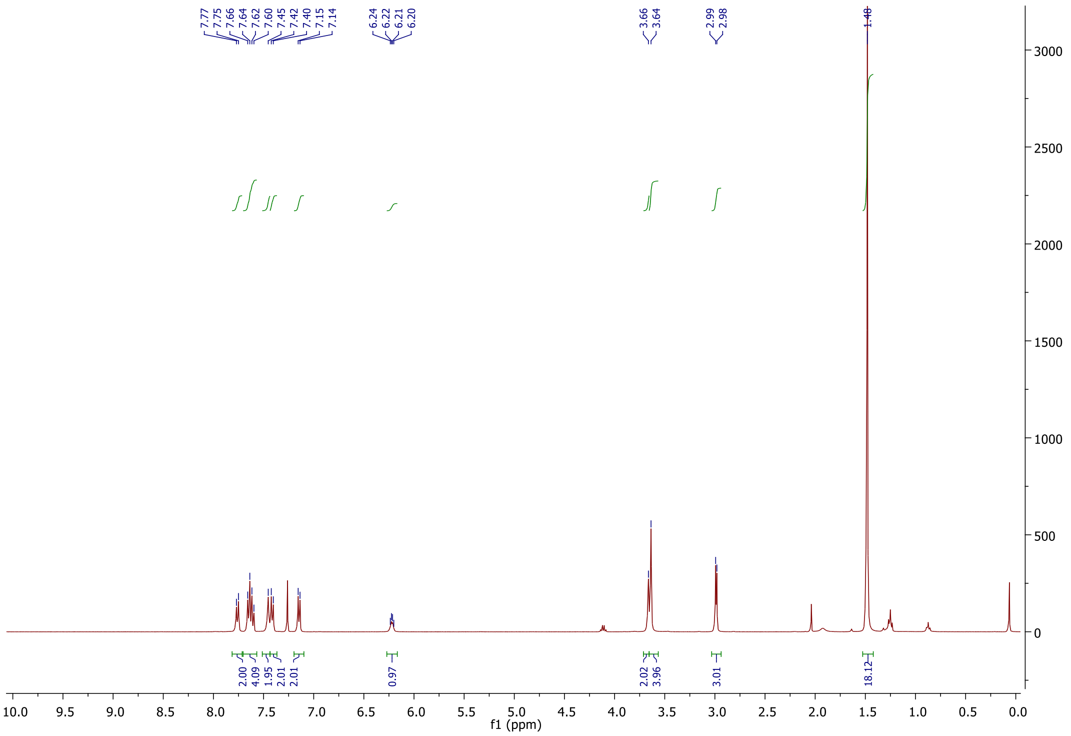

^{13}C NMR (101 MHz, $CDCl_3$)

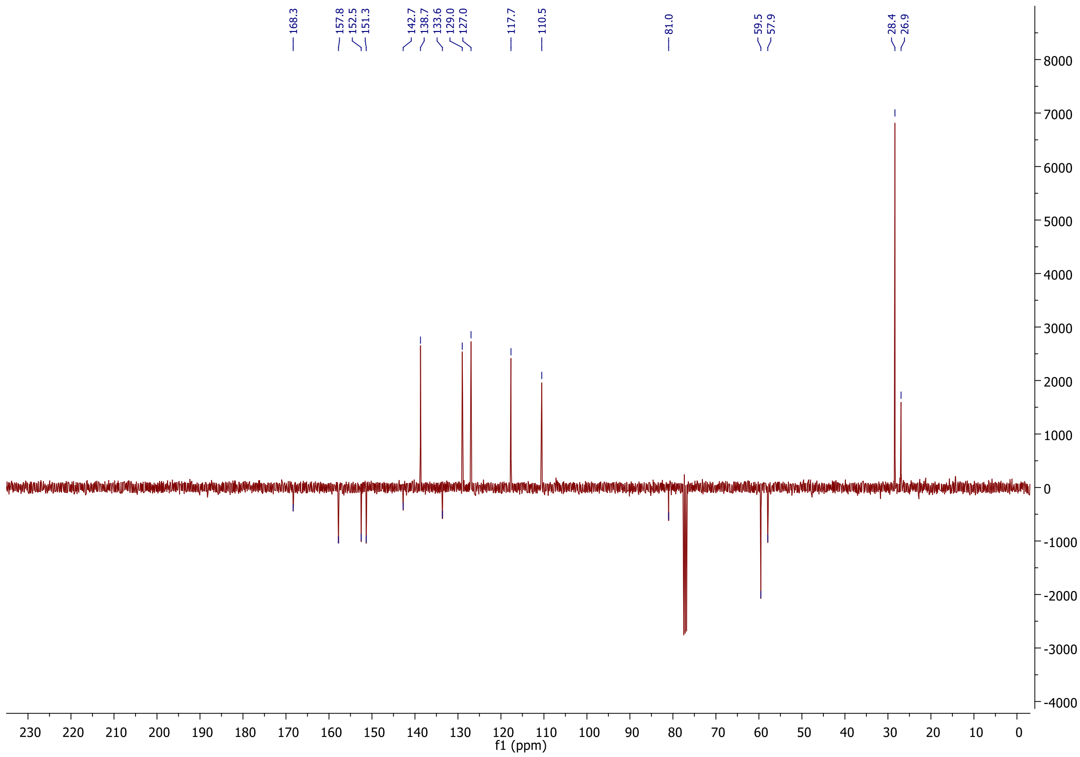

4-((bis((6-aminopyridin-2-yl)methyl)amino)methyl)-N-methylbenzamide **14**

^{1}H NMR (400 MHz, MeOD-d4)

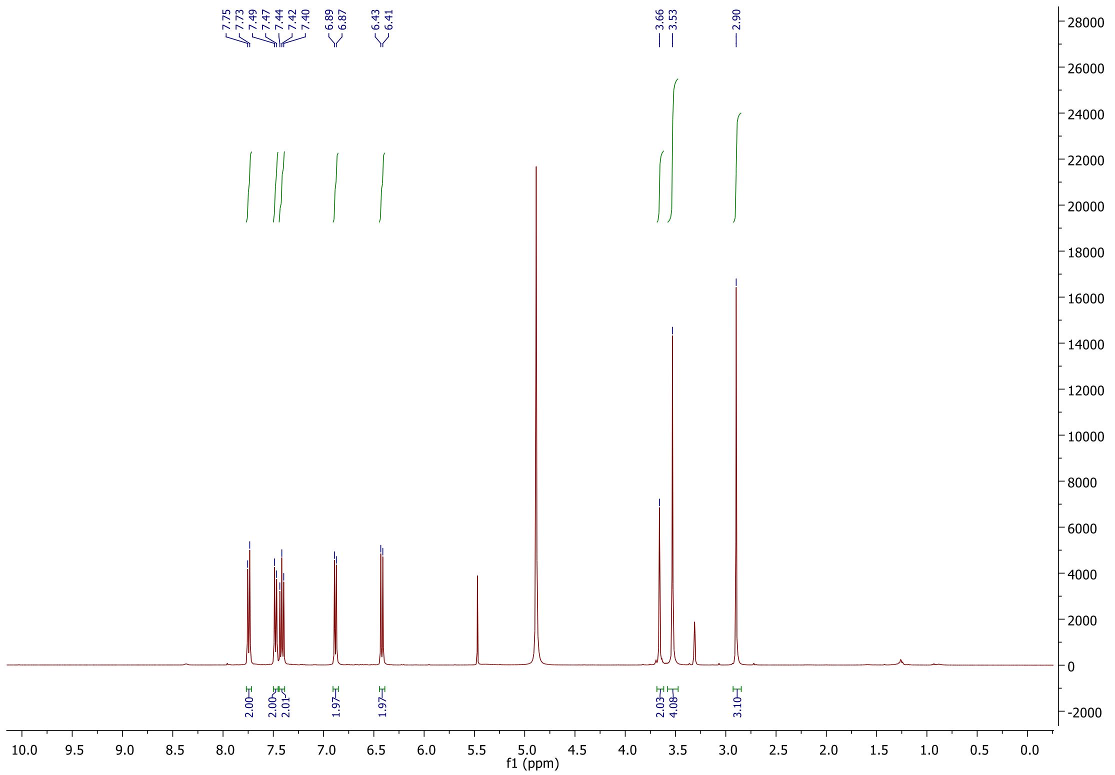

^{13}C NMR (101 MHz, MeOD-d4)

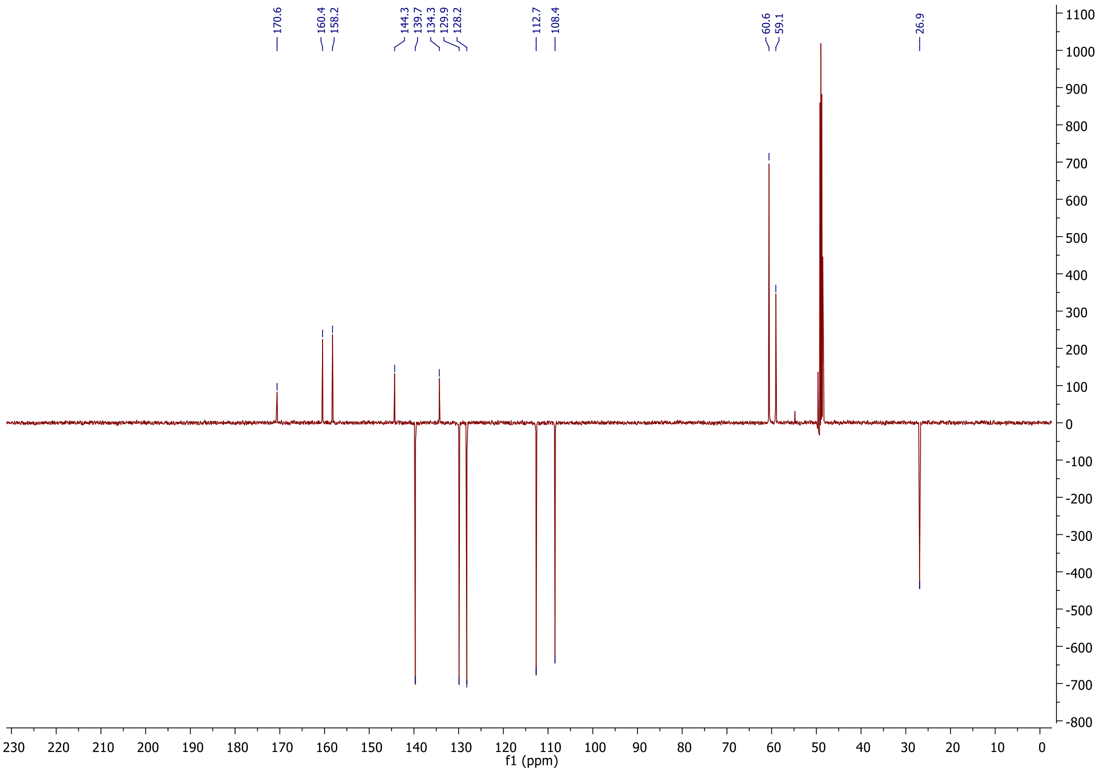

Metal complex formation

4-((bis(pyridin-2-ylmethyl)amino)methyl)-N-methylbenzamide **12** ·$ZnCl_2$ Complex

The ligand (1eq) was taken up in a small vial and a small amount of methanol was added to dissolve the ligand. $ZnCl_2$ (1.1eq) was weight out in a small vial and dissolved in a small amount of methanol. This solution was slowly added to the first solution. The methanolic mixture was placed in a closed system containing diethyl ether and left to crystallise (diffusion crystallisation).The white crystals were isolated and washed with Et_2O giving crystals suitable for X-ray studies.

4-((bis((6-methylpyridin-2-yl)methyl)amino)methyl)-N-methylbenzamide **13** ·$ZnCl_2$ Complex

The ligand (1 eq) was taken up in a small vial and a small amount of methanol was added to dissolve the ligand. $ZnCl_2$ (1.1 eq) was weight out in a small vial and dissolved in a small amount of methanol. This solution was slowly added to the first solution giving a white crystalline solid within seconds after addition. The solid was isolated and washed with Et_2O giving crystals suitable for X-ray studies.

4-((bis((6-aminopyridin-2-yl)methyl)amino)methyl)-N-methylbenzamide **14** ·$ZnCl_2$ Complex

The ligand (1 eq) was taken up in a small vial and a small amount of methanol was added to dissolve the ligand. $ZnCl_2$ (1.1 eq) was weight out in a small vial and dissolved in a small amount of methanol. This solution was slowly added to the first solution. The methanolic mixture was placed in a closed system containing diethyl ether and left to crystallise (diffusion crystallisation).The slightly yellow crystals were isolated and washed with Et_2O giving crystals suitable for X-ray studies.

Synthesis of PNA conjugates

PNAzyme 3 (PNA-ligand-NH2)

The Resin-PNA (5.7 mg; 1 eq) was placed in a reactor containing a bottom filter, and the MTT group was removed by repetitive washes of the resin with TFA/DCM (1:99) solution. The wash was continued for approximately 1min until a clear and colourless cleaving phase was observed (bright yellow becoming colourless). The resin was then washed with DCM, NMP and NMP+NMM followed by NMP.

The ligand (9.5 mg; 25 eq) and HATU (5.9 mg; 23 eq) were taken up in NMP (80 ul) and DIPEA (5.4 ul; 46 eq) was added. The pre-activation was left to react for 1h at RT. The preactivated solution was added to the washed and deprotected resin and left to react for 1.5h at RT. The resin was then washed with NMP (10x2 ml) and DCM (10x2 ml).

The PNA conjugate was fully deprotected and cleaved off the solid support by use of a cleavage mixture containing TFA/H_2O/TIS (95:2.5:2.5) (1 ml) for 90 min at RT. The cleavage mixture was collected and evaporated *in vacuo* over N_2 stream. Water (1.5 ml) was added and the water phase was washed with Et_2O (2x1.5 ml). The water

phase was then evaporated *in vacuo* before taken up in MilliQ water and purified by RP-HPLC.

MALDI: m/z $[M+H]^+$ calcd for $[C_{150}H_{188}N_{74}O_{38} + H^+]$: 3635.5160; Found: 3637.4154

PNAzyme 2 (PNA-ligand-Me)

The Resin-PNA (5.8mg; 1eq) was placed in a reactor containing a bottom filter, and the MTT group was removed by repetitive washes of the resin with TFA/DCM (1:99) solution. The wash was continued for approximately 1min until a clear and colourless cleaving phase was observed (bright yellow becoming colourless). The resin was then washed with DCM, NMP and NMP+NMM followed by NMP.

The ligand (6.3mg; 25eq) and HATU (5.9mg; 23eq) were taken up in NMP (80ul) and DIPEA (5.4ul; 46eq) was added. The pre-activation was left to react for 1h at RT. The preactivated solution was added to the washed and deprotected resin and left to react for 1.5h at RT. The resin was then washed with NMP (10x2ml) and DCM (10x2ml).

The PNA conjugate was fully deprotected and cleaved off the solid support by use of a cleavage mixture containing TFA/H_2O/TIS (95:2.5:2.5) (1ml) for 90min at RT. The cleavage mixture was collected and evaporated *in vacuo* over N_2 stream. Water (1.5ml) was added and the water phase was washed with Et_2O (2x1.5ml). The water phase was then evaporated *in vacuo* before taken up in MilliQ water and purified by RP-HPLC.

MALDI: m/z $[M+H]^+$ calcd for $[C_{152}H_{190}N_{72}O_{38} + H^+]$: 3633.5255; Found: 3634.2500

PNAzyme 1 (PNA-ligand-H):

The Resin-PNA (5.6 mg; 1eq) was placed in a reactor containing a bottom filter, and the MTT group was removed by repetitive washes of the resin with TFA/DCM (1:99) solution. The wash was continued for approximately 1min until a clear and colourless cleaving phase was observed (bright yellow becoming colourless). The resin was then washed with DCM, NMP and NMP+NMM followed by NMP.

The ligand (5.6 mg; 25eq) and HATU (5.9 mg; 23eq) were taken up in NMP (80 ul) and DIPEA (5.4 ul; 46eq) was added. The pre-activation was left to react for 1h at RT. The preactivated solution was added to the washed and deprotected resin and left to react for 1.5h at RT. The resin was then washed with NMP (10x2 ml) and DCM (10x2 ml).

The PNA conjugate was fully deprotected and cleaved off the solid support by use of a cleavage mixture containing TFA/H_2O/TIS (95:2.5:2.5) (1 ml) for 90 min at RT. The cleavage mixture was collected and evaporated *in vacuo* over N_2 stream. Water (1.5 ml) was added and the water phase was washed with Et_2O (2x1.5 ml). The water phase was then evaporated *in vacuo* before taken up in MilliQ water and purified by RP-HPLC.

MALDI: m/z $[M+H]^+$ calcd for $[C_{150}H_{186}N_{72}O_{38} + H^+]$: 3605.4942; Found: 3606.2969

The PNAzymes were purified on Ascentis Express Supelco Peptide ES-C18 column (2.7 µm, 150 × 4.6 mm) with a linear gradient elution of 0% to 40% buffer B over 30 min at 60 °C, using a flow rate of 1.0 mL/min and UV detection at 260 nm. Solvent system used: solvent A: 0.1% TFA in water; solvent B: 50% MeCN: water containing 0.1% TFA. Collected products were evaporated to dryness and lyophilised from water (×3). The final products were analysed by MALDI-MS in positive ion-mode using a sinapic acid matrix (10 mg/mL in 0.1% TFA/milliQ and MeCN (2:1, v/v)

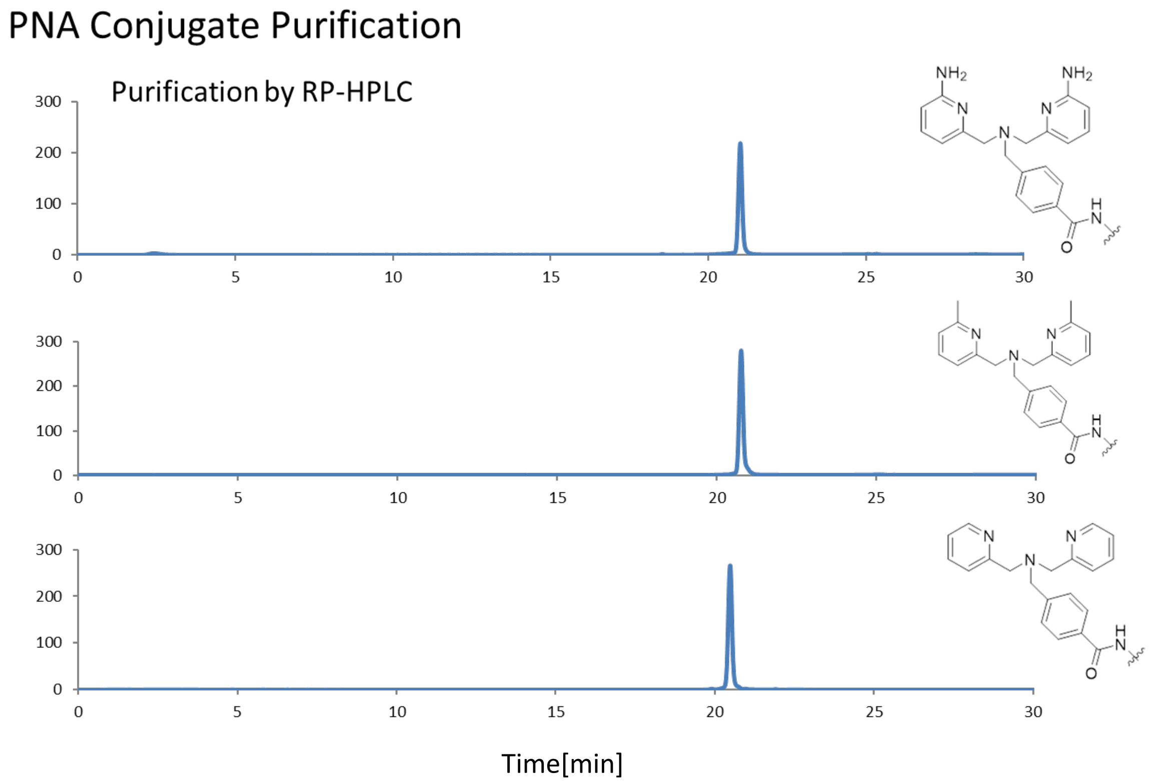

MALDI of PNA conjugates

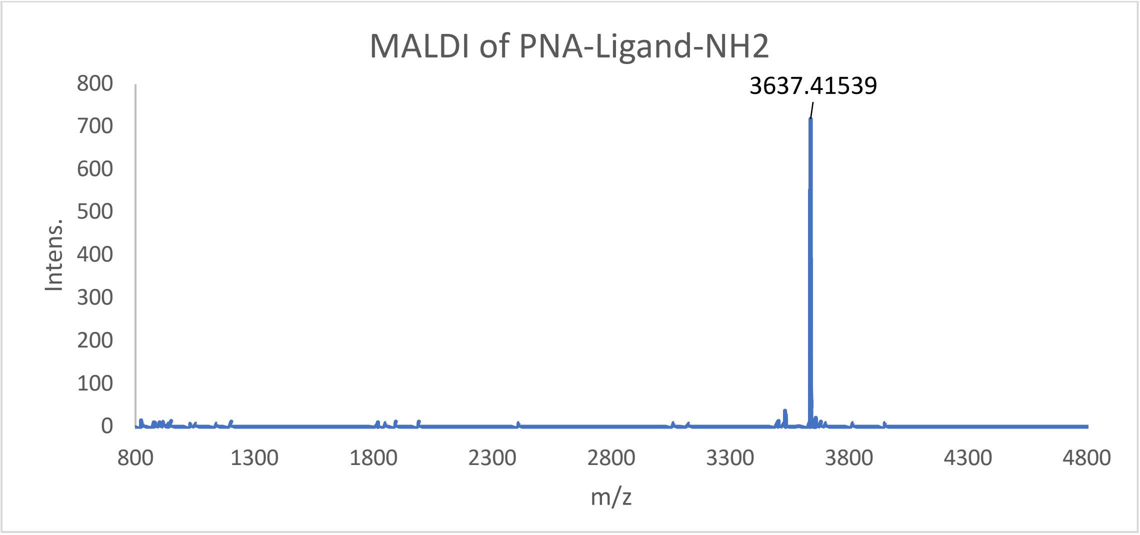

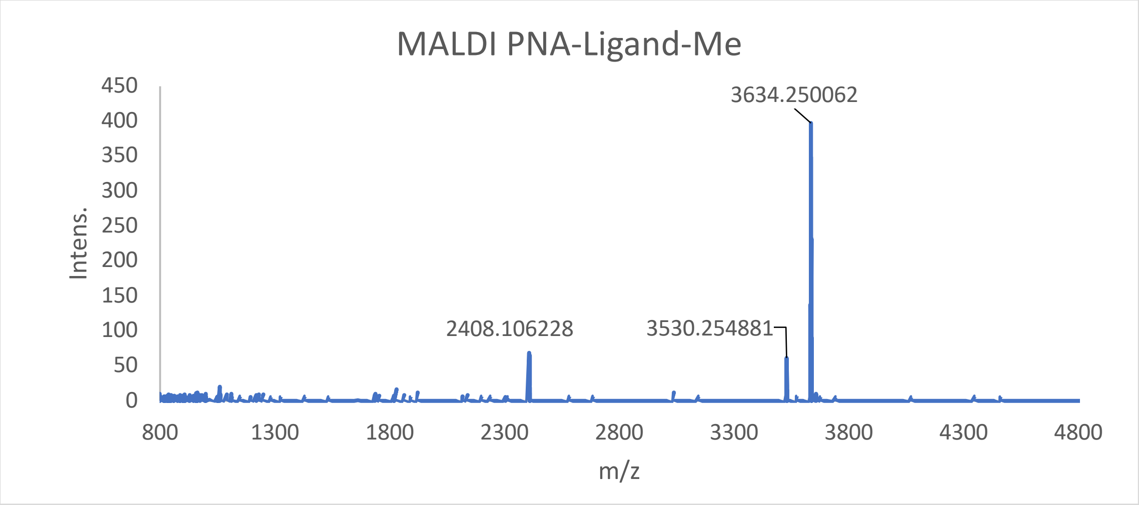

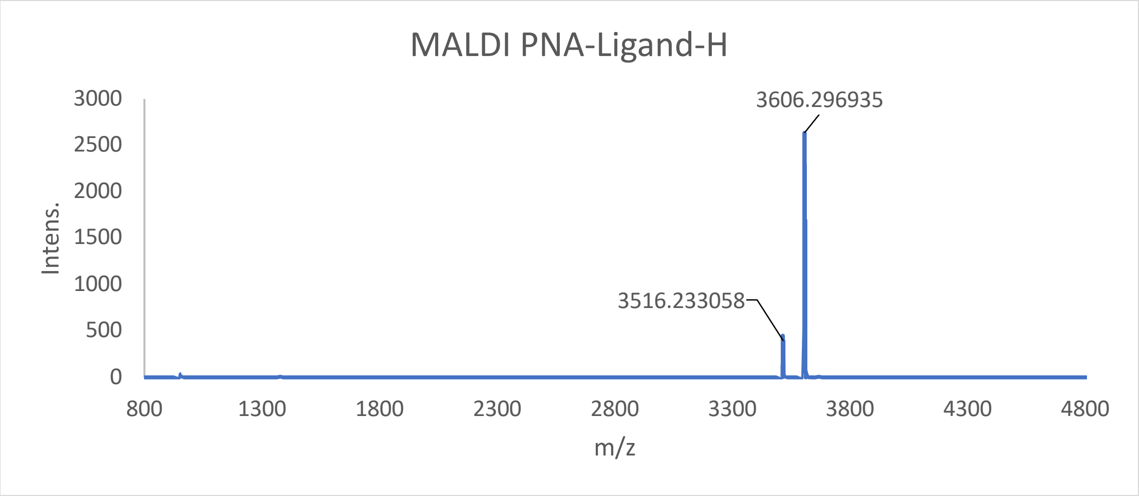

Titration experiments

For the titration experiments, the following standard conditions were used for all experiments. All solutions used were freshly made from chemicals of analytical grade and miliQwater.

For titration of the ligand itself, the ligand (1eq; 0.02mmol) was taken up in NaCl (0.10M) (10000µl). HCl (1M) (40µl; 2eq) was added and the solution was placed in a water bath at 25°C. The solution was titrated with NaOH (0.2M) (10µl pr. Addition; 1/10 eq.). The stirring was turned on after each addition and the pH was allowed to stabilize before each measurement without stirring.

For titration of the ligand in presence of metal ion, the ligand (1eq; 0.02mmol) was taken up in NaCl (0.10M) + $ZnCl_2$ (2.0mM; 1eq) (10000µl). HCl (1M) (40µl; 2eq) was added and the solution was placed in a water bath at 25°C. The solution was titrated with NaOH (0.2M) (10µl pr. Addition; 1/10 eq.). The stirring was turned on after each addition and the pH was allowed to stabilize before each measurement without stirring.

Speciation Curves

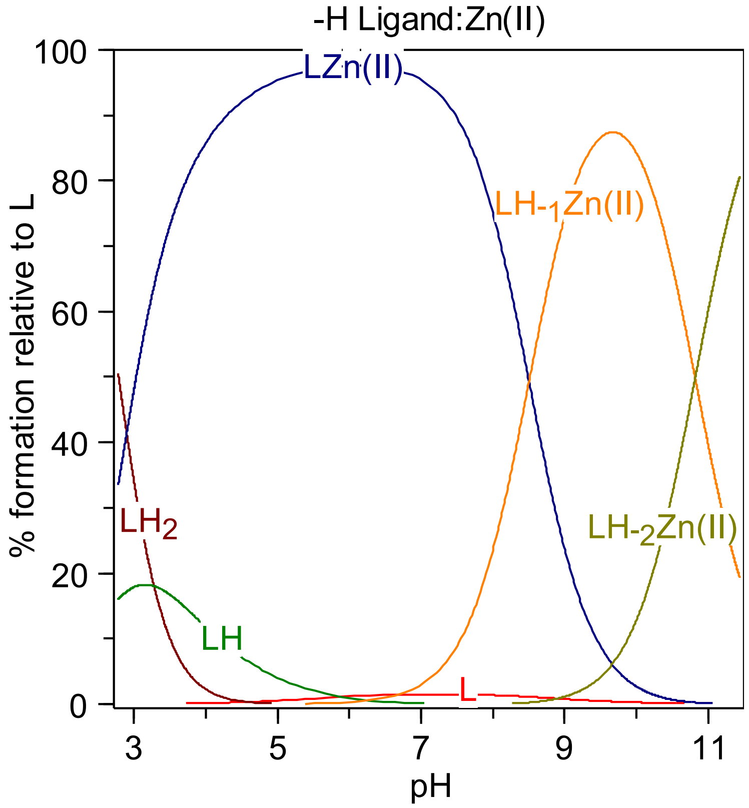

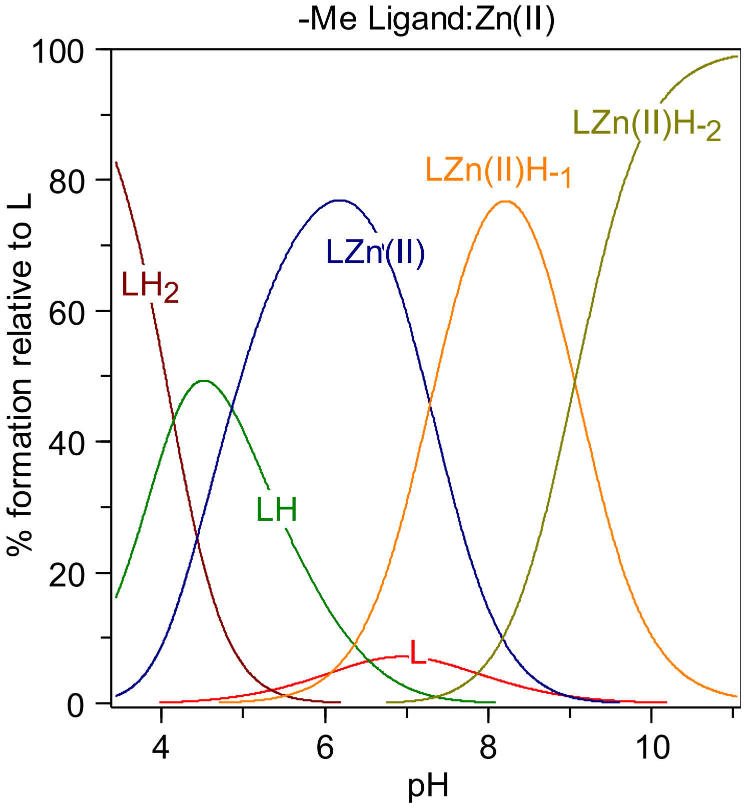

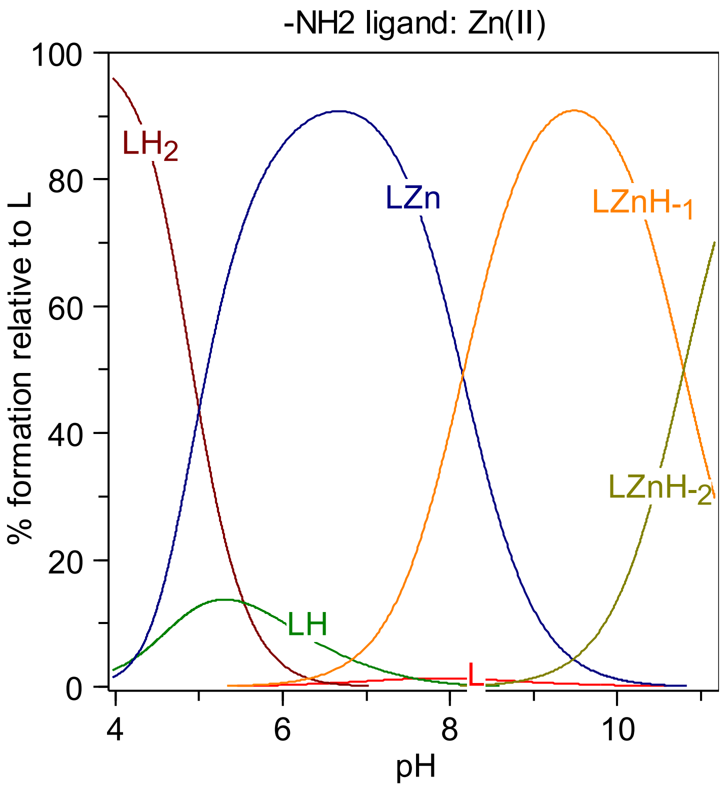

The reaction of HPNPP

Stock solutions of HPNPP (5.0 mM), HEPES Buffer (0.1 M), Zn(II)Cl_2 or Zn$(NO)_2$ (50 mM) and the respective ligand (50 mM, DMSO) were prepared. Reaction mixtures were prepared directly in the cuvette by adding ligand, Zn(II), buffer and water giving a total volume of 2 ml and final concentrations of [HPNPP]= 0.05 mM and [HEPES]= 50 mM. The cuvettes were placed in the UV cell block to equilibrate. HPNPP was added as the last component and the complete reaction was followed at 400nm. For the faster reactions, the integrated first order rate equation was fitted to the increase in absorbance with time to obtain the observed first rate constant (kobs) for each reaction. For slower reactions, initial rate experiments followed the same procedure but with use of HPNPP stock- solution of 50mM and final concentrations of 5.0 mM. 2-5% of the reaction was followed. Data was fitted to straight lines and divided by the extinction coefficient ε = 9575 at pH 7.0.

The reaction of PNAzymes

RNA cleavage reactions were carried out in sealed tubes immersed in a thermostated water bath (37 °C). Experiments were performed at pH 7.0 in HEPES buffer (10 mM HEPES, 0.1 M NaCl). RNA targets (4 µM final concentration, 1eq) were equilibrated in appropriate amounts of water and HEPES buffer over a 15-minute period at 37 °C prior to the addition of Zn(II) solution (100 µM final concentration) and 1.3 eq. of the PNAzyme. The reaction mixtures were then allowed to incubate at 37 °C. Aliquots (40 µL) were withdrawn at 3h, 6h, 24h, 48h, 72h and 96h time points and immediately quenched with 70 µL of 1.0 mM EDTA in 30% MeCN/milliQ. The samples were analysed by anion exchange HPLC (IE-HPLC) using a Dionex NucleoPac PA-100 (4 × 250 mm) column with a linear gradient elution of 0–45% buffer B over 30 min at 60

◦C. A flow rate of 1.5 mL/min was used and UV detection was carried out at 260 nm. The following solvent system was used: (A) 20 mM NaOAc in 30% aq. MeCN; and (B) 20 mM NaOAc, 0.4 M $LiClO_4$ in 30% aq. MeCN. Cleavage of RNA substrates was obtained by quantification of the remaining RNA and the sum of the formed fragments detected in the IE-HPLC analysis.

ONW144s_(12.Zn)

Table S1 Crystal data and structure refinement for ONW144s_.

Identification code	ONW144s_
Empirical formula	$C_{21}H_{22}Cl_2N_4OZn$
Formula weight	482.69
Temperature/K	100
Crystal system	orthorhombic
Space group	Pbca
a/Å	12.5333(9)
b/Å	17.5056(12)
c/Å	19.2519(14)
α/°	90
β/°	90
γ/°	90
Volume/Å^3	4223.9(5)
Z	8
ρ_{calc}g/cm^3	1.518
μ/mm^{-1}	1.436
F(000)	1984.0
Crystal size/mm^3	0.5 × 0.3 × 0.3
Radiation	MoKα (λ = 0.71073)
2Θ range for data collection/°	4.232 to 57.212
Index ranges	-16 ≤ h ≤ 16, -20 ≤ k ≤ 23, -24 ≤ l ≤ 25
Reflections collected	69597
Independent reflections	5392 [R_{int} = 0.0277, R_{sigma} = 0.0160]
Data/restraints/parameters	5392/1/267
Goodness-of-fit on F^2	1.073
Final R indexes [I>=2σ (I)]	R_1 = 0.0271, wR_2 = 0.0643
Final R indexes [all data]	R_1 = 0.0371, wR_2 = 0.0690
Largest diff. peak/hole / e Å^{-3}	0.42/-0.45

Table S2 Fractional Atomic Coordinates (×10^4) and Equivalent Isotropic Displacement Parameters (Å^2×10^3) for ONW144s_. U_{eq} is defined as 1/3 of of the trace of the orthogonalised U_{IJ} tensor.

Atom	*x*	*y*	*z*	U(eq)
Zn1	1629.3(2)	2523.8(2)	6395.7(2)	14.20(6)
Cl1	1264.2(3)	1825.3(2)	7354.8(2)	22.36(9)
Cl2	467.7(3)	2451.6(2)	5485.5(2)	17.93(8)
O1	7845.3(9)	5539.5(7)	6008.8(7)	23.1(3)

N1	2860.9(10)	1798.9(7)	5956.7(7)	14.9(3)
N2	1128.5(11)	3662.8(8)	6706.2(7)	18.7(3)
N3	3133.9(10)	3132.5(7)	6597.2(7)	14.8(3)
N4	6650.2(11)	6501.5(8)	5928.3(8)	19.2(3)
C1	2733.1(13)	1259.7(9)	5468.9(8)	16.9(3)
C2	3566.9(13)	809.9(9)	5230.4(9)	19.3(3)
C3	4575.2(13)	927.2(9)	5510.9(9)	19.9(3)
C4	4716.1(13)	1482.5(9)	6017.3(8)	17.5(3)
C5	3841.7(12)	1908.6(9)	6227.2(8)	14.6(3)
C6	175.0(14)	3991.9(10)	6583.9(9)	22.7(3)
C7	-81.2(14)	4715.9(10)	6817.1(9)	24.3(4)
C8	679.8(15)	5122.3(10)	7187.9(9)	24.5(4)
C9	1671.8(15)	4797.0(9)	7304.2(9)	21.9(3)
C10	1865.5(13)	4061.1(9)	7058.7(8)	17.8(3)
C11	3909.3(12)	2528.9(9)	6773.4(8)	16.3(3)
C12	2909.9(13)	3648.7(9)	7183.6(8)	17.7(3)
C13	3426.7(13)	3552.9(9)	5945.9(8)	17.0(3)
C14	4339.5(13)	4115.1(9)	6002.1(8)	16.2(3)
C15	5405.4(13)	3888.3(9)	5944.6(8)	18.3(3)
C16	6218.6(13)	4426.2(9)	5959.5(8)	17.8(3)
C17	5992.7(12)	5203.6(9)	6013.5(8)	15.5(3)
C18	4931.5(13)	5430.0(9)	6089.0(9)	21.4(3)
C19	4120.1(13)	4889.5(10)	6088.2(9)	21.9(4)
C20	6907.0(13)	5761.6(9)	5985.4(8)	16.4(3)
C21	7445.3(14)	7100.6(9)	5881.2(9)	22.5(3)

Table S3 Anisotropic Displacement Parameters ($Å^2×10^3$) for ONW144s_. The Anisotropic displacement factor exponent takes the form: $-2\pi^2[h^2a^{*2}U_{11}+2hka^*b^*U_{12}+…]$.

Atom	U_{11}	U_{22}	U_{33}	U_{23}	U_{13}	U_{12}
Zn1	11.65(9)	14.90(9)	16.06(10)	0.47(7)	0.43(6)	-0.78(7)
Cl1	25.4(2)	23.93(19)	17.71(19)	3.45(14)	4.31(16)	-1.89(16)
Cl2	14.18(17)	20.83(18)	18.78(18)	2.30(14)	-2.08(14)	-0.70(14)
O1	12.6(6)	22.9(6)	33.9(7)	-0.6(5)	0.6(5)	1.2(5)
N1	12.8(6)	15.5(6)	16.3(6)	0.5(5)	0.0(5)	-0.1(5)
N2	17.0(7)	17.8(6)	21.4(7)	1.4(5)	1.3(5)	1.2(5)
N3	15.1(6)	15.0(6)	14.3(6)	0.1(5)	-1.1(5)	0.1(5)
N4	10.6(7)	18.7(7)	28.2(8)	4.8(6)	-1.1(6)	-0.7(5)
C1	14.9(8)	17.4(7)	18.5(8)	0.8(6)	-1.2(6)	-1.5(6)
C2	20.6(8)	17.6(7)	19.6(8)	-1.3(6)	0.0(6)	1.3(6)
C3	17.6(8)	19.6(8)	22.6(8)	1.8(6)	2.1(6)	6.0(6)
C4	13.3(7)	19.8(8)	19.5(8)	3.4(6)	-0.4(6)	0.9(6)
C5	13.4(7)	14.7(7)	15.6(7)	2.8(5)	0.2(6)	-1.4(6)
C6	17.1(8)	22.7(8)	28.2(9)	3.7(7)	2.1(7)	0.8(7)

C7	22.1(9)	24.0(8)	26.8(9)	8.6(7)	7.0(7)	6.8(7)
C8	34.1(10)	18.5(8)	21.1(8)	2.1(6)	6.6(7)	6.2(7)
C9	30.6(9)	17.3(8)	17.8(8)	-0.8(6)	0.8(7)	0.2(7)
C10	20.0(8)	17.3(7)	16.2(8)	1.4(6)	2.1(6)	0.3(6)
C11	14.5(7)	18.2(7)	16.3(7)	1.1(6)	-2.4(6)	0.9(6)
C12	19.4(8)	17.6(7)	16.1(7)	-1.7(6)	-2.1(6)	-0.5(6)
C13	17.0(8)	17.5(7)	16.6(7)	2.6(6)	-2.3(6)	-2.9(6)
C14	16.0(8)	17.3(7)	15.5(7)	2.6(6)	-0.9(6)	-2.0(6)
C15	19.6(8)	15.4(7)	19.9(8)	-2.6(6)	4.2(6)	1.8(6)
C16	14.4(7)	20.6(8)	18.4(8)	-1.2(6)	3.5(6)	2.8(6)
C17	14.9(7)	16.9(7)	14.6(7)	1.7(6)	0.5(6)	-0.4(6)
C18	16.5(8)	14.5(7)	33.3(10)	4.4(6)	3.5(7)	1.8(6)
C19	11.3(7)	19.4(8)	35.1(10)	7.1(7)	1.3(7)	2.3(6)
C20	14.9(7)	20.0(8)	14.4(7)	-0.4(6)	1.1(6)	0.5(6)
C21	16.9(8)	20.2(8)	30.5(9)	4.6(7)	-2.3(7)	-4.6(7)

Table S4 Bond Lengths for ONW144s_.

Atom	Atom	Length/Å	Atom	Atom	Length/Å
Zn1	Cl1	2.2614(4)	C3	C4	1.388(2)
Zn1	Cl2	2.2817(4)	C4	C5	1.386(2)
Zn1	N1	2.1697(13)	C5	C11	1.514(2)
Zn1	N2	2.1741(14)	C6	C7	1.382(2)
Zn1	N3	2.2005(13)	C7	C8	1.388(3)
O1	C20	1.240(2)	C8	C9	1.386(3)
N1	C1	1.341(2)	C9	C10	1.393(2)
N1	C5	1.349(2)	C10	C12	1.514(2)
N2	C6	1.347(2)	C13	C14	1.513(2)
N2	C10	1.342(2)	C14	C15	1.398(2)
N3	C11	1.475(2)	C14	C19	1.393(2)
N3	C12	1.473(2)	C15	C16	1.388(2)
N3	C13	1.499(2)	C16	C17	1.394(2)
N4	C20	1.339(2)	C17	C18	1.395(2)
N4	C21	1.450(2)	C17	C20	1.507(2)
C1	C2	1.387(2)	C18	C19	1.389(2)
C2	C3	1.390(2)			

Table S5 Bond Angles for ONW144s_.

Atom	Atom	Atom	Angle/°	Atom	Atom	Atom	Angle/°
Cl1	Zn1	Cl2	117.906(17)	N1	C5	C4	122.11(14)
N1	Zn1	Cl1	98.38(4)	N1	C5	C11	114.92(13)
N1	Zn1	Cl2	97.03(4)	C4	C5	C11	122.97(14)

N1	Zn1	N2	148.09(5)	N2	C6	C7	122.77(17)
N1	Zn1	N3	75.06(5)	C6	C7	C8	118.52(17)
N2	Zn1	Cl1	102.30(4)	C9	C8	C7	119.29(16)
N2	Zn1	Cl2	94.46(4)	C8	C9	C10	118.78(16)
N2	Zn1	N3	75.81(5)	N2	C10	C9	122.14(16)
N3	Zn1	Cl1	106.94(4)	N2	C10	C12	115.33(14)
N3	Zn1	Cl2	135.15(4)	C9	C10	C12	122.52(15)
C1	N1	Zn1	126.83(11)	N3	C11	C5	108.49(12)
C1	N1	C5	118.65(13)	N3	C12	C10	109.61(13)
C5	N1	Zn1	114.50(10)	N3	C13	C14	116.39(13)
C6	N2	Zn1	126.88(12)	C15	C14	C13	122.16(14)
C10	N2	Zn1	114.64(11)	C19	C14	C13	119.48(15)
C10	N2	C6	118.48(15)	C19	C14	C15	118.32(15)
C11	N3	Zn1	104.96(9)	C16	C15	C14	120.50(15)
C11	N3	C13	112.50(12)	C15	C16	C17	120.98(15)
C12	N3	Zn1	105.59(9)	C16	C17	C18	118.60(15)
C12	N3	C11	112.88(12)	C16	C17	C20	118.41(14)
C12	N3	C13	112.73(12)	C18	C17	C20	122.99(14)
C13	N3	Zn1	107.46(9)	C19	C18	C17	120.28(15)
C20	N4	C21	122.66(14)	C18	C19	C14	121.23(15)
N1	C1	C2	122.79(15)	O1	C20	N4	122.31(15)
C1	C2	C3	118.21(15)	O1	C20	C17	121.13(14)
C4	C3	C2	119.49(15)	N4	C20	C17	116.56(14)
C5	C4	C3	118.76(15)				

Table S6 Hydrogen Bonds for ONW144s_.

D	H	A	d(D-H)/Å	d(H-A)/Å	d(D-A)/Å	D-H-A/°
N4	H4	Cl2[1]	0.776(15)	2.520(16)	3.2464(15)	156(2)
C7	H7	O1[2]	0.95	2.47	3.354(2)	155.6

[1]1/2-X,1/2+Y,+Z; [2]-1+X,+Y,+Z

Table S7 Torsion Angles for ONW144s_.

A	B	C	D	Angle/°	A	B	C	D	Angle/°
Zn1	N1	C1	C2	-178.45(12)	C8	C9	C10	N2	0.9(2)
Zn1	N1	C5	C4	178.56(12)	C8	C9	C10	C12	-177.73(15)
Zn1	N1	C5	C11	-1.42(16)	C9	C10	C12	N3	-148.85(15)
Zn1	N2	C6	C7	178.41(12)	C10	N2	C6	C7	-1.2(2)
Zn1	N2	C10	C9	-179.19(12)	C11	N3	C12	C10	-160.31(13)

Zn1	N2	C10	C12	-0.45(17)	C11	N3	C13	C14	-74.67(17)
Zn1	N3	C11	C5	49.12(13)	C12	N3	C11	C5	163.62(13)
Zn1	N3	C12	C10	-46.19(13)	C12	N3	C13	C14	54.36(18)
Zn1	N3	C13	C14	170.29(11)	C13	N3	C11	C5	-67.42(16)
N1	C1	C2	C3	-0.1(2)	C13	N3	C12	C10	70.85(16)
N1	C5	C11	N3	-33.07(18)	C13	C14	C15	C16	176.32(15)
N2	C6	C7	C8	0.5(3)	C13	C14	C19	C18	-175.05(16)
N2	C10	C12	N3	32.42(19)	C14	C15	C16	C17	-1.7(2)
N3	C13	C14	C15	84.25(19)	C15	C14	C19	C18	2.6(3)
N3	C13	C14	C19	-98.18(18)	C15	C16	C17	C18	3.3(2)
C1	N1	C5	C4	-0.1(2)	C15	C16	C17	C20	-176.53(14)
C1	N1	C5	C11	179.96(13)	C16	C17	C18	C19	-2.0(2)
C1	C2	C3	C4	0.2(2)	C16	C17	C20	O1	-9.7(2)
C2	C3	C4	C5	-0.3(2)	C16	C17	C20	N4	169.78(15)
C3	C4	C5	N1	0.2(2)	C17	C18	C19	C14	-1.0(3)
C3	C4	C5	C11	-179.80(14)	C18	C17	C20	O1	170.52(16)
C4	C5	C11	N3	146.95(15)	C18	C17	C20	N4	-10.0(2)
C5	N1	C1	C2	0.0(2)	C19	C14	C15	C16	-1.3(2)
C6	N2	C10	C9	0.5(2)	C20	C17	C18	C19	177.86(15)
C6	N2	C10	C12	179.20(14)	C21	N4	C20	O1	0.7(3)
C6	C7	C8	C9	0.9(2)	C21	N4	C20	C17	-178.70(15)
C7	C8	C9	C10	-1.6(2)					

Table S8 Hydrogen Atom Coordinates ($Å\times10^4$) and Isotropic Displacement Parameters ($Å^2\times10^3$) for ONW144s_.

Atom	*x*	*y*	*z*	U(eq)
H4	6059(13)	6624(11)	5885(11)	23(5)
H1	2042	1182.59	5278.65	20
H2	3451.83	431.73	4884.46	23
H3	5163.51	629.74	5357.2	24
H4A	5399.09	1568.74	6216.37	21
H6	-343.13	3714.66	6326.66	27
H7	-763.05	4930.53	6725.43	29
H8	522.45	5618.18	7360.18	29
H9	2209.91	5070.67	7546.81	26
H11A	3744.59	2314.08	7236.84	20
H11B	4639.47	2744.15	6786.09	20
H12A	3495	4024.94	7231.3	21
H12B	2866.11	3350.97	7619.91	21

H13A	2788.33	3832.73	5781.64	20
H13B	3610.97	3171.69	5585.55	20
H15	5574.33	3362.12	5895.06	22
H16	6939.92	4262.13	5932.44	21
H18	4763.28	5956.06	6141.19	26
H19	3402.13	5050.89	6147.56	26
H21A	7957.46	6975.15	5513.83	34
H21B	7821.11	7145.09	6325.76	34
H21C	7095.01	7586.48	5772.17	34

Crystal structure determination of ONW144s_

Crystal Data for $C_{21}H_{22}Cl_2N_4OZn$ (M =482.69 g/mol): orthorhombic, space group Pbca (no. 61), a = 12.5333(9) Å, b = 17.5056(12) Å, c = 19.2519(14) Å, V = 4223.9(5) Å^3, Z = 8, T = 100 K, μ(MoKα) = 1.436 mm^{-1}, $Dcalc$ = 1.518 g/cm^3, 69597 reflections measured (4.232° ≤ 2Θ ≤ 57.212°), 5392 unique (R_{int} = 0.0277, R_{sigma} = 0.0160) which were used in all calculations. The final R_1 was 0.0271 (I > 2σ(I)) and wR_2 was 0.0690 (all data).

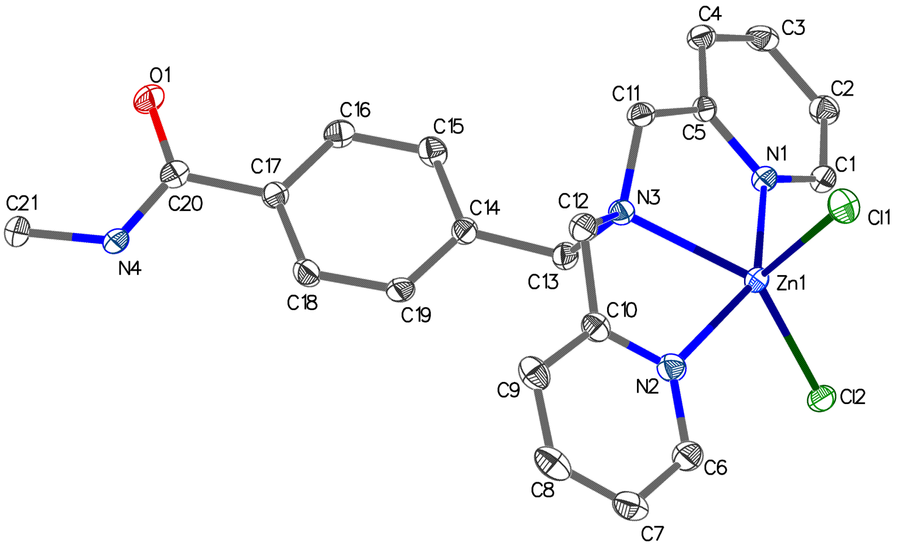

ONW143s_0m (13.Zn)

Table S9 Crystal data and structure refinement for ONW143s_0m.

Identification code	ONW143s_0m
Empirical formula	$C_{23}H_{26}Cl_2N_4OZn$
Formula weight	510.75
Temperature/K	100.0
Crystal system	monoclinic
Space group	$P2_1/n$
a/Å	11.531(5)
b/Å	9.136(4)
c/Å	22.280(10)
α/°	90
β/°	93.892(10)
γ/°	90
Volume/Å^3	2341.6(18)

Z	4
ρ_{calc}g/cm^3	1.449
μ/mm^{-1}	1.300
F(000)	1056.0
Crystal size/mm^3	0.54 × 0.23 × 0.22
Radiation	MoKα (λ = 0.71073)
2Θ range for data collection/°	3.664 to 57.276
Index ranges	-15 ≤ h ≤ 15, -12 ≤ k ≤ 10, -30 ≤ l ≤ 29
Reflections collected	65388
Independent reflections	5948 [R_{int} = 0.0300, R_{sigma} = 0.0174]
Data/restraints/parameters	5948/0/283
Goodness-of-fit on F^2	1.045
Final R indexes [I>=2σ (I)]	R_1 = 0.0244, wR_2 = 0.0593
Final R indexes [all data]	R_1 = 0.0286, wR_2 = 0.0614
Largest diff. peak/hole / e Å^{-3}	0.39/-0.29

Table S10 Fractional Atomic Coordinates (×10^4) and Equivalent Isotropic Displacement Parameters (Å^2×10^3) for ONW143s_0m. U_{eq} is defined as 1/3 of of the trace of the orthogonalised U_{IJ} tensor.

Atom	*x*	*y*	*z*	U(eq)
Zn1	2498.7(2)	8704.8(2)	5737.4(2)	11.70(5)
Cl1	3079.2(3)	10537.2(4)	5123.9(2)	17.92(7)
Cl2	884.7(3)	8109.4(4)	6199.7(2)	18.93(7)
O1	7738.0(9)	1621.6(11)	7225.8(5)	20.2(2)
N1	3516.1(10)	9573.5(12)	6554.1(5)	14.1(2)
N2	2051.5(10)	7094.5(12)	4967.1(5)	14.5(2)
N3	3827.4(9)	7074.6(12)	5888.5(5)	11.5(2)
N4	7348.8(10)	630.3(13)	6301.6(5)	16.6(2)
C1	3207.8(12)	10570.9(15)	6963.8(6)	17.4(3)
C2	3925.8(14)	10863.9(17)	7479.9(7)	22.7(3)
C3	4964.2(14)	10108.4(17)	7579.4(6)	22.7(3)
C4	5287.4(13)	9085.3(16)	7155.2(6)	18.4(3)
C5	4546.6(12)	8875.3(14)	6644.4(6)	14.1(2)
C6	2074.9(14)	11370.9(17)	6834.8(7)	25.6(3)
C7	1019.7(13)	6885.3(16)	4654.4(6)	18.5(3)
C8	779.6(14)	5583.0(17)	4339.9(7)	23.4(3)
C9	1612.1(14)	4487.1(17)	4344.3(7)	25.2(3)

C10	2685.5(13)	4717.1(16)	4657.9(7)	20.5(3)
C11	2871.4(12)	6039.9(14)	4958.4(6)	14.2(3)
C12	140.5(14)	8104.7(18)	4653.3(8)	28.2(3)
C13	4864.9(11)	7904.0(14)	6128.5(6)	13.5(2)
C14	4019.0(12)	6431.6(15)	5291.2(6)	14.4(3)
C15	3459.9(11)	5924.5(14)	6325.6(6)	13.0(2)
C16	4367.3(11)	4743.5(14)	6452.4(6)	12.7(2)
C17	5276.3(12)	4953.1(14)	6894.2(6)	15.4(3)
C18	6172.9(12)	3933.7(15)	6972.0(6)	15.6(3)
C19	6172.7(11)	2672.7(14)	6615.6(6)	13.0(2)
C20	5245.9(11)	2427.1(14)	6189.7(6)	13.0(2)
C21	4350.7(12)	3452.7(14)	6110.2(6)	13.4(2)
C22	7155.0(12)	1603.8(14)	6740.3(6)	14.0(2)
C23	8260.7(14)	-473.5(18)	6384.1(7)	24.4(3)

Table S11 Anisotropic Displacement Parameters (Å^2×10^3) for ONW143s_0m. The Anisotropic displacement factor exponent takes the form: $-2\pi^2[h^2a^{*2}U_{11}+2hka^*b^*U_{12}+…]$.

Atom	U_{11}	U_{22}	U_{33}	U_{23}	U_{13}	U_{12}
Zn1	12.26(8)	11.73(8)	11.04(8)	0.28(5)	0.10(6)	2.84(5)
Cl1	25.89(17)	14.67(15)	12.91(15)	0.85(11)	-0.78(13)	-3.19(12)
Cl2	12.99(15)	23.14(17)	20.97(16)	3.78(13)	3.46(12)	3.35(12)
O1	22.0(5)	20.0(5)	17.3(5)	-1.8(4)	-8.0(4)	6.7(4)
N1	15.5(5)	15.3(5)	11.4(5)	-0.9(4)	1.0(4)	0.2(4)
N2	17.5(6)	13.3(5)	12.4(5)	-0.1(4)	-1.2(4)	1.5(4)
N3	11.1(5)	12.8(5)	10.7(5)	-0.6(4)	0.5(4)	0.8(4)
N4	17.2(6)	19.7(6)	12.6(5)	-0.3(4)	-2.0(4)	7.7(5)
C1	20.0(7)	18.1(6)	14.4(6)	-3.0(5)	4.4(5)	-1.0(5)
C2	27.9(8)	25.4(7)	15.4(7)	-7.2(6)	5.3(6)	-6.0(6)
C3	25.5(8)	29.6(8)	12.3(6)	-1.7(6)	-3.4(6)	-8.5(6)
C4	17.0(7)	21.2(7)	16.5(7)	2.8(5)	-3.3(5)	-3.9(5)
C5	15.3(6)	14.0(6)	12.9(6)	1.6(5)	0.9(5)	-1.7(5)
C6	25.2(8)	25.6(8)	26.3(8)	-9.5(6)	4.3(6)	5.8(6)
C7	20.6(7)	19.8(7)	14.7(6)	0.3(5)	-2.7(5)	1.9(5)
C8	22.4(7)	26.4(8)	20.6(7)	-5.5(6)	-4.9(6)	-2.4(6)
C9	31.2(8)	20.5(7)	23.4(8)	-10.4(6)	0.0(6)	-3.0(6)
C10	24.0(7)	17.6(7)	19.9(7)	-5.4(5)	2.3(6)	3.1(5)
C11	17.1(6)	15.1(6)	10.5(6)	-0.5(5)	2.8(5)	1.5(5)
C12	26.2(8)	28.2(8)	28.1(8)	-4.3(7)	-12.3(7)	10.8(6)
C13	10.7(6)	15.1(6)	14.6(6)	-0.4(5)	0.1(5)	0.8(5)
C14	15.1(6)	16.3(6)	11.9(6)	-2.0(5)	2.8(5)	3.0(5)
C15	13.1(6)	12.7(6)	13.2(6)	1.3(5)	1.7(5)	1.8(5)
C16	13.8(6)	12.1(6)	12.1(6)	1.8(5)	1.0(5)	1.5(5)
C17	19.9(7)	12.3(6)	13.7(6)	-1.8(5)	-1.4(5)	2.4(5)

C18	17.7(7)	15.1(6)	13.1(6)	-0.6(5)	-4.7(5)	1.8(5)
C19	14.4(6)	12.0(6)	12.4(6)	2.5(5)	0.5(5)	1.4(5)
C20	16.0(6)	10.3(6)	12.6(6)	-0.4(4)	0.0(5)	0.1(5)
C21	13.5(6)	13.1(6)	13.2(6)	0.7(5)	-2.5(5)	-0.6(5)
C22	14.8(6)	12.1(6)	15.0(6)	1.5(5)	-0.2(5)	1.7(5)
C23	25.7(8)	27.5(8)	19.7(7)	-2.6(6)	-0.8(6)	15.3(6)

Table S12 Bond Lengths for ONW143s_0m.

Atom	Atom	Length/Å	Atom	Atom	Length/Å
Zn1	Cl1	2.2898(7)	C3	C4	1.398(2)
Zn1	Cl2	2.2539(8)	C4	C5	1.389(2)
Zn1	N1	2.2421(13)	C5	C13	1.5169(19)
Zn1	N2	2.2925(13)	C7	C8	1.399(2)
Zn1	N3	2.1468(13)	C7	C12	1.506(2)
O1	C22	1.2344(17)	C8	C9	1.387(2)
N1	C1	1.3544(18)	C9	C10	1.395(2)
N1	C5	1.3516(18)	C10	C11	1.391(2)
N2	C7	1.3509(19)	C11	C14	1.515(2)
N2	C11	1.3509(17)	C15	C16	1.5161(18)
N3	C13	1.4847(17)	C16	C17	1.4015(19)
N3	C14	1.4849(17)	C16	C21	1.4036(19)
N3	C15	1.5126(17)	C17	C18	1.3935(19)
N4	C22	1.3513(18)	C18	C19	1.3992(19)
N4	C23	1.4594(18)	C19	C20	1.3982(19)
C1	C2	1.396(2)	C19	C22	1.5069(18)
C1	C6	1.507(2)	C20	C21	1.3965(18)
C2	C3	1.387(2)			

Table S13 Bond Angles for ONW143s_0m.

Atom	Atom	Atom	Angle/°	Atom	Atom	Atom	Angle/°
Cl1	Zn1	N2	94.72(4)	N1	C5	C4	122.85(13)
Cl2	Zn1	Cl1	137.149(16)	N1	C5	C13	114.75(11)
Cl2	Zn1	N2	92.09(4)	C4	C5	C13	122.32(13)
N1	Zn1	Cl1	93.88(4)	N2	C7	C8	121.07(13)
N1	Zn1	Cl2	96.74(4)	N2	C7	C12	117.73(13)
N1	Zn1	N2	155.98(4)	C8	C7	C12	121.20(14)
N3	Zn1	Cl1	111.49(4)	C9	C8	C7	119.81(14)
N3	Zn1	Cl2	111.32(4)	C8	C9	C10	119.01(14)
N3	Zn1	N1	77.86(5)	C11	C10	C9	118.31(14)
N3	Zn1	N2	78.12(5)	N2	C11	C10	122.77(13)
C1	N1	Zn1	129.67(10)	N2	C11	C14	114.42(12)

C5	N1	Zn1	111.11(9)	C10	C11	C14	122.80(12)
C5	N1	C1	119.04(12)	N3	C13	C5	109.94(11)
C7	N2	Zn1	128.49(9)	N3	C14	C11	110.73(11)
C11	N2	Zn1	110.26(9)	N3	C15	C16	113.23(11)
C11	N2	C7	118.98(12)	C17	C16	C15	120.49(12)
C13	N3	Zn1	104.56(8)	C17	C16	C21	118.57(12)
C13	N3	C14	111.06(10)	C21	C16	C15	120.86(12)
C13	N3	C15	112.13(10)	C18	C17	C16	120.61(12)
C14	N3	Zn1	106.37(8)	C17	C18	C19	120.54(12)
C14	N3	C15	111.56(10)	C18	C19	C22	117.24(12)
C15	N3	Zn1	110.78(8)	C20	C19	C18	119.20(12)
C22	N4	C23	121.12(12)	C20	C19	C22	123.47(12)
N1	C1	C2	121.06(14)	C21	C20	C19	120.19(12)
N1	C1	C6	117.47(13)	C20	C21	C16	120.80(12)
C2	C1	C6	121.46(13)	O1	C22	N4	122.39(12)
C3	C2	C1	119.58(14)	O1	C22	C19	120.72(12)
C2	C3	C4	119.44(13)	N4	C22	C19	116.89(12)
C5	C4	C3	117.97(14)				

Table S14 Hydrogen Bonds for ONW143s_0m.

D	H	A	d(D-H)/Å	d(H-A)/Å	d(D-A)/Å	D-H-A/°
N4	H4	Cl1[1]	0.88	2.65	3.3550(18)	137.5
C17	H17	O1[2]	0.95	2.71	3.2865(19)	120.0
C18	H18	O1[2]	0.95	2.61	3.2399(19)	124.3

[1]1-X,1-Y,1-Z; [2]3/2-X,1/2+Y,3/2-Z

Table S15 Torsion Angles for ONW143s_0m.

A	B	C	D	Angle/°	A	B	C	D	Angle/°
Zn1	N1	C1	C2	173.42(10)	C8	C9	C10	C11	0.5(2)
Zn1	N1	C1	C6	-7.79(19)	C9	C10	C11	N2	1.2(2)
Zn1	N1	C5	C4	-172.57(11)	C9	C10	C11	C14	-177.41(13)
Zn1	N1	C5	C13	10.63(13)	C10	C11	C14	N3	-134.99(13)
Zn1	N2	C7	C8	-159.12(11)	C11	N2	C7	C8	2.0(2)
Zn1	N2	C7	C12	21.22(19)	C11	N2	C7	C12	-177.69(13)
Zn1	N2	C11	C10	161.91(11)	C12	C7	C8	C9	179.29(15)
Zn1	N2	C11	C14	-19.41(13)	C13	N3	C14	C11	-160.99(11)
Zn1	N3	C13	C5	50.43(11)	C13	N3	C15	C16	-63.38(14)
Zn1	N3	C14	C11	-47.78(12)	C14	N3	C13	C5	164.78(10)

Zn1	N3	C15	C16	-179.78(8)	C14	N3	C15	C16	61.92(14)
N1	C1	C2	C3	-0.9(2)	C15	N3	C13	C5	-69.65(13)
N1	C5	C13	N3	-42.01(15)	C15	N3	C14	C11	73.12(13)
N2	C7	C8	C9	-0.4(2)	C15	C16	C17	C18	-173.75(12)
N2	C11	C14	N3	46.32(15)	C15	C16	C21	C20	174.04(12)
N3	C15	C16	C17	84.81(15)	C16	C17	C18	C19	-0.9(2)
N3	C15	C16	C21	-91.86(15)	C17	C16	C21	C20	-2.69(19)
C1	N1	C5	C4	3.0(2)	C17	C18	C19	C20	-1.6(2)
C1	N1	C5	C13	-173.79(12)	C17	C18	C19	C22	-178.25(12)
C1	C2	C3	C4	1.3(2)	C18	C19	C20	C21	1.92(19)
C2	C3	C4	C5	0.4(2)	C18	C19	C22	O1	19.34(19)
C3	C4	C5	N1	-2.6(2)	C18	C19	C22	N4	-161.45(12)
C3	C4	C5	C13	173.98(13)	C19	C20	C21	C16	0.3(2)
C4	C5	C13	N3	141.17(13)	C20	C19	C22	O1	-157.13(13)
C5	N1	C1	C2	-1.2(2)	C20	C19	C22	N4	22.08(19)
C5	N1	C1	C6	177.57(13)	C21	C16	C17	C18	3.0(2)
C6	C1	C2	C3	-179.63(14)	C22	C19	C20	C21	178.33(12)
C7	N2	C11	C10	-2.4(2)	C23	N4	C22	O1	0.7(2)
C7	N2	C11	C14	176.28(12)	C23	N4	C22	C19	-178.48(13)
C7	C8	C9	C10	-0.9(2)					

Table S16 Hydrogen Atom Coordinates (Å×10^4) and Isotropic Displacement Parameters (Å^2×10^3) for ONW143s_0m.

Atom	*x*	*y*	*z*	U(eq)
H4	6917.93	662.4	5960.4	20
H2	3704.38	11576.15	7760.93	27
H3	5451.85	10284.85	7933	27
H4A	5992.1	8549.83	7214.44	22
H6A	2014.23	11694.61	6414.45	38
H6B	2047.71	12224.03	7100.09	38
H6C	1426.33	10714.24	6905.83	38
H8	48.22	5449.81	4123.88	28
H9	1454	3593.01	4136.94	30
H10	3274.41	3988.78	4666.04	25
H12A	-405.75	7915.9	4962.6	42
H12B	-285.6	8156.15	4258.05	42
H12C	540.93	9035.51	4738.5	42
H13A	5164.05	8512.65	5805.39	16
H13B	5485	7212.74	6271.06	16

H14A	4505.13	5541.88	5344.97	17
H14B	4439.72	7142.84	5050.62	17
H15A	2730.12	5462.58	6160.13	16
H15B	3297.27	6406.24	6708.98	16
H17	5281.32	5797.94	7142.9	18
H18	6789.27	4096.22	7269.42	19
H20	5225.06	1559.98	5953.89	16
H21	3722.85	3274.77	5820.91	16
H23A	8445.03	-858.17	5991.2	37
H23B	7991.48	-1273.55	6632.58	37
H23C	8957.97	-29.81	6584.02	37

Crystal structure determination of ONW143s_0m

Crystal Data for $C_{23}H_{26}Cl_2N_4OZn$ (M =510.75 g/mol): monoclinic, space group P2$_1$/n (no. 14), a = 11.531(5) Å, b = 9.136(4) Å, c = 22.280(10) Å, β = 93.892(10)°, V = 2341.6(18) Å^3, Z = 4, T = 100.0 K, μ(MoKα) = 1.300 mm^{-1}, $Dcalc$ = 1.449 g/cm^3, 65388 reflections measured (3.664° ≤ 2Θ ≤ 57.276°), 5948 unique (R_{int} = 0.0300, R_{sigma} = 0.0174) which were used in all calculations. The final R_1 was 0.0244 (I > 2σ(I)) and wR_2 was 0.0614 (all data).

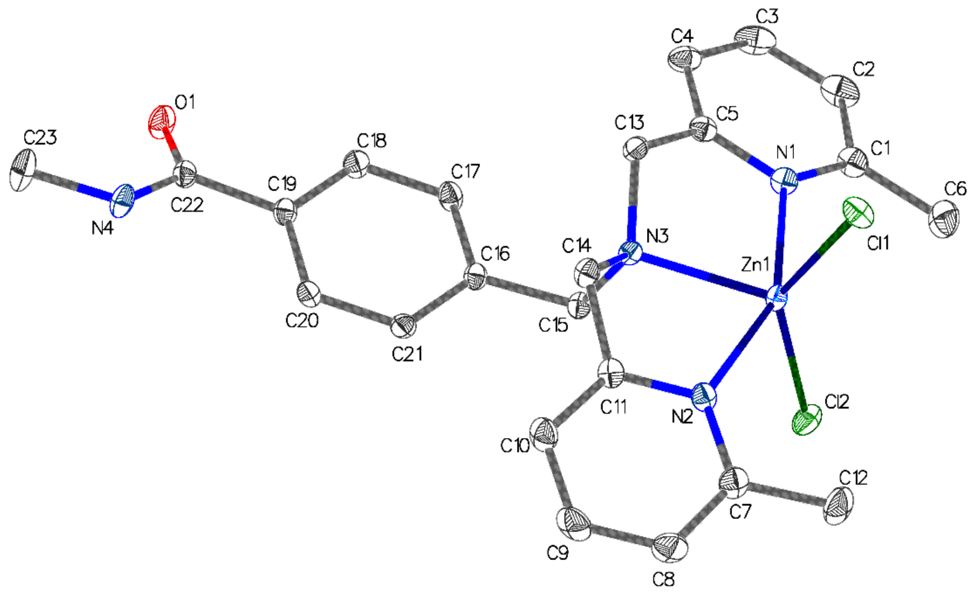

ONW146v_0m (14.Zn)

Table S17 Crystal data and structure refinement for ONW146v_0m.

Identification code	ONW146v_0m
Empirical formula	$C_{22}H_{28}Cl_2N_6O_2Zn$
Formula weight	544.77
Temperature/K	100.0
Crystal system	triclinic
Space group	P-1
a/Å	10.4293(5)
b/Å	15.2887(7)
c/Å	16.4734(8)
α/°	101.862(2)
β/°	90.335(2)
γ/°	92.981(2)
Volume/Å^3	2566.8(2)
Z	4
ρ_{calc}g/cm^3	1.410
μ/mm^{-1}	3.493
F(000)	1128.0
Crystal size/mm^3	0.35 × 0.28 × 0.068
Radiation	CuKα (λ = 1.54178)
2Θ range for data collection/°	5.482 to 133.57
Index ranges	-12 ≤ h ≤ 12, -17 ≤ k ≤ 18, -19 ≤ l ≤ 19
Reflections collected	101989
Independent reflections	9030 [R_{int} = 0.0585, R_{sigma} = 0.0240]
Data/restraints/parameters	9030/0/601
Goodness-of-fit on F^2	1.040
Final R indexes [I>=2σ (I)]	R_1 = 0.0589, wR_2 = 0.1595
Final R indexes [all data]	R_1 = 0.0625, wR_2 = 0.1642
Largest diff. peak/hole / e Å^{-3}	3.00/-0.75

Table S18 Fractional Atomic Coordinates (×10^4) and Equivalent Isotropic Displacement Parameters (Å^2×10^3) for ONW146v_0m. U_{eq} is defined as 1/3 of of the trace of the orthogonalised U_{IJ} tensor.

Atom	*x*	*y*	*z*	U(eq)
Zn1	-904.4(4)	8219.1(3)	3707.5(2)	17.86(14)
Cl1	-1691.7(13)	9570.0(6)	4283.7(7)	57.6(4)
Cl2	-2221.1(7)	7231.9(6)	2805.3(5)	27.6(2)
O1	5569(2)	4345.1(15)	4001.0(14)	26.3(5)

N1	-1304(2)	7586.2(16)	4765.9(15)	18.7(5)
N2	321(3)	8665.1(16)	2772.2(16)	21.1(5)
N3	930(2)	7762.6(16)	3963.6(15)	17.5(5)
N4	6789(3)	5442.0(18)	3607.7(17)	24.4(6)
N5	-3521(3)	7611.8(19)	4696.4(17)	25.3(6)
N6	-1302(3)	8977.8(19)	1931.3(18)	30.5(7)
C1	-2466(3)	7348(2)	5032.6(19)	20.1(6)
C2	-2584(3)	6842(2)	5657.6(19)	22.4(6)
C3	-1488(3)	6598(2)	5999.8(19)	24.0(7)
C4	-276(3)	6866(2)	5743.8(19)	23.7(7)
C5	-245(3)	7354.0(19)	5133.0(18)	19.0(6)
C6	-26(4)	8927(2)	2078.9(19)	25.9(7)
C7	884(4)	9127(2)	1505(2)	32.2(8)
C8	2154(4)	9040(2)	1655(2)	33.8(9)
C9	2527(4)	8769(2)	2379(2)	29.7(8)
C10	1597(3)	8612(2)	2918(2)	22.6(7)
C11	1009(3)	7734(2)	4851.0(18)	18.8(6)
C12	1921(3)	8398(2)	3748.8(19)	19.3(6)
C13	978(3)	6851.6(19)	3409.2(18)	17.7(6)
C14	2202(3)	6384(2)	3468.0(18)	19.6(6)
C15	2276(3)	5790(2)	4004.4(19)	20.2(6)
C16	3403(3)	5366(2)	4083.4(19)	21.5(6)
C17	4480(3)	5540(2)	3636.1(19)	19.6(6)
C18	4408(3)	6119(2)	3086.5(19)	20.7(6)
C19	3273(3)	6527(2)	2999.5(18)	20.3(6)
C20	5663(3)	5059(2)	3763.3(18)	20.2(6)
C21	7988(3)	5042(2)	3722(2)	28.9(7)
Zn2	-627.2(4)	1812.9(3)	1282.6(2)	17.56(14)
Cl3	-1628.3(12)	467.8(6)	689.5(6)	52.4(3)
Cl4	-1786.9(7)	2755.4(5)	2229.1(5)	26.22(19)
O2	6617(2)	5598.7(15)	1002.6(14)	24.9(5)
N7	-980(2)	2484.9(16)	254.2(15)	17.5(5)
N8	559(3)	1348.6(16)	2191.8(16)	20.6(5)
N9	1273(2)	2275.4(16)	1017.1(15)	16.6(5)
N10	7537(2)	4553.0(17)	1552.6(16)	21.8(5)
N11	-3202(3)	2480.5(18)	338.4(17)	23.7(6)
N12	-1080(3)	1025.0(18)	3046.3(17)	27.4(6)
C22	-2107(3)	2742(2)	1.4(18)	19.5(6)
C23	-2159(3)	3270(2)	-604.9(19)	22.1(6)
C24	-1040(3)	3504(2)	-957(2)	24.5(7)
C25	132(3)	3223(2)	-713.9(19)	22.9(6)
C26	101(3)	2715(2)	-115.5(18)	18.7(6)
C27	185(3)	1077(2)	2890.2(19)	24.3(7)
C28	1092(4)	858(2)	3445(2)	30.3(8)
C29	2367(4)	933(2)	3285(2)	31.2(8)

C30	2763(3)	1212(2)	2563(2)	27.4(7)
C31	1830(3)	1398.8(19)	2042.3(19)	20.4(6)
C32	1308(3)	2320(2)	131.5(18)	20.9(6)
C33	2156(3)	1622(2)	1211.1(19)	19.8(6)
C34	1487(3)	3177.6(19)	1573.6(18)	18.3(6)
C35	2788(3)	3643.6(19)	1521.9(18)	18.0(6)
C36	2977(3)	4262(2)	1013.9(19)	20.3(6)
C37	4178(3)	4680(2)	960.1(19)	20.9(6)
C38	5229(3)	4464.9(19)	1399.1(18)	18.2(6)
C39	5043(3)	3859(2)	1916.2(18)	19.7(6)
C40	3835(3)	3463(2)	1984.5(18)	19.6(6)
C41	6516(3)	4920(2)	1301.7(18)	19.5(6)
C42	8819(3)	4947(2)	1505(2)	26.2(7)
O3	4050(2)	7208.2(16)	5441.9(16)	30.2(5)
C43	4073(4)	7849(3)	6187(3)	39.5(9)
O4	5415(2)	7007.5(18)	532.5(16)	35.5(6)
C44	5551(4)	7702(3)	1228(2)	39.1(9)

Table S19 Anisotropic Displacement Parameters ($Å^2 \times 10^3$) for ONW146v_0m. The Anisotropic displacement factor exponent takes the form: $-2\pi^2[h^2a^{*2}U_{11}+2hka^*b^*U_{12}+\ldots]$.

Atom	U_{11}	U_{22}	U_{33}	U_{23}	U_{13}	U_{12}
Zn1	23.9(2)	14.8(2)	15.7(2)	4.13(16)	2.81(16)	4.29(16)
Cl1	108.5(10)	23.0(5)	49.2(6)	18.2(4)	51.6(6)	28.8(5)
Cl2	27.3(4)	36.0(5)	19.7(4)	7.9(3)	-4.3(3)	-5.7(3)
O1	31.2(12)	21.3(12)	29.2(12)	10.2(9)	1.9(10)	5.6(9)
N1	26.6(13)	14.8(12)	13.5(12)	0.7(9)	2.2(10)	0.3(10)
N2	32.8(14)	11.6(12)	18.0(13)	1.5(10)	1.9(11)	-0.2(10)
N3	20.5(12)	13.3(12)	17.9(12)	1.7(10)	3.3(10)	-0.4(9)
N4	26.9(14)	16.1(13)	29.4(15)	2.4(11)	-0.8(11)	1.4(10)
N5	23.6(13)	27.7(15)	27.6(14)	12.3(11)	4.7(11)	3.2(11)
N6	48.1(18)	24.4(15)	21.3(14)	10.4(11)	-3.9(13)	0.8(13)
C1	25.9(15)	14.3(15)	18.1(15)	-1.2(11)	3.1(12)	0.6(11)
C2	30.0(16)	17.0(15)	19.5(15)	2.4(12)	8.4(13)	-2.7(12)
C3	36.6(18)	18.6(16)	17.1(15)	4.8(12)	2.7(13)	-0.7(13)
C4	30.2(17)	20.9(16)	19.0(15)	1.6(12)	-2.9(13)	1.9(13)
C5	29.2(16)	10.6(14)	15.0(14)	-3.4(11)	0.2(12)	4.0(11)
C6	49(2)	10.8(15)	16.4(15)	0.2(11)	1.6(14)	1.1(13)
C7	66(3)	12.0(16)	18.0(16)	1.9(12)	7.1(16)	-1.3(15)
C8	59(2)	13.2(16)	27.9(18)	2.4(13)	20.5(17)	-0.9(15)
C9	41(2)	17.3(16)	30.3(18)	4.6(13)	15.8(15)	0.5(13)
C10	32.8(17)	12.5(15)	21.3(16)	1.1(12)	7.0(13)	-0.1(12)
C11	26.0(15)	13.2(14)	15.4(14)	-1.1(11)	-0.5(12)	1.5(11)
C12	22.8(15)	14.5(15)	20.1(15)	3.7(11)	1.2(12)	-5.2(11)

C13	22.4(15)	14.2(14)	15.5(14)	0.7(11)	0.5(11)	0.8(11)
C14	22.9(15)	16.8(15)	17.0(14)	-1.1(11)	-1.4(12)	0.9(11)
C15	23.1(15)	14.6(15)	22.8(15)	4.1(12)	4.7(12)	-0.3(11)
C16	28.5(16)	15.5(15)	20.4(15)	3.8(12)	-1.6(12)	1.1(12)
C17	22.9(15)	14.3(15)	19.5(15)	-0.7(11)	-1.4(12)	0.9(11)
C18	23.4(15)	17.3(15)	20.0(15)	0.1(12)	3.5(12)	2.4(12)
C19	27.5(16)	17.6(15)	16.5(14)	4.0(11)	3.4(12)	3.9(12)
C20	28.3(16)	18.4(16)	12.2(14)	-1.6(11)	-1.2(12)	4.3(12)
C21	27.4(17)	28.0(18)	29.6(18)	1.7(14)	-3.4(14)	1.7(14)
Zn2	22.5(2)	14.8(2)	15.7(2)	4.31(16)	-1.55(16)	-1.09(16)
Cl3	93.0(8)	22.8(5)	42.0(5)	15.8(4)	-40.5(5)	-22.6(5)
Cl4	30.3(4)	31.9(4)	19.8(4)	10.4(3)	6.3(3)	11.9(3)
O2	27.3(11)	22.0(12)	28.1(12)	12.9(9)	-4.6(9)	-2.8(9)
N7	23.4(13)	14.0(12)	14.9(12)	2.2(9)	-1.0(10)	0.8(9)
N8	32.2(14)	11.0(12)	18.5(13)	2.8(10)	-1.4(11)	1.8(10)
N9	18.8(12)	13.8(12)	16.3(12)	1.2(9)	-0.6(9)	-0.1(9)
N10	21.7(13)	18.1(13)	26.6(14)	7.2(11)	2.6(11)	0.8(10)
N11	20.0(13)	28.5(15)	24.7(14)	10.7(11)	-0.7(11)	-0.3(11)
N12	42.9(17)	20.9(14)	19.7(14)	8.0(11)	4.2(12)	-0.5(12)
C22	23.7(15)	17.1(15)	16.6(14)	1.1(11)	-3.1(12)	0.3(11)
C23	26.4(16)	18.1(15)	21.1(15)	2.7(12)	-2.4(12)	1.7(12)
C24	32.3(17)	22.1(16)	20.5(16)	7.9(12)	-3.0(13)	0.1(13)
C25	27.6(16)	22.2(16)	19.8(15)	6.3(12)	2.3(12)	0.0(12)
C26	25.1(15)	14.9(15)	14.5(14)	-0.3(11)	-0.4(11)	1.2(11)
C27	45(2)	8.5(14)	18.3(15)	1.1(11)	0.9(14)	1.5(12)
C28	61(2)	10.9(15)	18.3(16)	3.0(12)	-6.5(15)	-1.4(14)
C29	51(2)	13.6(16)	27.9(18)	2.4(13)	-16.2(16)	2.5(14)
C30	36.1(18)	16.9(16)	28.1(17)	2.0(13)	-11.3(14)	2.1(13)
C31	29.8(16)	9.4(14)	20.4(15)	-0.3(11)	-5.3(12)	0.6(11)
C32	23.3(15)	23.2(16)	16.2(15)	3.9(12)	-0.1(12)	2.8(12)
C33	22.9(15)	16.0(15)	19.9(15)	1.4(11)	-0.7(12)	4.6(11)
C34	21.3(15)	15.3(15)	17.1(14)	0.8(11)	-0.5(11)	0.4(11)
C35	22.1(15)	13.6(14)	16.8(14)	-0.9(11)	0.8(11)	2.6(11)
C36	22.3(15)	16.8(15)	21.1(15)	1.6(12)	-2.2(12)	2.8(11)
C37	27.7(16)	16.6(15)	18.5(15)	3.3(12)	-0.4(12)	2.0(12)
C38	22.9(15)	14.2(15)	16.3(14)	-0.1(11)	-0.2(11)	1.7(11)
C39	23.4(15)	19.4(15)	15.5(14)	1.4(11)	-0.4(12)	2.4(12)
C40	25.5(15)	17.8(15)	15.0(14)	3.4(11)	-0.6(12)	-2.1(12)
C41	25.7(16)	16.2(15)	15.0(14)	-0.3(11)	-0.6(11)	0.9(12)
C42	23.8(16)	23.5(17)	31.3(18)	5.5(13)	2.1(13)	0.9(13)
O3	29.7(12)	25.8(13)	35.8(14)	8.2(10)	0.7(10)	1.2(10)
C43	38(2)	40(2)	39(2)	4.9(17)	6.4(17)	0.1(16)
O4	33.2(14)	35.5(15)	38.1(14)	6.0(11)	-9.0(11)	11.1(11)
C44	37(2)	41(2)	37(2)	3.3(17)	-6.3(16)	9.6(17)

Table S20 Bond Lengths for ONW146v_0m.

Atom	Atom	Length/Å	Atom	Atom	Length/Å
Zn1	Cl1	2.2849(9)	Zn2	Cl4	2.2913(8)
Zn1	Cl2	2.2827(8)	Zn2	N7	2.193(2)
Zn1	N1	2.193(3)	Zn2	N8	2.188(3)
Zn1	N2	2.199(3)	Zn2	N9	2.147(2)
Zn1	N3	2.139(3)	O2	C41	1.236(4)
O1	C20	1.233(4)	N7	C22	1.349(4)
N1	C1	1.349(4)	N7	C26	1.349(4)
N1	C5	1.354(4)	N8	C27	1.354(4)
N2	C6	1.340(4)	N8	C31	1.350(4)
N2	C10	1.360(4)	N9	C32	1.475(4)
N3	C11	1.473(4)	N9	C33	1.474(4)
N3	C12	1.476(4)	N9	C34	1.496(4)
N3	C13	1.504(4)	N10	C41	1.332(4)
N4	C20	1.334(4)	N10	C42	1.447(4)
N4	C21	1.449(4)	N11	C22	1.351(4)
N5	C1	1.345(4)	N12	C27	1.347(5)
N6	C6	1.360(5)	C22	C23	1.410(4)
C1	C2	1.412(4)	C23	C24	1.369(5)
C2	C3	1.373(5)	C24	C25	1.400(5)
C3	C4	1.403(5)	C25	C26	1.374(4)
C4	C5	1.370(4)	C26	C32	1.513(4)
C5	C11	1.517(4)	C27	C28	1.411(5)
C6	C7	1.410(5)	C28	C29	1.361(6)
C7	C8	1.365(6)	C29	C30	1.402(5)
C8	C9	1.399(5)	C30	C31	1.372(5)
C9	C10	1.363(5)	C31	C33	1.514(4)
C10	C12	1.512(4)	C34	C35	1.510(4)
C13	C14	1.509(4)	C35	C36	1.393(4)
C14	C15	1.395(4)	C35	C40	1.400(4)
C14	C19	1.394(4)	C36	C37	1.389(4)
C15	C16	1.389(4)	C37	C38	1.399(4)
C16	C17	1.391(4)	C38	C39	1.388(4)
C17	C18	1.395(4)	C38	C41	1.505(4)
C17	C20	1.503(4)	C39	C40	1.385(4)
C18	C19	1.386(4)	O3	C43	1.404(5)
Zn2	Cl3	2.2830(9)	O4	C44	1.394(5)

Table S21 Bond Angles for ONW146v_0m.

Atom	Atom	Atom	Angle/°	Atom	Atom	Atom	Angle/°
Cl2	Zn1	Cl1	117.29(5)	Cl3	Zn2	Cl4	116.38(4)
N1	Zn1	Cl1	96.17(7)	N7	Zn2	Cl3	96.65(7)
N1	Zn1	Cl2	94.64(7)	N7	Zn2	Cl4	95.26(7)
N1	Zn1	N2	154.88(10)	N8	Zn2	Cl3	98.00(7)
N2	Zn1	Cl1	98.07(7)	N8	Zn2	Cl4	96.17(7)
N2	Zn1	Cl2	97.06(7)	N8	Zn2	N7	155.05(10)
N3	Zn1	Cl1	126.37(8)	N9	Zn2	Cl3	125.98(8)
N3	Zn1	Cl2	116.29(7)	N9	Zn2	Cl4	117.64(7)
N3	Zn1	N1	77.56(10)	N9	Zn2	N7	77.68(9)
N3	Zn1	N2	77.33(10)	N9	Zn2	N8	77.36(10)
C1	N1	Zn1	127.1(2)	C22	N7	Zn2	128.1(2)
C1	N1	C5	118.3(3)	C22	N7	C26	118.1(3)
C5	N1	Zn1	114.3(2)	C26	N7	Zn2	113.6(2)
C6	N2	Zn1	128.9(2)	C27	N8	Zn2	128.3(2)
C6	N2	C10	117.7(3)	C31	N8	Zn2	113.8(2)
C10	N2	Zn1	113.4(2)	C31	N8	C27	117.8(3)
C11	N3	Zn1	108.66(18)	C32	N9	Zn2	108.14(18)
C11	N3	C12	111.0(2)	C32	N9	C34	112.3(2)
C11	N3	C13	112.7(2)	C33	N9	Zn2	106.72(18)
C12	N3	Zn1	107.64(18)	C33	N9	C32	111.5(2)
C12	N3	C13	111.7(2)	C33	N9	C34	112.4(2)
C13	N3	Zn1	104.80(17)	C34	N9	Zn2	105.29(17)
C20	N4	C21	121.4(3)	C41	N10	C42	121.1(3)
N1	C1	C2	121.2(3)	N7	C22	N11	118.7(3)
N5	C1	N1	118.6(3)	N7	C22	C23	121.4(3)
N5	C1	C2	120.2(3)	N11	C22	C23	119.9(3)
C3	C2	C1	118.8(3)	C24	C23	C22	118.9(3)
C2	C3	C4	120.3(3)	C23	C24	C25	120.2(3)
C5	C4	C3	117.2(3)	C26	C25	C24	117.3(3)
N1	C5	C4	124.1(3)	N7	C26	C25	124.1(3)
N1	C5	C11	114.1(3)	N7	C26	C32	115.2(3)
C4	C5	C11	121.6(3)	C25	C26	C32	120.7(3)
N2	C6	N6	117.7(3)	N8	C27	C28	121.1(3)
N2	C6	C7	121.9(3)	N12	C27	N8	118.3(3)
N6	C6	C7	120.3(3)	N12	C27	C28	120.6(3)
C8	C7	C6	119.0(3)	C29	C28	C27	119.7(3)
C7	C8	C9	119.5(3)	C28	C29	C30	119.5(3)
C10	C9	C8	118.3(4)	C31	C30	C29	117.8(3)
N2	C10	C9	123.5(3)	N8	C31	C30	124.1(3)
N2	C10	C12	114.6(3)	N8	C31	C33	114.3(3)
C9	C10	C12	121.9(3)	C30	C31	C33	121.5(3)
N3	C11	C5	110.6(2)	N9	C32	C26	110.1(2)
N3	C12	C10	109.9(2)	N9	C33	C31	109.9(2)

N3	C13	C14	115.2(2)	N9	C34	C35	115.8(2)
C15	C14	C13	119.5(3)	C36	C35	C34	121.0(3)
C19	C14	C13	121.8(3)	C36	C35	C40	118.4(3)
C19	C14	C15	118.7(3)	C40	C35	C34	120.6(3)
C16	C15	C14	120.6(3)	C37	C36	C35	120.8(3)
C15	C16	C17	120.3(3)	C36	C37	C38	120.2(3)
C16	C17	C18	119.5(3)	C37	C38	C41	118.0(3)
C16	C17	C20	116.8(3)	C39	C38	C37	119.2(3)
C18	C17	C20	123.6(3)	C39	C38	C41	122.7(3)
C19	C18	C17	119.9(3)	C40	C39	C38	120.3(3)
C18	C19	C14	121.0(3)	C39	C40	C35	121.0(3)
O1	C20	N4	122.8(3)	O2	C41	N10	121.9(3)
O1	C20	C17	120.2(3)	O2	C41	C38	121.6(3)
N4	C20	C17	116.9(3)	N10	C41	C38	116.5(3)

Table S22 Hydrogen Bonds for ONW146v_0m.

D	H	A	d(D-H)/Å	d(H-A)/Å	d(D-A)/Å	D-H-A/°
N5	H5B	O3[1]	0.88	2.04	2.914(4)	170.4
N10	H10	Cl4[2]	0.88	2.47	3.278(3)	153.4
N11	H11D	O4[3]	0.88	2.08	2.934(4)	162.4
O3	H3A	O1[4]	0.84	1.94	2.762(3)	165.2
O4	H4B	O2	0.84	1.95	2.787(3)	171.7

[1]-1+X,+Y,+Z; [2]1+X,+Y,+Z; [3]-X,1-Y,-Z; [4]1-X,1-Y,1-Z

Table S23 Torsion Angles for ONW146v_0m.

A	B	C	D	Angle/°	A	B	C	D	Angle/°
Zn1	N1	C1	N5	-9.0(4)	Zn2	N7	C22	N11	-8.5(4)
Zn1	N1	C1	C2	171.8(2)	Zn2	N7	C22	C23	171.8(2)
Zn1	N1	C5	C4	-172.7(2)	Zn2	N7	C26	C25	-172.9(2)
Zn1	N1	C5	C11	11.2(3)	Zn2	N7	C26	C32	11.0(3)
Zn1	N2	C6	N6	3.8(4)	Zn2	N8	C27	N12	4.3(4)
Zn1	N2	C6	C7	-174.8(2)	Zn2	N8	C27	C28	-175.3(2)
Zn1	N2	C10	C9	173.4(2)	Zn2	N8	C31	C30	174.6(2)
Zn1	N2	C10	C12	-8.9(3)	Zn2	N8	C31	C33	-9.3(3)
Zn1	N3	C11	C5	42.0(3)	Zn2	N9	C32	C26	42.4(3)
Zn1	N3	C12	C10	-45.3(3)	Zn2	N9	C33	C31	-46.0(3)
Zn1	N3	C13	C14	179.4(2)	Zn2	N9	C34	C35	178.5(2)
N1	C1	C2	C3	0.5(4)	N7	C22	C23	C24	1.8(5)
N1	C5	C11	N3	-35.7(3)	N7	C26	C32	N9	-36.2(3)
N2	C6	C7	C8	0.8(5)	N8	C27	C28	C29	1.0(5)

N2	C10	C12	N3	36.5(3)	N8	C31	C33	N9	37.6(3)
N3	C13	C14	C15	93.9(3)	N9	C34	C35	C36	95.3(3)
N3	C13	C14	C19	-86.1(3)	N9	C34	C35	C40	-84.7(3)
N5	C1	C2	C3	-178.7(3)	N11	C22	C23	C24	-177.9(3)
N6	C6	C7	C8	-177.7(3)	N12	C27	C28	C29	-178.6(3)
C1	N1	C5	C4	2.1(4)	C22	N7	C26	C25	2.4(4)
C1	N1	C5	C11	-174.0(2)	C22	N7	C26	C32	-173.6(3)
C1	C2	C3	C4	1.4(5)	C22	C23	C24	C25	-0.2(5)
C2	C3	C4	C5	-1.6(4)	C23	C24	C25	C26	-0.2(5)
C3	C4	C5	N1	-0.2(5)	C24	C25	C26	N7	-0.9(5)
C3	C4	C5	C11	175.7(3)	C24	C25	C26	C32	174.9(3)
C4	C5	C11	N3	148.1(3)	C25	C26	C32	N9	147.6(3)
C5	N1	C1	N5	177.0(3)	C26	N7	C22	N11	176.9(3)
C5	N1	C1	C2	-2.2(4)	C26	N7	C22	C23	-2.8(4)
C6	N2	C10	C9	-3.3(4)	C27	N8	C31	C30	-1.6(4)
C6	N2	C10	C12	174.5(3)	C27	N8	C31	C33	174.4(2)
C6	C7	C8	C9	-1.0(5)	C27	C28	C29	C30	-1.1(5)
C7	C8	C9	C10	-0.8(5)	C28	C29	C30	C31	-0.1(5)
C8	C9	C10	N2	3.1(5)	C29	C30	C31	N8	1.5(5)
C8	C9	C10	C12	-174.5(3)	C29	C30	C31	C33	-174.3(3)
C9	C10	C12	N3	-145.7(3)	C30	C31	C33	N9	-146.2(3)
C10	N2	C6	N6	179.8(3)	C31	N8	C27	N12	179.9(3)
C10	N2	C6	C7	1.3(4)	C31	N8	C27	C28	0.3(4)
C11	N3	C12	C10	-164.1(2)	C32	N9	C33	C31	-163.9(2)
C11	N3	C13	C14	-62.6(3)	C32	N9	C34	C35	-64.1(3)
C12	N3	C11	C5	160.2(2)	C33	N9	C32	C26	159.5(2)
C12	N3	C13	C14	63.2(3)	C33	N9	C34	C35	62.7(3)
C13	N3	C11	C5	-73.7(3)	C34	N9	C32	C26	-73.3(3)
C13	N3	C12	C10	69.2(3)	C34	N9	C33	C31	68.9(3)
C13	C14	C15	C16	-178.5(3)	C34	C35	C36	C37	-179.1(3)
C13	C14	C19	C18	177.3(3)	C34	C35	C40	C39	177.4(3)
C14	C15	C16	C17	0.9(5)	C35	C36	C37	C38	1.9(5)
C15	C14	C19	C18	-2.6(4)	C36	C35	C40	C39	-2.6(4)
C15	C16	C17	C18	-2.1(4)	C36	C37	C38	C39	-2.8(4)
C15	C16	C17	C20	179.5(3)	C36	C37	C38	C41	178.5(3)
C16	C17	C18	C19	0.9(4)	C37	C38	C39	C40	1.1(4)
C16	C17	C20	O1	24.6(4)	C37	C38	C41	O2	15.9(4)
C16	C17	C20	N4	-155.4(3)	C37	C38	C41	N10	-164.0(3)
C17	C18	C19	C14	1.5(5)	C38	C39	C40	C35	1.6(5)

C18	C17	C20	O1	-153.7(3)	C39	C38	C41	O2	-162.8(3)
C18	C17	C20	N4	26.3(4)	C39	C38	C41	N10	17.4(4)
C19	C14	C15	C16	1.5(4)	C40	C35	C36	C37	0.8(4)
C20	C17	C18	C19	179.2(3)	C41	C38	C39	C40	179.7(3)
C21	N4	C20	O1	-0.9(5)	C42	N10	C41	O2	1.6(5)
C21	N4	C20	C17	179.1(3)	C42	N10	C41	C38	-178.6(3)

Table S24 Hydrogen Atom Coordinates ($Å\times10^4$) and Isotropic Displacement Parameters ($Å^2\times10^3$) for ONW146v_0m.

Atom	*x*	*y*	*z*	U(eq)
H4	6803.12	5948.33	3431.8	29
H5A	-3443.65	7932.73	4311.23	30
H5B	-4287.61	7463.43	4861.76	30
H6A	-1867.4	8840.52	2285.67	37
H6B	-1561.61	9147.83	1481.38	37
H2	-3405.98	6673.09	5838.23	27
H3	-1551.72	6245.82	6411.98	29
H4A	491.73	6716.08	5984.11	28
H7	616.97	9318.89	1020.22	39
H8	2781.3	9161.78	1271.47	41
H9	3404.63	8697.05	2491.96	36
H11A	1199.04	8345.79	5180.75	23
H11B	1716.49	7357.93	4946.5	23
H12A	2769.22	8132.97	3725.1	23
H12B	1966.75	8954.79	4180.92	23
H13A	852.17	6912.66	2827.67	21
H13B	250.16	6467.67	3545.3	21
H15	1548.37	5674.09	4318.42	24
H16	3438.36	4956.78	4444.35	26
H18	5134.98	6234.61	2772.34	25
H19	3223.73	6910.02	2614.78	24
H21A	8666.03	5295.79	3416.53	43
H21B	7889.99	4393.12	3513.46	43
H21C	8219.33	5164.93	4313.66	43
H10	7430.71	4061.97	1750.36	26
H11C	-3171.88	2148.62	715.04	28
H11D	-3946.7	2641.92	181.13	28
H12C	-1638.78	1166.17	2698.52	33
H12D	-1347.59	850.09	3496	33
H23	-2956.36	3461.21	-766.33	26
H24	-1059.53	3857.83	-1367.93	29
H25	916.65	3376.71	-952.95	28

H28	814.08	659.02	3928.08	36
H29	2984.78	796.59	3660.21	37
H30	3647.25	1270.82	2437.53	33
H32A	1380.73	1711.61	-209.82	25
H32B	2069.51	2694.59	29.36	25
H33A	3051.8	1874.25	1226.92	24
H33B	2084.64	1070.22	773.2	24
H34A	1360.29	3111.74	2153.59	22
H34B	821.73	3568.24	1441.81	22
H36	2274.97	4399.89	700.6	24
H37	4287.76	5113.86	624.15	25
H39	5746.84	3716.81	2224.44	24
H40	3715.16	3061.11	2351.2	24
H42A	9051.83	4892.86	922.45	39
H42B	9428.84	4635.19	1783.49	39
H42C	8843.99	5581.33	1778.6	39
H3A	4063.74	6693.99	5547.26	45
H43A	3362.7	7710.39	6534.57	59
H43B	3983.67	8444.19	6065.73	59
H43C	4890.35	7839.71	6481.55	59
H4B	5785.42	6561.04	622.53	53
H44A	5253.57	8252.37	1091.14	59
H44B	6456.1	7792.89	1402.2	59
H44C	5037.23	7547.57	1680.26	59

Table S25 Solvent masks information for ONW146v_0m.

Number	X	Y	Z	Volume	Electron count	Content
1	0.500	0.000	-0.949	296.3	67.2	?

Crystal structure determination of ONW146v_0m

Crystal Data for $C_{22}H_{28}Cl_2N_6O_2Zn$ (M =544.77 g/mol): triclinic, space group P-1 (no. 2), a = 10.4293(5) Å, b = 15.2887(7) Å, c = 16.4734(8) Å, α = 101.862(2)°, β = 90.335(2)°, γ = 92.981(2)°, V = 2566.8(2) Å^3, Z = 4, T = 100.0 K, μ(CuKα) = 3.493 mm^{-1}, $Dcalc$ = 1.410 g/cm^3, 101989 reflections measured (5.482° ≤ 2Θ ≤ 133.57°), 9030 unique (R_{int} = 0.0585, R_{sigma} = 0.0240) which were used in all calculations. The final R_1 was 0.0589 (I > 2σ(I)) and wR_2 was 0.1642 (all data).

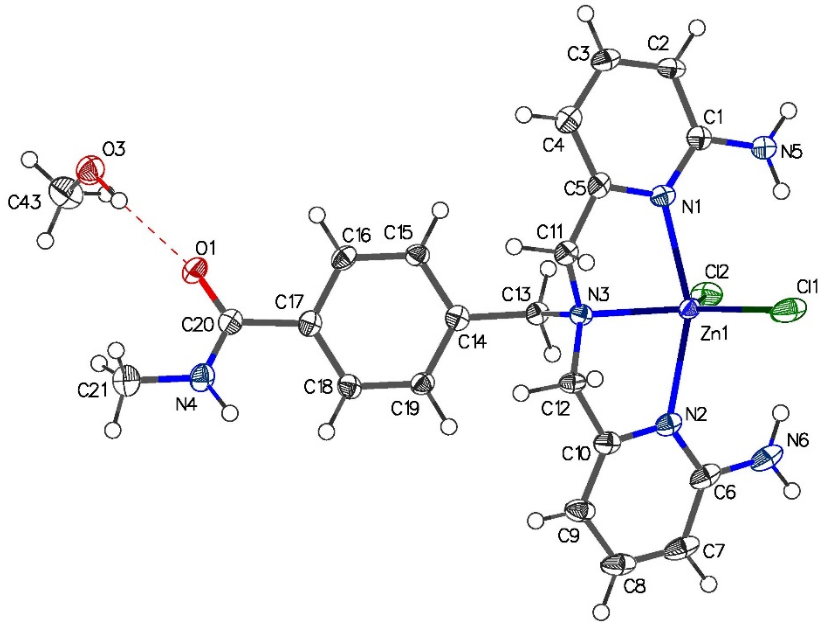
O3
C43
O1
C16
C15
C11
Cl2
Cl1
C17
C13
N3
C20
C14
Zn1
C21
N4
C18
C19
C12
N2
N6
C10
C6
C9
C7
C8
C3
C2
C1
C4
N5
C5
N1
